# Olaparib maintenance versus placebo monotherapy in patients with advanced non-small cell lung cancer (PIN): A multicentre, randomised, controlled, phase 2 trial

**DOI:** 10.1016/j.eclinm.2022.101595

**Published:** 2022-08-11

**Authors:** Dean A. Fennell, Catharine Porter, Jason Lester, Sarah Danson, Fiona Blackhall, Marianne Nicolson, Lisette Nixon, Georgina Gardner, Ann White, Gareth Griffiths, Angela Casbard

**Affiliations:** a University of Leicester & University Hospitals of Leicester NHS Trust, Leicester, UK; b Centre for Trials Research, Cardiff University, South Wales, UK; c Rutherford Hospitals, Newport, South Wales, UK; d Western Park Hospital NHS Trust, Sheffield, UK; e Christie NHS Foundation Trust, Manchester UK; f Aberdeen Royal Infirmary, Scotland, UK; g University of Southampton, UK

**Keywords:** NSCLC, PARP, Olaparib, HRD, Placebo, Randomised, Maintenance

## Abstract

**Background:**

Impaired double strand DNA repair by homologous repair deficiency (HRD) leads to sensitivity to poly ADP ribose polymerase (PARP) inhibition. Poly-ADP ribose polymerase (PARP) inhibitors target HRD to induce synthetic lethality and are used routinely in the treatment of BRCA1 mutated ovarian cancer in the platinum-sensitive maintenance setting. A subset of non-small cell lung cancers (NSCLCs) harbour impaired DNA double strand break repair. We therefore hypothesised that patients with metastatic non-small cell lung cancer exhibiting partial responses to platinum doublet-based chemotherapy, might enrich for impaired HRD, rendering these tumours more sensitive to inhibition of PARP inhibition by olaparib.

**Methods:**

The Olaparib Maintenance versus Placebo Monotherapy in Patients with Advanced Non-Small Cell Lung Cancer trial (PIN) was a multicentre double-blind placebo controlled randomised phase II screening trial. This study was conducted at 23 investigative hospital sites in the UK. Patients had advanced (stage IIIB/IV) squamous (Sq) or non-squamous (NSq) NSCLC, and had to be chemo-naive, European Cooperative Oncology Group (ECOG) performance status 0-1. Prior immunotherapy with a PD1 or PDL1 inhibitor was allowed. Patients could be registered for PIN prior to (stage 1), or after (stage 2) initiation of induction chemotherapy. If any tumour shrinkage was observed (any shrinkage of RECIST target lesions), following a minimum of 3 cycles of platinum doublet chemotherapy, patients were randomised 1:1 using a centralised online system, to either olaparib (300 mg twice daily by mouth in 21-day cycles) or placebo, which was continued until disease progression, or unacceptable toxicity. Intention to treat (ITT) analyses of the primary endpoint included all randomised participants. Per protocol (PP) safety analysis included all participants who received at least one dose of study drug. Primary endpoint was progression-free survival (PFS), with a one-sided *p*-value of 0.2 to demonstrate statistical significance. Hazard ratios (HR) for PFS were both unadjusted and adjusted for the randomisation balancing factors (smoking status and histology). The trial was registered with ClinicalTrials.gov (NCT01788332) and EudraCT (2012-003383-51).

**Findings:**

A total of 940 patients were assessed for stage 1 eligibility of whom 263 were registered between Feb 24, 2014 and Nov 7, 2017. 194 patients were excluded prior to stage 2 (no tumour shrinkage or unevaluable) and 70 were randomised; 32 (46%) to Olaparib and 38 (54%) to placebo. 4% (3/70) of patients randomised had a CR and 96% (67/70) had a PR (or other evidence of tumour response/mixed stable) during induction therapy. A total of 36 patients were registered in stage 2 only, i.e., post induction therapy. Intention to treat (ITT) unadjusted analysis showed a PFS hazard ratio (HR) of 0.83 (one-sided 80% CI upper limit 1.03, one-sided unadjusted log rank test *p*-value=0.23). ITT Cox-adjusted model showed a HR 0.73 (one-sided 80% CI upper limit 0.91, one sided *p*-value 0.11). Adverse events were reported in 31/32 subjects (97%) in the olaparib arm and 38/38 (100%) in the placebo group. The most commonly reported adverse events in the olaparib group were fatigue (20/31; 65%), nausea (17/31; 55%), anaemia (15/31; 48%) and dyspnea (13/31; 42%). In the placebo group the most common adverse events were fatigue (25/38; 66%), coughing (22/38; 58%), dyspnea (15/38; 39%) and nausea (11/38; 29%). There were no treatment-related deaths.

**Interpretation:**

PFS was longer in the olaparib arm, but this did not reach statistical significance. When the PFS HR was adjusted for smoking status and histology, a significant difference at the one-sided 0.2 level was observed, suggesting that tumour control may be achieved for chemosensitive NSCLC treated with PARP monotherapy. We speculate that this signal may be driven by a molecular subgroup harbouring HRD.

**Funding:**

This study was funded between AstraZeneca CRUK, National Cancer Research Institute, and Cancer Research UK Feasibility Study Committee.

**Research in context**

**Evidence before this study**

We searched MEDLINE from Jan 1, 2009, to 1 Nov 2021 for clinical trials using the terms “non-small cell lung cancer”, “PARP”, “olaparib”, or “maintenance”, “phase II”, “randomised”, “placebo” without any language restrictions. This search revealed no evidence of any previously published placebo controlled, switch maintenance study.

**Added value of this study**

To our knowledge, PIN is the first placebo-controlled switch maintenance study of olaparib in patients relapsed non-small cell lung cancer. Olaparib was associated with both longer PFS and OS in the experimental arm, however, did not reach statistical significance in the intention to treat population, but in a planned analysis involving stratification for smoking status and histology, this difference was statistically significant.

**Implications of all the available evidence**

Some NSCLCs may harbour homologous recombination deficiency, accounting for the observed signal of efficacy for Olaparib. Chemotherapy is presently the front-line standard of care for advanced NSCLC and the combination of PARP inhibition, and anti-PDL1 immune checkpoint inhibition is being explored in a switch maintenance study design.

Alt-text: Unlabelled box

## Introduction

The repair of double stranded DNA breaks is essential for viability of cancer cells. Cancers harbouring homologous recombination deficiency (HRD), required for efficient repair of double strand breaks, results in a switch to an error-prone DNA repair pathway exposing a therapeutically exploitable vulnerability to PARP inhibition.^1^ Trapping of PARP on the DNA by a small molecule PARP inhibitor (PARPi) creates DNA-PARP complexes which create a physical block to DNA repair, leading to replication fork collapse and catastrophic DNA double strand breaks which are selectively lethal to the cancer cell. This paradigm is called *synthetic lethality*. PARP inhibition is now standard therapy in ovarian and breast cancers harbouring HRD due to BRCA1 and BRCA2 mutations,^1^, ^2^, ^3^, ^4^, ^5^ and recently HRD has been shown to be exploitable in other solid tumours such as prostate^6^ and pancreatic cancer.^7^

BRCAness can be phenocopied by the loss of components other than BRCA1 or 2 in the HR pathway.^8^ Pan-cancer analysis has revealed widespread bi-allelic inactivation of HR pathway genes involving more than 5% of all cancers including non-small cell lung cancer (NSCLC).^9^ Homologous recombination deficiency (HRD) determined by whole exome sequencing has been reported in 18.7% of patients with advanced NSCLC,^10^ with Foundation One HRD LOH/HRD score in another study being common, occurring in 66% of patients.^11^ Furthermore, the presence of HRD in NSCLC has been associated with sensitivity to PARP inhibition in preclinical models^12^ with evidence of impaired RAD51 foci formation and platinum sensitivity^13^

HRD is associated with increased platinum sensitivity which can be utilised as a surrogate biomarker and has been repeatedly shown to be predictive for PARP inhibitor sensitivity in ovarian cancer.^2^^,^^14^^,^^15^ We therefore hypothesised that response of NSCLCs to platinum-based chemotherapy might enrich for NSCLCs harbouring HRD and therefore exhibit sensitivity to maintenance PARP inhibition. In this phase 2 trial (PIN - PARP Inhibitor in advanced NSCLC) we address the question of whether switch maintenance therapy with olaparib in chemosensitive advanced NSCLC leads to increased efficacy.

## Methods

### Study design

A multicentre, randomised, double-blind, placebo-controlled, parallel arm, phase 2 trial of Olaparib Maintenance versus Placebo Monotherapy in Patients with Advanced Non-Small Cell Lung Cancer (PIN) was conducted at 23 investigative hospital sites in the UK. The trial protocol can be found in the supplementary materials. The study was approved by the National ethics committee (reference 13/WA/0117**)**, and The University Hospitals of Leicester NHS Trust (lead centre) was the first centre to obtain ethical approval. All participating hospitals obtained ethical approval.

### Patients

Patients were eligible for registration into the PIN trial prior to initiation of chemotherapy (stage 1 registration, with consent to permit research blood and tissue sample collection during induction therapy). If patients had had completed induction chemotherapy with evidence of any response to treatment patients could consent for randomisation to Olaparib or placebo (stage 2 registration) after a minimum of three cycles of induction chemotherapy. The protocol originally required all patients to have PR/CR and be registered prior to induction chemotherapy. To improve recruitment, we changed the eligibility criteria to allow patients to be registered after induction radiotherapy and to accept patients with <30% tumour shrinkage. Patients had to provide written informed consent, be 18 years of age or older, with a histological diagnosis of stage IIIB/IV NSCLC (either squamous or non-squamous) as defined by the American Joint committee on Cancer staging criteria (7th edition) for lung cancer, not amenable to curative therapy and with no prior treatment with systemic chemotherapy for advanced NSCLC. Patients harbouring EGFR mutation positive or ALK translocation positive NSCLC were eligible following prior treatment with a tyrosine kinase inhibitor. Patients who had previously received a PD1 checkpoint inhibitor were deemed eligible. ECOG performance status had to be 0-1 with adequate renal, hepatic, or haematological parameters. Prior exposure to PARP inhibitors was not allowed.

Stage 1 registered participants received of platinum-based doublet induction therapy and those who were found to have any evidence of radiological response (including mixed stable/response or evidence of tumour shrinkage that did not reach the criteria of partial response according to RECIST) were then eligible to be randomised. We also allowed participants who had already completed platinum-based doublet induction therapy to enter the trial at randomisation if they were eligible for both stage 2 registration and randomisation criteria.

### Randomisation and masking

If consenting participants had evidence of radiological response during their induction chemotherapy (complete or partial response or stable disease by RECIST1.1 with evidence of tumour shrinkage), the research nurse randomly assigned in a 1:1 ratio to olaparib or placebo using a central, interactive web response system. The olaparib and placebo tablets and packaging were identical with each bottle having a unique kit number for dispensing. Randomisation was stratified using permuted blocks (block size 4) according to histology (squamous vs non-squamous) and smoking status (never smoked versus ever smoked). Patients, their clinicians, and the research nurses were blinded to treatment allocation. A central unblinding process was available should treating clinicians need to know treatment allocation to ensure patient safety.

### Procedures

The initial study dose of oral olaparib or placebo tablets was 300mg administered twice daily in 21-day cycles until disease progression, unacceptable toxicity, or patient withdrawal of consent. Dose interruption was allowed for any grade of toxicity for a maximum of 14 days until complete recovery or reversion to Common Terminology Criteria for Adverse Events (CTCAE) version 4.03 grade 1. Dose reductions were allowed to 250mg twice daily (dose level -1) or 200 mg twice daily (dose level -2). Dose escalations were not permitted.

### Outcome measures

Participants were monitored by CT scan every two cycles until disease progression. The primary endpoint was progression-free survival (PFS), assessed according to Response Evaluation Criteria in Solid Tumors (RECIST), version 1.1. PFS was defined as the time from randomisation until progression or death from any cause. Participants still alive and progression-free were censored at the time of their last evaluable CT scan.

Secondary endpoints included objective response rate (ORR), toxicity rate, overall survival and change in tumour volume. ORR was assessed at the end of cycle 2 according to RECIST version 1.1 and change in tumour volume was calculated as the change in sum of longest diameters of target tumours from randomisation to the 6-week CT scan. After the discontinuation or completion of the trial regimen, patients were followed for survival. Overall survival (OS) was defined as the time from randomisation until death from any cause. Participants still alive were censored at the time last seen. Toxicity rate was reported as any adverse events reported during treatment affecting more than 10% of participants, summarised by CTCAE version 4.03 grade.

### Statistical analysis

The primary objective of the PIN trial was to establish whether maintenance olaparib in patients with DNA damage sensitive NSCLC, as measured by PFS, has sufficient anti-tumour activity to warrant further investigation with a phase 3 trial. For this purpose a one-sided *p*-value of 0.2 was considered suitable, while conventional two-tailed tests at the 0.05 level and 95% confidence intervals are used elsewhere. Assuming a true hazard ratio of 0.65 for olaparib versus placebo, 68 participants were required in total to demonstrate statistical significance between the arms, based on the log-rank test, 80% power and a one-sided α (type I error) of 0.2 and recruitment period of 18 months (m). Minimum participant follow-up was at least 6m, or until disease progression, complete withdrawal, or death. The primary analysis of data required 63 PFS events.

The analysis of efficacy endpoints was performed for both the intention-to-treat (ITT) population and per-protocol (PP) population with the toxicity endpoint in only the safety population who received trial treatment. Patients registered at both stage 1 and 2 were combined and not compared. The ITT population consisted of all randomised subjects. The PP population excluded all participants who: i) did not receive at least one cycle of olaparib or placebo; ii) were found to be ineligible; iii) were RECIST unevaluable and/or iv) had other protocol deviations likely to have an effect on the estimation of the efficacy endpoints.

For the primary endpoint of PFS, the effect of olaparib compared with placebo was estimated by the unadjusted hazard ratio (HR) with an 80% one-sided confidence interval (CI) (upper limit) and p-value was calculated using a 1-sided logrank test. The median PFS with 95% CI and Kaplan–Meier curves were calculated for each arm of the trial. If the hazards were found to be proportional, a Cox regression was performed to adjust the hazard ratio for i) the stratification factors (smoking status and histology) or ii) stratification factors, and any unbalanced balance characteristics. OS was a secondary endpoint, and calculated as the median, HR (with 95% CI and 2-sided logrank test p-values, with Kaplan–Meier curves for time to OS endpoint. No patients were missing follow-up data.

An Independent Data Monitoring Committee (IDMC) reviewed accumulating data at regular intervals, but there were no formal stopping guidelines.

CONSORT reporting guidelines were followed in writing this report. The Statistical Analysis Plan can be found in the supplementary materials. As an exploratory analysis, a post-hoc analysis of squamous and non-squamous OS was also performed.

The trial was registered with ClinicalTrials.gov (NCT01788332) and EudraCT (2012-003383-51).

### Role of the funding source

The funder of the study had no role in study design, data collection, data analysis, data interpretation, or writing of the manuscript. DF, AC, CP had access to the dataset and had final responsibility for the decision to submit for publication.

## Results

**Figure 1:**
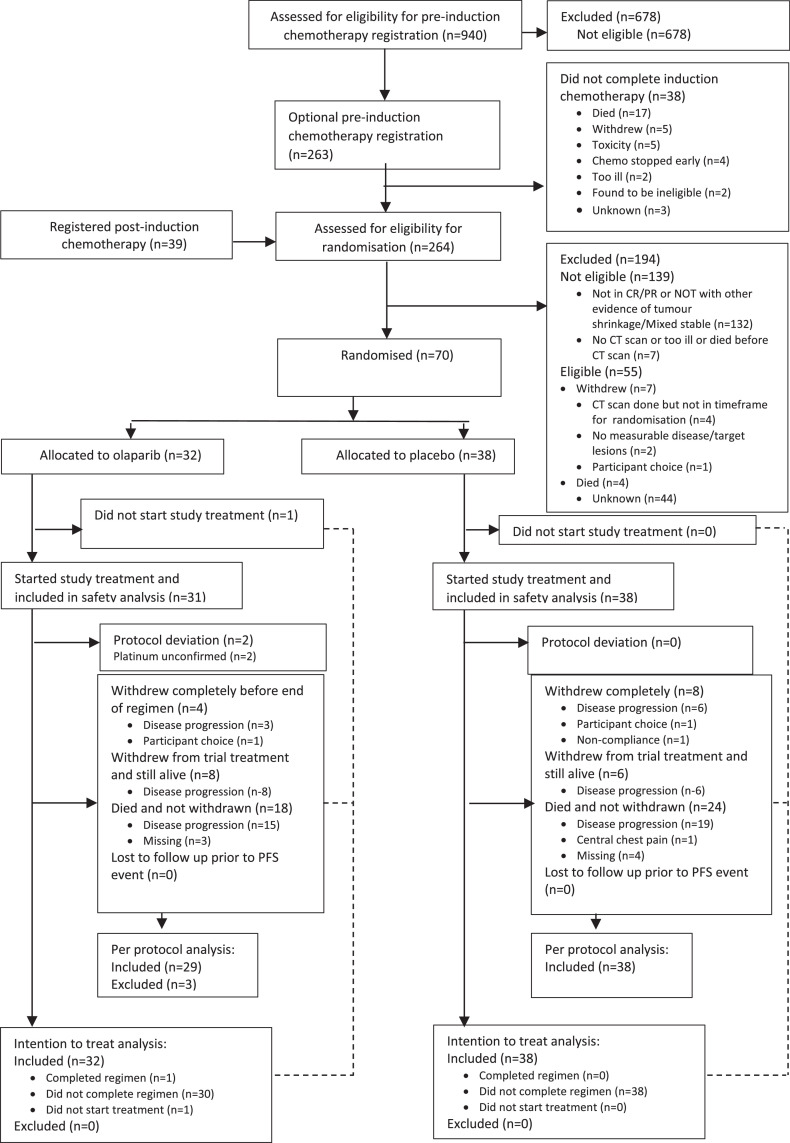
PIN trial - Consort diagram.

A total of 940 patients were assessed for stage 1 eligibility of whom 263 were registered between 24^th^ February 2014 and 7^th^ November 2017 (CONSORT diagram - Figure 1). Of the stage 1 registered patients who commenced chemotherapy, 38 did not complete treatment. Another 39 patients were registered at stage 2 having completed their chemotherapy. In total 264 patients were assessed for eligibility for randomisation of whom 70 were randomised to olaparib (32) or placebo (38) between 27^th^ August 2014 and 14^th^ November 2017 (ITT population). The trial was completed when all patients had completed their minimum follow-up and 63 events were confirmed.

Patient characteristics and radiological response to induction chemotherapy of the ITT population are summarised in Table 1. The ITT population was highly enriched for radiological response to induction chemotherapy with 4% (3/70) having complete radiological response, 89% (62/70) having partial response, and 7% (5/70) having some evidence of tumour shrinkage not meeting the RECIST version 1.1 criteria for partial response. The median number of chemotherapy induction cycles was 4 (inter-quartile range (IQR) 4-4). The majority of patients had a diagnosis of the NSq subtype (41/70; 59%), stage IV NSCLC (47/70; 67%) (with metastases to brain in 3% (2/70), bone in 16% (11/70), adrenal gland in 7% (5/70)), and ECOG performance status 1 (48/70; 69%). Carboplatin-based doublet chemotherapy was received by the majority of patients (45/70; 64%).

**Table 1:** Clinical and demographic characteristics and response to induction chemotherapy in olaparib versus placebo arms.

	Olaparib	Placebo
	*N*=32	*N*=38
**Recruitment time point - *N* (%)**		
Pre induction chemotherapy	14 (44)	20 (53)
Post induction chemotherapy	18 (56.3)	18 (47.4)
**Sex - *N* (%)**		
Male	16 (50)	24 (63)
Female	16 (50)	14 (37)
**Age - median (IQR)**	65 (61–72)	63 (59–70)
**ECOG status - *N* (%)**		
0	9 (28)	13 (34)
1	23 (72)	25 (66)
**Smoking history - *N* (%)**		
Never smoked	3 (9)	3 (8)
Ever smoked	29 (91)	35 (92)
**Non-small cell lung cancer type - *N* (%)**		
Adenocarcinoma	19 (59)	18 (47)
Squamous	13 (41)	18 (47)
Large cell nos	0(0)	1 (3)
Mixed adenocarcinoma/Squamous	0(0)	1 (3)
**Type of induction chemotherapy treatment - *N* (%)**		
Gemcitabine	10 (31)	14 (37)
Cisplatin	25 (78)	34 (90)
Carboplatin	23 (72)	31 (82)
Pemetrexed	17 (53)	17 (45)
Paclitaxel	1 (3)	0(0)
Vinorelbine	6 (19)	4 (11)
Docetaxel	0(0)	1 (3)
**Number of cycles of induction chemotherapy - median (IQR)**	4 (4-4)	4 (4-4)
**Response to induction chemotherapy - *N* (%)**		
Complete response	2 (6)	1 (2.6)
Partial response	28 (86)	34 (89)
Other evidence of tumour shrinkage/Mixed stable	2 (6)	3 (8)

## Efficacy

**Figure 2:**
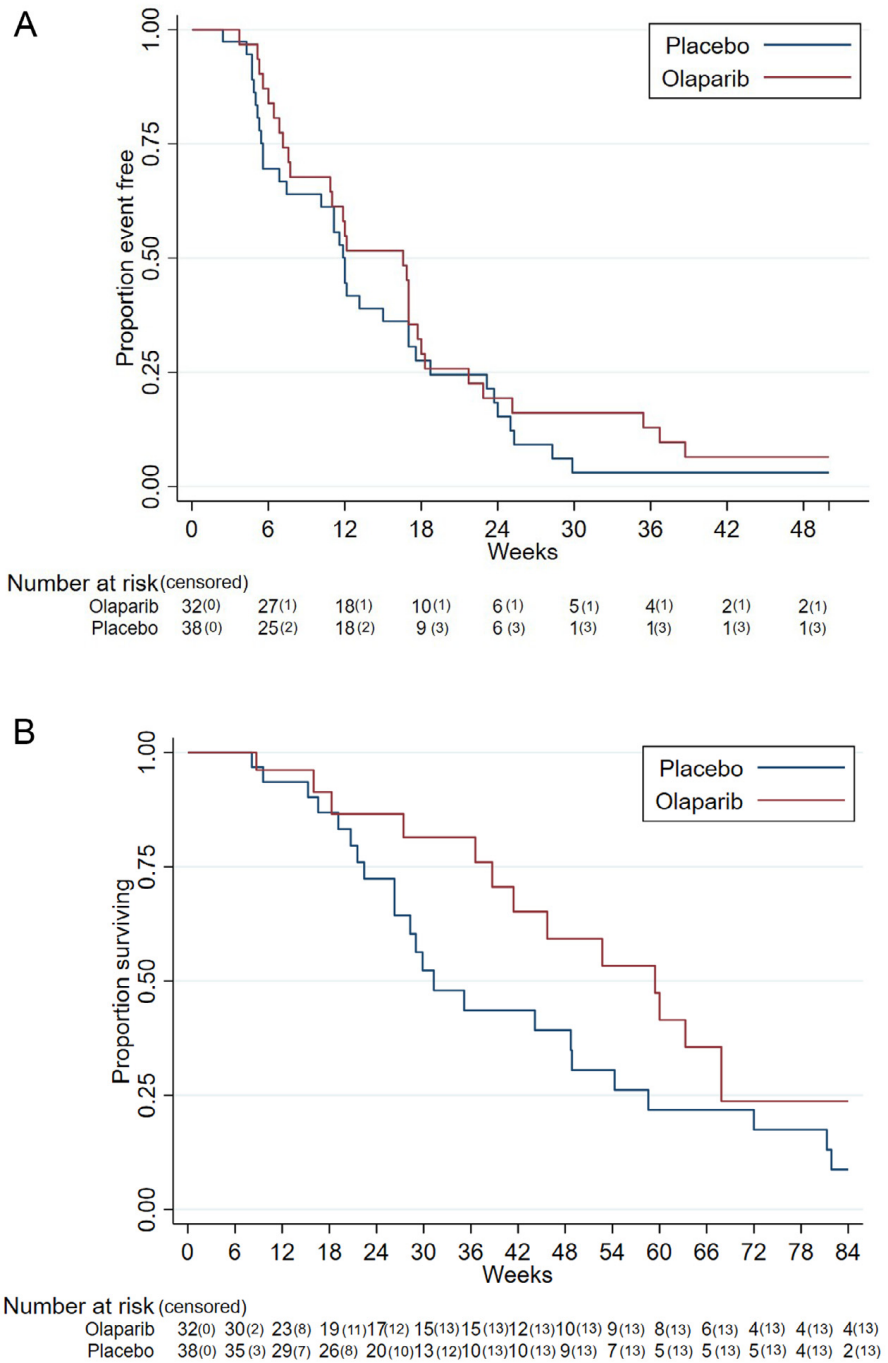
Progression-free and overall survival by trial arm. A. Progression Free Survival (ITT unadjusted). HR 0.83 (one-sided 80% CI upper limit 1.03, one-sided *p* value 0.23). B. Overall survival (ITT population), HR 0.68 (95% CI 0.37–1.26, two-sided *p*-value 0.22).

In the ITT unadjusted analysis, PFS hazard ratio (HR) (primary endpoint) was 0.83 (one sided 80% Confidence Interval [CI] upper limit 1.03, unadjusted one-sided log rank test *p*-value=0.23 Figure 2A). The ITT Cox-adjusted model, adjusted for smoking history and histology, showed PFS HR was 0.73 (one sided 80% CI upper limit 0.91, one sided *p*-value 0.11) (Table 2). There was no evidence of non-proportional hazards for the above analyses (supplementary materials)."?>

**Table 2:** Efficacy of olaparib versus placebo with respect to progression-free survival (ITT, PP and Cox adjusted populations) and overall survival.

	Olaparib (*n*=32)	Placebo (*n*=38)	HR^d^	*p*-value^c^
**Progression-free survival events - *N* (%)**	31 (97)	35 (92)		
**Progression-free survival (weeks) -median (IQR)**				
**ITT population**	16.6 (7.1–21.7)	12 (5.6–18.7)	0.83 (–1.02)	0.23
**PP population**	16.6 (7.6–18.3)	12.0 (5.6–18.7)	0.86 (1.06)	0.28
**Cox model adjusted**^a^	16.6 (7.1–21.7)	12 (5.6–18.7)	0.73 –0.91)	0.11
**Cox model adjusted**^b^	16.6 (7.1–21.7)	12 (5.6–18.7)	0.79 (1.02)	0.22
**Overall survival (weeks) - median (IQR)**				
**ITT population**	59.4 (38.7–67.9)	31.3 (22.4–58.6)	0.68 (0.37–1.26)	0.22

a Adjusted for stratification factors (smoking and histology).

b Adjusted for stratification factors, T-stage, and N-stage.

c One-sided p-values for progression-free survival (PFS). Two-sided p-value for overall survival (OS).

d PFS hazard ratio (HR) 80% one-sided confidence interval (CI) upper limit and for OS HR two-sided 95% CI.

For OS (secondary endpoint), in the ITT population HR was 0.68 (95% CI 0.37–1.26: two-sided *p*-value 0.22), (Figure 2B). ORR was 2%. One patient receiving olaparib exhibited a complete response in the target lesion at cycle 4 and one patient had a partial response of the target lesion at cycle 6. Median change in tumour volume from randomisation to end of cycle 2 was 0cm (IQR -1 to 9) for olaparib and 4cm (IQR -1 to 10) for placebo.

Median duration of follow-up in censored patients in the ITT population could not be calculated as 67/70 (96%) patients progressed or died. Times on study for three patients that were still alive and not progressed at complete withdrawal were 0.67 m (two patients) and 0.47 m (one patient). Additionally, one patient missed two visits prior to death with no evidence for disease progression by RECIST and was censored at date of last RECIST (4 m).

In an unplanned post-hoc analysis of the Sq subgroup, median OS was 14m for olaparib (95% CI 9 -not reached; *n*=13) and for placebo 7 m (95% CI 5–11; *n*=18) In the NSq group median OS was 12m for olaparib (95% CI 4–16; *n*=19) and for placebo 10m (95% CI 6–19; *n*=20) (supplementary materials).

## Toxicity

31 participants started olaparib treatment and 38 started placebo. Olaparib was generally well tolerated. Dose reductions were reported in 4/31 (13%) subjects in the olaparib arm (all four reduced to 250 mg) and 4/38 (11%) in the placebo group (two reduced to 250 mg and two further reduced to 200 mg). The incidence of severe adverse events was similar between treatment groups: SAEs occurred in 9/31 (29%) of subjects in the olaparib group and 10/38 (26%) in the placebo group. Most SAEs occurred once in one subject. Only lung infection and dyspnea were reported in more than one subject. These were reported in two subjects each in the placebo group. Adverse events were reported in 31/31 subjects (100%) in the olaparib arm and 38/38 (100%) in the placebo group (PP population). The most commonly reported treatment-related AEs (TRAEs; occurring in more than 10% of subjects) in the olaparib group were anaemia (15/31; 48%), neutropenia (4/31; 13%), thrombocytopenia (4/31; 13%), constipation (4/31; 13%), diarrhoea (4/31; 13%), nausea (17/31; 55%), vomiting (7/31; 23%), fatigue (20/31; 65%), upper respiratory infection (4/31; 13%), anorexia (11/31; 35%), back pain (5/31; 16%), dizziness (6/31; 19%), headache (6/31; 19%), coughing (11/31; 35%), dyspnea (13/31; 42%) and skin rash (4/31; 13%). Diarrhoea (8/38; 21%), back pain (8/38; 21%) and coughing (22/38; 58%) were more common in the placebo group, as was the incidence of dry mouth (5/38; 13%), dyspepsia (7/38; 18%), flatulence (5/38; 13%), insomnia (6/38; 16%), and hypertension (9/38; 24%). The incidence of fatigue, oedema peripheral, upper respiratory infection, anorexia, and dyspnoea was similar between treatment groups (Table 3). A breakdown by CTCAE grade is shown in the supplementary materials.

**Table 3:** Adverse events of any grade occurring in 10% or more of patients (safety population) in olaparib vs placebo arms.

	Subjects - *N* (%)		Occurrences - *N* (%)	
	Olaparib	Placebo	Olaparib	Placebo
	*N*=31	*N*=38	*N*=31	*N*=38
**Blood and lymphatic system**				
Anaemia	15 (48)	10 (26)	45	26
Neutropenia	4 (13)	0(0)	6	0
Thrombocytopenia	4 (13)	1 (3)	12	3
**Gastrointestinal disorders**				
Constipation	4 (13)	4 (11)	9	7
Diarrhoea	4 (13)	8 (21)	12	12
Dry mouth	1 (3)	5 (13)	1	5
Dyspepsia	3 (10)	7 (18)	4	13
Flatulence	1 (3)	5 (13)	1	3
Nausea	17 (55)	11 (29)	44	21
Vomiting	7 (23)	4 (11)	17	5
**General disorders and administration site conditions**				
Fatigue	20 (65)	25 (66)	50	66
Oedema peripheral	3 (10)	4 (11)	8	6
**Infections and infestations**				
Upper respiratory infection	4 (13)	5 (13)	7	9
**Metabolism and nutrition disorders**				
Anorexia	11 (35)	12 (32)	22	21
**Musculoskeletal and connective tissue disorders**				
Back pain	5 (16)	8 (21)	6	10
**Nervous System Disorders**				
Dizziness	6 (19)	3 (8)	6	3
Headache	6 (19)	6 (16)	8	6
**Psychiatric disorders**				
Insomnia	2 (6)	6 16)	9	8
**Respiratory, thoracic, and mediastinal disorders**				
Coughing	11 (35)	22 (58)	33	47
Dyspnoea	13 (42)	15 (39)	28	35
**Skin and subcutaneous tissue disorders**				
Rash	4 (13)	3 (8)	10	3
**Vascular disorders**				
Hypertension	3 (10)	9 (24)	15	18

In both treatment groups, most AEs were Grade 1 or 2. There were no grade 4+ TRAEs. SAEs resulting in death were reported in 1 (3%) subject in the olaparib group and no subjects in the placebo group. The SAE resulting in death was not related to olaparib. Nausea was the only study drug-related SAE in the olaparib group, reported in one subject. There were no study drug-related SAEs in the placebo group. No subjects had treatment emergent adverse events (TEAEs) leading to death.

## Discussion

The PIN trial tested the hypothesis that a subgroup of patients exhibiting response to platinum-based doublet chemotherapy might be enriched for HRD and benefit from a PARP inhibitor. This study was conducted in a placebo controlled double blind design to minimise the risk of bias. When the PFS HR was adjusted for smoking status and histology, a significant difference at the one-sided 0.2 level was observed, suggesting that tumour control may be achieved for chemosensitive NSCLC treated with PARP monotherapy. We speculate that this signal may be driven by a molecular subgroup harbouring HRD.

PARP inhibition has been explored in combination with paclitaxel and carboplatin in NSCLC but did not meet primary endpoint in a double-blind placebo-controlled phase III trial.^16^

In PIN a personalised strategy was employed based on phenotype, to enrich for patients with tumours more likely to respond to a PARP inhibitor, i.e., Radiological response to platinum-based chemotherapy. By far the majority of patients enrolled into PIN had either complete or partial response to first line platinum-based chemotherapy, satisfying the requirement for chemosensitive tumour enrichment. PARP inhibitors have established a role in the standard of care for ovarian cancer.^2^^,^^17^, ^18^, ^19^ Use of response to induction platinum-based therapy has consistently been used as a surrogate to enrich for PARP-sensitive ovarian cancer subpopulations irrespective of BRCA1 and BRCA2 status.^14^^,^^15^

In PIN, induction chemotherapy was heterogeneous, ie. any combination of chemotherapy was allowed prior to randomisation on the basis that the combination contained either cisplatin or carboplatin. This is in common with randomised studies for example in ovarian cancer,^20^ whereby response to platinum-based doublet induction therapy was shown to predict response to PARP inhibition. We expected patients with response to platinum-based doublet to be enriched for HRD. Patients who received cisplatin would have been eligible to receive pemetrexed maintenance,^21^ however only a minority of patients in PIN received cisplatin. Although patients with mutated oncogenic drivers such as EGFR and ALK were eligible following relapse after kinase inhibitors, such patients were not enrolled.

Recent pan-cancer studies have provided genomic evidence of mutation signatures associated with HRD in NSCLC.^22^ The genomic landscape of NSCLC exhibits low frequency deleterious mutations which impair HR, such as copy number alterations or mutations including RAD51 (1.3%), ATM (3%), BRCA2 (1.3%)^23^ that are enriched in platinum responsive cancers. In the PIN trial, baseline archival tumour tissues and plasma were collected on all baseline and 50% of progressing patients. Translational studies are planned to determine if enrichment of HRD associated mutations was found in the ITT population.

Since the development of the PIN trial, the standard of care has changed with addition of an anti-PD1 checkpoint inhibitor in the first line treatment setting.^24^, ^25^, ^26^ PARP inhibitors promote cytosolic DNA fragments which, when sensed by cGAS (cyclic guanosine monophosphate adenosine monophosphate synthase), lead to activation of the stimulator of interferon genes (STING) pathway, and a Th1 inflammatory response.^27^^,^^28^ The combination of anti-PD1 immunotherapy with PARP inhibitor has been explored in NSCLC with suggested synergy^29^^,^^30^ The rationale for combining a PARP inhibitor and PD1 or PDL1 immune checkpoint inhibitor has led to the development of the ORION randomised phase II trial (NCT03775486), which is evaluating checkpoint inhibition with the anti-PDL1 antibody durvalumab with or without olaparib as maintenance therapy in NSCLC. This study has completed accrual and presents a logical progression, building on the PARP inhibitor monotherapy data in the PIN trial. An unplanned post-hoc analysis, suggested Sq subtypes had a longer median survival time in the olaparib group compared to placebo, but our subgroup size was too small to draw conclusions other than it would be interesting to investigate this in a larger trial.

In summary, although the PIN trial did not meet its PFS primary endpoint, in a planned adjusted analysis, there was evidence of longer PFS in the Olaparib group, suggesting that a larger trial would be required to establish if a significant treatment effect exists. Our results provide some supporting evidence that patient enrichment based on response to platinum-based chemotherapy, may select an HRD+ subgroup likely to benefit from PARP inhibitor monotherapy. DNA damage response gene correlation with clinical response might enable patient stratification to improve PARP inhibitor efficacy in a future study.

## Contributors

DAF wrote manuscript, patient enrolment, data collection and data interpretation, LN performed Conception, study design, acquisition and accuracy of data and the co-ordination, AC drafted manuscript, accessed and verified the underlying data, data analysis, CP accessed and verified the underlying data, did data analysis, wrote manuscript, Tables and Figures, GGr wrote the manuscript, FB performed patient enrolment and data collection, JL did patient enrolment and data collection, GGa responsible for acquisition and accuracy of data and the co-ordination of the trial, AW monitored acquisition and accuracy of data and the co-ordination of the trial, MN performed patient enrolment, data collection, SD did patient enrolment, data collection.

All authors contributed to the management and delivery of the trial and review and revision of the manuscript. AC and GGr contributed equally.

## Data sharing statement

Trial data relating to this publication shall remain confidential to the sponsor organisation and will not be disclosed, except where disclosure might be required in accordance with pharmacovigilance duties of the parties involved. Individual participant data can be made available, after deidentification, to investigators who provide a written request in accordance with General Data Protection Regulation and following authorisation from the sponsor organisation, starting immediately and ending 3 years after publication. Data sharing requests should be directed to DAF and A.C.

The Centre for Trials Research (Cardiff) is committed to the responsible sharing of clinical trial data and trial samples with the wider research community. Data access is administered through the Centre for Trials Research Data Release Committee. Requests for data access and sharing trials should be completed onto the request for data form and emailed to the Chief Investigator (DAF).

## Declaration of interests

AC reports grants from Cancer Research UK, during the conduct of the study. FB reports grants and personal fees from Astra Zeneca, outside the submitted work. GGr reports grants from AstraZeneca, personal fees from AstraZeneca, outside the submitted work. DAF reports grants from Astex Therapeutics, personal fees from Aldeyra, grants from Boehringer Ingelheim, non-financial support from Clovis, non-financial support from Eli Lilly, from BMS, personal fees from Inventiva, personal fees from Paredox, personal fees and non-financial support from Roche, grants from MSD, grants from Bayer, during the conduct of the study. JL reports personal fees from Astra Zeneca, personal fees from Boehringer Ingelheim, personal fees from Roche, outside the submitted work. MN reports payments for Pfizer Lung Cancer Advocacy board work; membership of the BMS lung cancer screening committee; and participation on a data safety monitoring board for Roche. All other authors declare no competing interests.

## Appendix Supplementary materials

Image, application 1
