# Supplementary material for: Olaparib maintenance versus placebo monotherapy in patients with advanced non-small cell lung cancer (PIN): A multicentre, randomised, controlled, phase 2 trial
Source: eClinicalMedicine. 2022 Aug 11;52:101595. doi: 10.1016/j.eclinm.2022.101595 (PMC9386392; doi:10.1016/j.eclinm.2022.101595)
Supplement: Supplementary file 1 [file mmc1.pdf]

Olaparib Maintenance versus Placebo Monotherapy in Patients with Advanced Non-Small Cell Lung Cancer (PIN): a Multicentre, Randomised, Controlled, Phase 2 Trial

Dean A. Fennell FRCP, Catharine Porter PhD, Jason Lester FRCR, Sarah Danson FRCP, Fiona Blackhall FRCP, Marianne Nicolson FRCP, Lisette Nixon PhD, Georgina Gardner BSc, Ann White, Gareth Griffiths PhD, Angela Casbard MSc

Supplementary materials

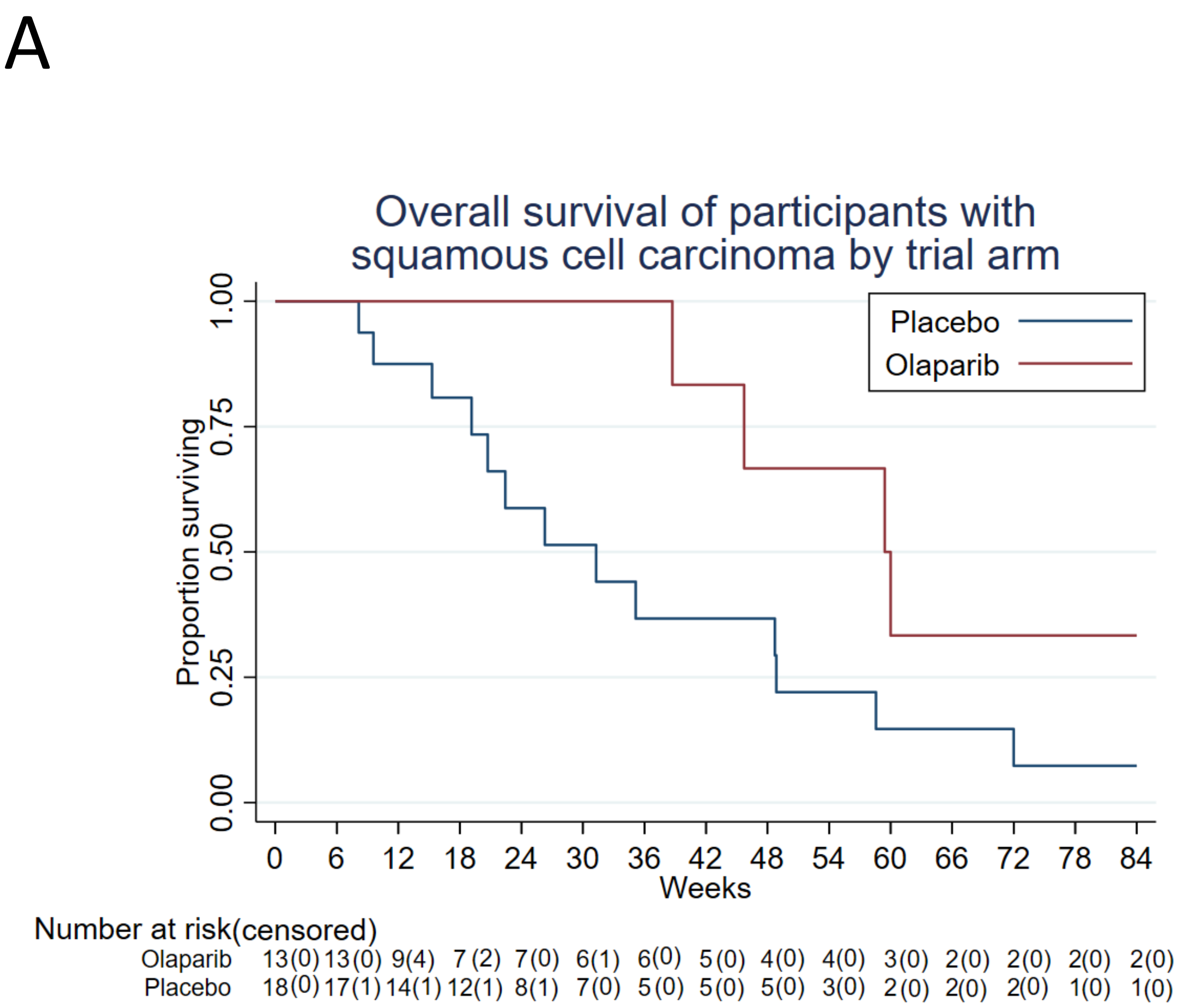
A
Overall survival of participants with squamous cell carcinoma by trial arm
Proportion surviving
0.00 0.25 0.50 0.75 1.00
Placebo
Olaparib
0 6 12 18 24 30 36 42 48 54 60 66 72 78 84
Weeks
Number at risk(censored)
Olaparib 13(0) 13(0) 9(4) 7(2) 7(0) 6(1) 6(0) 5(0) 4(0) 4(0) 3(0) 2(0) 2(0) 2(0) 2(0)
Placebo 18(0) 17(1) 14(1) 12(1) 8(1) 7(0) 5(0) 5(0) 5(0) 3(0) 2(0) 2(0) 2(0) 1(0) 1(0)

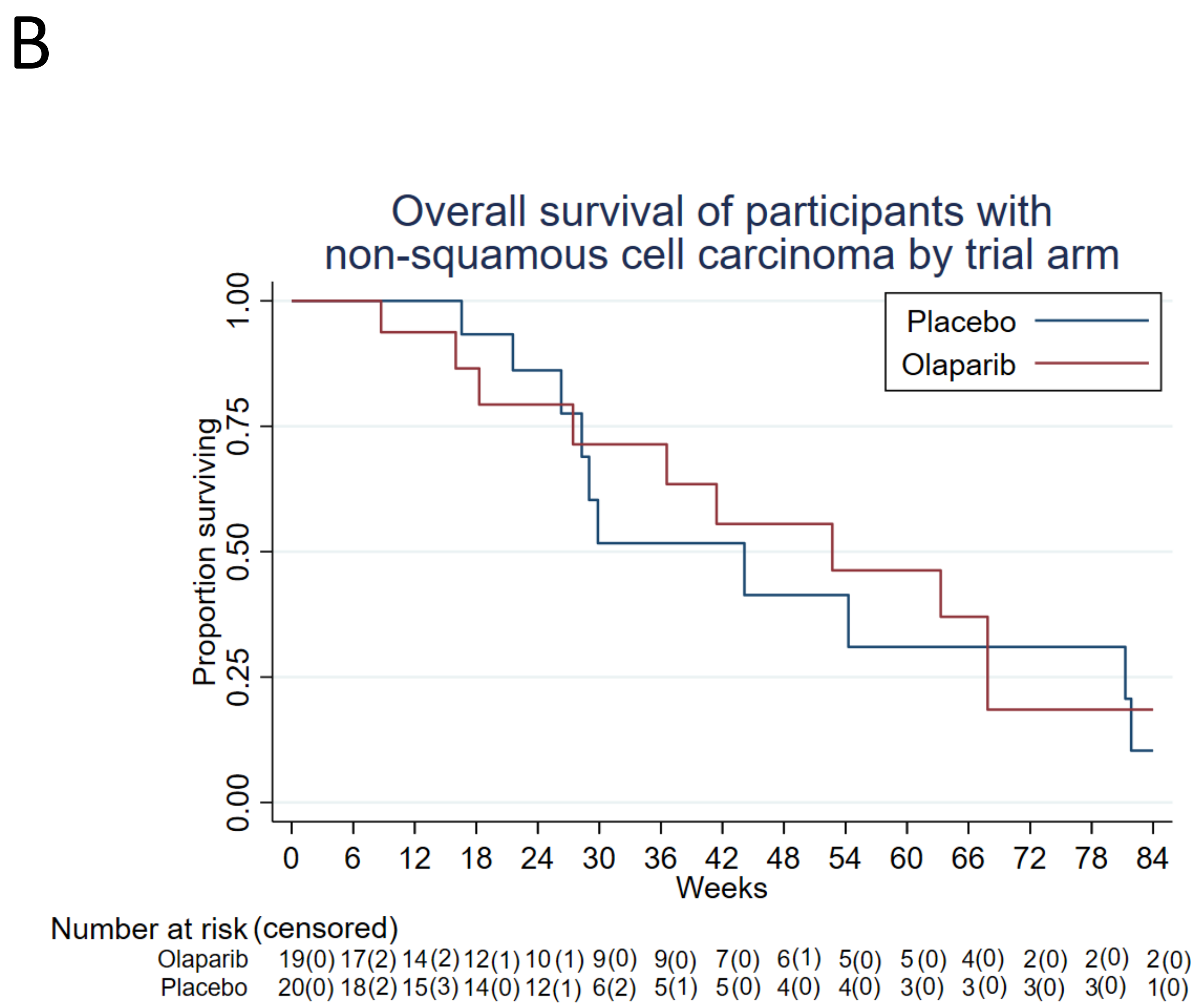
B
Overall survival of participants with non-squamous cell carcinoma by trial arm
Proportion surviving
0.00 0.25 0.50 0.75 1.00
Placebo
Olaparib
0 6 12 18 24 30 36 42 48 54 60 66 72 78 84
Weeks
Number at risk (censored)
Olaparib 19(0) 17(2) 14(2) 12(1) 10(1) 9(0) 9(0) 7(0) 6(1) 5(0) 5(0) 4(0) 2(0) 2(0) 2(0)
Placebo 20(0) 18(2) 15(3) 14(0) 12(1) 6(2) 5(1) 5(0) 4(0) 4(0) 3(0) 3(0) 3(0) 3(0) 1(0)

Supplementary tables

Table S1: Schoenfield Tests of proportionality of hazards

Model	p-value
PFS	
Unadjusted	0.41
Adjusted for smoking history and histology	0.67
OS	
Unadjusted	0.18

Table S2: Adverse events occurring in 10% or more of patients (safety population) in olaparib vs placebo arms by CTCAE grade (Version 4.03)

	**Subjects - N(%)**							
	**Olaparib (N=31)**				**Placebo (N=38)**			
**CTCAE (v 4.03) grade**	**1**	**2**	**3**	**4**	**1**	**2**	**3**	**4**
**Blood and lymphatic system**								
Anaemia	10 (32)	5 (16)	0(0)	0(0)	9 (24)	1 (3)	0(0)	0(0)
Neutropenia	3 (10)	1 (3)	0(0)	0(0)	0(0)	0(0)	0(0)	0(0)
Thrombocytopenia	3 (10)	1 (3)	0(0)	0(0)	1 (3)	0(0)	0(0)	0(0)
**Gastrointestinal disorders**								
Constipation	3 (10)	1 (3)	0(0)	0(0)	3 (8)	1 (3)	0(0)	0(0)
Diarrhoea	4 (13)	0(0)	0(0)	0(0)	6 (16)	0(0)	2 (5)	0(0)
Dry mouth	1 (3)	0(0)	0(0)	0(0)	4 (11)	1 (3)	0(0)	0(0)
Dyspepsia	3 (10)	0(0)	0(0)	0(0)	7 (18)	0(0)	0(0)	0(0)
Flatulence	1 (3)	0(0)	0(0)	0(0)	5 (13)	0(0)	0(0)	0(0)
Nausea	13 (42)	4 (13)	0(0)	0(0)	7 (18)	4 (11)	0(0)	0(0)
Vomiting	4 (13)	3 (10)	0(0)	0(0)	3 (8)	1 (3)	0(0)	0(0)
**General disorders and administration site conditions**								
Fatigue	16 (52)	4 (13)	0(0)	0(0)	18 (47)	7 (18)	0(0)	0(0)
Oedema peripheral	3 (10)	0(0)	0(0)	0(0)	4 (11)	0(0)	0(0)	0(0)
**Infections and infestations**								
Upper respiratory infection	2 (6)	2 (6)	0(0)	0(0)	2 (5)	3 (8)	0(0)	0(0)
**Metabolism and nutrition disorders**								
Anorexia	8 (26)	3 (10)	0(0)	0(0)	7 (18)	5 (13)	0(0)	0(0)
**Musculoskeletal and connective tissue disorders**								
Back pain	2 (6)	1 (3)	2 (6)	0(0)	6 (16)	2 (5)	0(0)	0(0)
**Nervous System Disorders**								
Dizziness	4 (13)	2 (6)	0(0)	0(0)	3 (8)	0(0)	0(0)	0(0)
Headache	4 (13)	1 (3)	1 (3)	0(0)	6 (16)	2 (5)	0(0)	0(0)
**Psychiatric disorders**								
Insomnia	2 (6)	0(0)	0(0)	0(0)	5 (13)	1 (3)	0(0)	0(0)
**Respiratory, thoracic and mediastinal disorders**								
Coughing	11 (35)	0(0)	0(0)	0(0)	16 (42)	5 (13)	1 (3)	0(0)
Dyspnoea	10 (32)	2 (6)	1 (3)	0(0)	10 (26)	4 (11)	1 (3)	0(0)

**Skin and subcutaneous tissue disorders**								
Rash	4 (13)	0(0)	0(0)	0(0)	2 (5)	1 (3)	0(0)	0(0)
**Vascular disorders**								
Hypertension	1 (3)	1 (3)	1 (3)	0(0)	3 (8)	5 (13)	1 (3)	0(0)

Legend: Abbreviations: Common Terminology Criteria for Adverse Events (CTCAE)

PIN

PARP Inhibitor in advanced NSCLC

CLINICAL TRIAL PROTOCOL

Short Title: Parp inhibitor in advanced NSCLC

Full Title: A randomised phase II trial of Olaparib maintenance versus placebo monotherapy in patients with chemosensitive advanced non-small cell lung cancer

Version: 9.0

Date: 11th July 2017

EudraCT No: 2012-003383-51

ISRCTN No: 42518913

Funder: AstraZeneca (an NCRN/AstraZeneca collaborative NCRI trial) and Cancer Research UK

CRUK Ref No: CRUK/11/055

Name of Sponsor: Velindre NHS Trust

Sponsor No: 2012/VCC/0037

Authorised by:			
Name:	Professor Dean Fennell	Role:	Chief Investigator
Signature		Date:	12/7/17
Name:	Professor Richard Adams	Role:	Director WCTU
Signature:		Date:	19/7/17

WCTU
WALES CANCER TRIALS UNIT
UNED YMCHWIL CANSER CYMRU

CONFIDENTIAL

This material is the property of the Wales Cancer Trials Unit. Do not disclose or use except as authorised.

General Information

This protocol describes the PIN clinical trial, and provides information about the procedures for entering participants into the trial. The protocol should not be used as a guide, or as an aide-memoire for the treatment of other patients. Every care has been taken in drafting this protocol; however, corrections or amendments may be necessary. These will be circulated to the known investigators in the trial, but centres entering patients for the first time are advised to contact the Wales Cancer Trials Unit (WCTU) in Cardiff to confirm that they have the most up-to-date version of the protocol in their possession. Problems relating to the trial should be referred, in the first instance, to the WCTU. Any deviation from the protocol should be immediately reported to WCTU.

Compliance

This trial will adhere to the conditions and principles which apply to all clinical trials as outlined in the Medicines for Human Use (Clinical Trials) Regulations 2004, as amended, EU Directive 2001/20/EC, EU Directive 2005/28/EC and the ICH Harmonised Tripartite Guideline for Good Clinical Practice (CPMP/ICH/135/95). It will be conducted in compliance with the protocol, the Medicines for Human Use (Clinical Trials) Regulations 2004 (SI2004/1031), as amended, the Research Governance Framework for Health and Social Care (Welsh Assembly Government November 2001 and Department of Health 2nd July 2005), the Data Protection Act 1998, and other regulatory requirements as appropriate.

Funding

The PIN trial is being funded by the AstraZeneca National Cancer Research Institute (NCRI) collaboration and by the Cancer Research UK Feasibility Study Committee and is thus part of the NCRN/NCRI portfolio of clinical trials. This study is supported by Cancer Research UK core funding at the WCTU.

Registration Line:

029 2075 5771

Serious Adverse Event (SAE) Email:

CTR-Safety@cardiff.ac.uk

CONFIDENTIAL
This material is the property of the Wales Cancer Trials Unit. Do not disclose or use except as authorised.

Trial Co-ordination

The PIN trial is being coordinated by the Wales Cancer Trials Unit (WCTU), an NCRI accredited, and United Kingdom Clinical Research Collaboration (UKCRC) registered trials unit.

This protocol has been developed by the PIN Trial Management Group (TMG) on behalf of the NCRI Lung Clinical Studies Group. Details of the TMG composition can be found on the WCTU website: www.wctu.org.uk

Wales Cancer Trials Unit
School of Medicine
Cardiff University
6th Floor, Neuadd Meirionnydd
Heath Park
Cardiff
CF14 4YS

Tel: +44 (0) 29 2068 7500
Fax: +44 (0) 29 2068 7501

Email: pin@cardiff.ac.uk
Website: www.wctu.org.uk

PIN trial staff

For all queries please contact the PIN Trial Manager. Any clinical queries will be directed through the Trial Manager to either the Chief Investigator or one of the clinical Co-Investigators.

Trial Manager:	Jo Canham	Tel: 029 2068 7581<br>Email: PIN@cardiff.ac.uk
Associate Director for Late Phase Trials:	Fergus Macbeth	c/o Wales Cancer Trials Unit
Director:	Richard Adams	Email : AdamsRA3@cardiff.ac.uk
Senior Statistician:	Angela Casbard	Email: casbardac@cardiff.ac.uk
Safety Desk:		Tel: 029 2068 7469<br>Email: CTR-Safety@cardiff.ac.uk

This material is the property of the Wales Cancer Trials Unit. Do not disclose or use except as authorised.

Chief Investigator

Professor Dean Fennell
Chair of Thoracic Medical Oncology
University of Leicester & Leicester University Hospitals
Hodgkin Building
PO Box 138, Lancaster Road
Leicester
LE1 9HN

Methodological Chief Investigator

Professor Gareth Griffiths
University of Southampton Clinical Trials Unit
MP 131, Southampton General Hospital
Tremona Road
Southampton
Hampshire
SO16 6YD

Co-investigators

Dr Jason Lester
Velindre Cancer Centre
Velindre Road
Cardiff
Glamorgan
CF14 2TL

Dr Marianne Nicolson
Consultant Medical Oncologist
ANCHOR Unit - Clinic D
Aberdeen Royal Infirmary
Foresterhill
Aberdeen
AB25 2ZN

Professor Fiona Blackhall
Christie NHS Foundation Trust
Clinical and Experimental Pharmacology
Paterson Institute for Cancer Research
Wilmslow Road
Manchester
M20 4BX

Professor Sarah Danson
Senior Clinical Lecturer
University of Sheffield
Weston Park Hospital
Whitham Road
Sheffield
S10 2SJ

Professor Samreen Ahmed
University Hospitals of Leicester NHS Trust
Leicester
LE1 5WW

CONFIDENTIAL
This material is the property of the Wales Cancer Trials Unit. Do not disclose or use except as authorised.

Advisors

Dr Jacqui Shaw
Senior Lecturer
Cancer Studies and Molecular Medicine
University of Leicester

Pharmacy representative
Renata Poole
Velindre Cancer Centre
Whitchurch
Cardiff
CF14 2TL

Research Nurse Representative
Louise Nelson
Hope Clinical Trials Unit
Level 2, Osborne Building
Leicester Royal Infirmary
Leicester
LE1 5WW

Consumer representatives

Harold Toone
c/o WCTU

Allan Barham
c/o WCTU

Contents

Trial Co-ordination **3**
Abbreviations and glossary **8**
1.0 Trial schema **11**
2.0 Trial synopsis **12**
2.1 Lay summary........ 17
3.0 Background, rationale and objectives **18**
4.0 Study design **21**
4.1 Risk Assessment 22
5.0 Participating centre selection **23**
6.0 Trial Entry **24**
6.1 Stage 1 Screening procedures 24
6.1.1 Inclusion criteria – Stage 1 24
6.1.2 Exclusion criteria – Stage 1........ 25
6.1.3 Participant Registration into stage 1 of the trial 25
6.2 Participant Randomisation into the trial 25
6.2.1 Randomisation Screening Procedures 26
6.2.2 Inclusion criteria – Randomisation........ 26
6.2.3 Exclusion criteria – Randomisation 27
6.2.4 Participant Randomisation into the trial........ 28
6.3 Informed consent 28
7.0 Trial treatments **30**
7.1 Dose interruptions and modifications........ 31
7.2 Management of toxicities........ 32
7.3 Drug supply and distribution 33
7.4 Drug accountability 34
7.5 Concomitant medications 35
7.6 Code breaking procedure 35
8.0 Trial assessments **36**
8.3 Pre-Randomisation screening procedures 36
8.4 During Maintenance Therapy (every 3 weeks) until disease progression, or up to 1 year post randomisation........ 36
8.5 Upon radiological disease progression, or up to 1 year post randomisation 37
8.6 30 days post-treatment visit........ 37
8.7 Completion of CRFs 37
8.8 Schedule of trial assessments 38
9.0 Safety reporting and pharmacovigilance **40**
9.1 Causality Assessments 41
9.2 Expectedness Assessments 42
9.3 SAE reporting........ 42
9.3.2 The WCTU responsibilities 43
9.4 SUSAR reporting........ 43
9.5 Unblinding for the purposes of SUSAR reporting 44
9.6 Safety Reports 44
9.7 Pregnancy reporting whilst participating in the PIN trial........ 45
9.8 Flowchart for Serious Adverse Event reporting........ 46

This material is the property of the Wales Cancer Trials Unit. Do not disclose or use except as authorised.

10.0 Trial management 47
10.1 Trial committees and trial management 47
10.2 Monitoring 47
10.2.1 Site monitoring 47
10.2.2 Central monitoring and data queries 48
10.3 Participant withdrawal 48
10.4 Lost to follow up 49
10.5 Protocol/GCP non-compliance 49
10.6 The End of the Trial 49
10.7 Archiving 49
11.0 Statistical considerations 50
11.1 Randomisation 50
11.2 Outcome measures 50
11.2.1 Primary outcome measure 50
11.2.2 Secondary outcome measures 50
11.3 Sample size calculation 50
11.4 Statistical analyses 50
11.5 Subgroup analyses 51
12.0 PIN Translational research 52
13.0 Publication policy 52
14.0 Informed consent, ethical and regulatory considerations 53
14.1 Ethical approval 53
14.2 Clinical Trial Authorisation (CTA) 53
14.3 Regulatory considerations 53
14.4 Research governance approval 53
14.5 Sponsorship 53
14.5 Indemnity 54
14.6 Data protection 54
14.7 Finance 54
15.0 References 56
Appendix A: A guide to performing causality assessments 58

This material is the property of the Wales Cancer Trials Unit. Do not disclose or use except as authorised.

Abbreviations and glossary

**AE**	Adverse Event
**ALK**	Anaplastic Lymphoma Kinase
**AML**	Acute myeloid leukaemia
**AUC**	Area under the curve
**bd**	Twice daily
**CI**	Chief Investigator
**CNS**	Central nervous system
**CRF**	Case report form
**CR-UK**	Cancer Research UK
**CT**	Computerised axial tomography
**CTA**	Clinical Trial Authorisation
**CTCAE**	Common Terminology Criteria for Adverse Events
**DH**	Department of Health
**DNA**	Deoxyribonucleic acid
**ECG**	Electrocardiogram
**EGFR**	Epidermal Growth Factor Receptor
**EMA**	European Medicines Agency
**EML4**	Echinoderm microtubule-associated protein-like 4
**EudraCT**	European Union Drug Regulatory Agency Clinical Trial
**FDA**	Food and Drug Administration
**FRS**	Fellow of the Royal Society
**GCP**	Good Clinical Practice
**GI**	Gastrointestinal
**GP**	General Practitioner
**IB**	Investigator's Brochure
**ICH**	International Conference on Harmonisation
**ICH-GCP**	International Conference on Harmonisation – Good Clinical Practice
**IDMC**	Independent Data Monitoring Committee
**IMP**	Investigational medicinal product

CONFIDENTIAL
This material is the property of the Wales Cancer Trials Unit. Do not disclose or use except as authorised.

**ISF**	Investigator Site File
**ISRCTN**	International Standard Randomised Controlled Trial Number
**IWRS**	Interactive Web Response System
**MDS**	Myelodysplastic syndrome
**MHRA**	Medicines and Healthcare products Regulatory Agency
**MRC**	Medical Research Council
**MREC**	Multi-centre research ethics committee
**mRNA**	Messenger Ribonucleic Acid
**NCRI**	National Cancer Research Institute
**NCRN**	National Cancer Research Network
**NSCLC**	Non-Small Cell Lung Cancer
**NHS**	National Health Service
**od**	Once daily
**OS**	Overall survival
**PARP**	Poly (ADP-ribose) polymerase
**Participant**	An individual who has given written informed consent and is participating in trial related activities
**Patient**	A patient under care who may be eligible for the trial but has not yet consented to participate in any trial related activities.
**PI**	Principal Investigator
**PIS**	Participant Information Sheet
**po**	Peroral (by mouth)
**PFS**	Progression Free Survival
**q21**	21 day cycle
**R&D**	Research and Development
**REC**	Research Ethics Committee
**RECIST**	Response Evaluation Criteria In Solid Tumours
**SAE**	Serious adverse event
**SAR**	Serious adverse reaction
**SOP**	Standard operating procedure
**SPC**	Summary of Product Characteristics

CONFIDENTIAL
This material is the property of the Wales Cancer Trials Unit. Do not disclose or use except as authorised.

**SUSAR**	Suspected unexpected serious adverse reaction
**TKI**	Tyrosine Kinase Inhibitor
**TMF**	Trial Master File
**TMG**	Trial Management Group
**TSC**	Trial Steering Committee
**TSF**	Trial Site File
**Vital signs**	Weight, pulse, blood pressure, oxygen saturation.
**WBC**	White blood cell
**WCTU**	Wales Cancer Trials Unit

CONFIDENTIAL
This material is the property of the Wales Cancer Trials Unit. Do not disclose or use except as authorised.

1.0 Trial schema

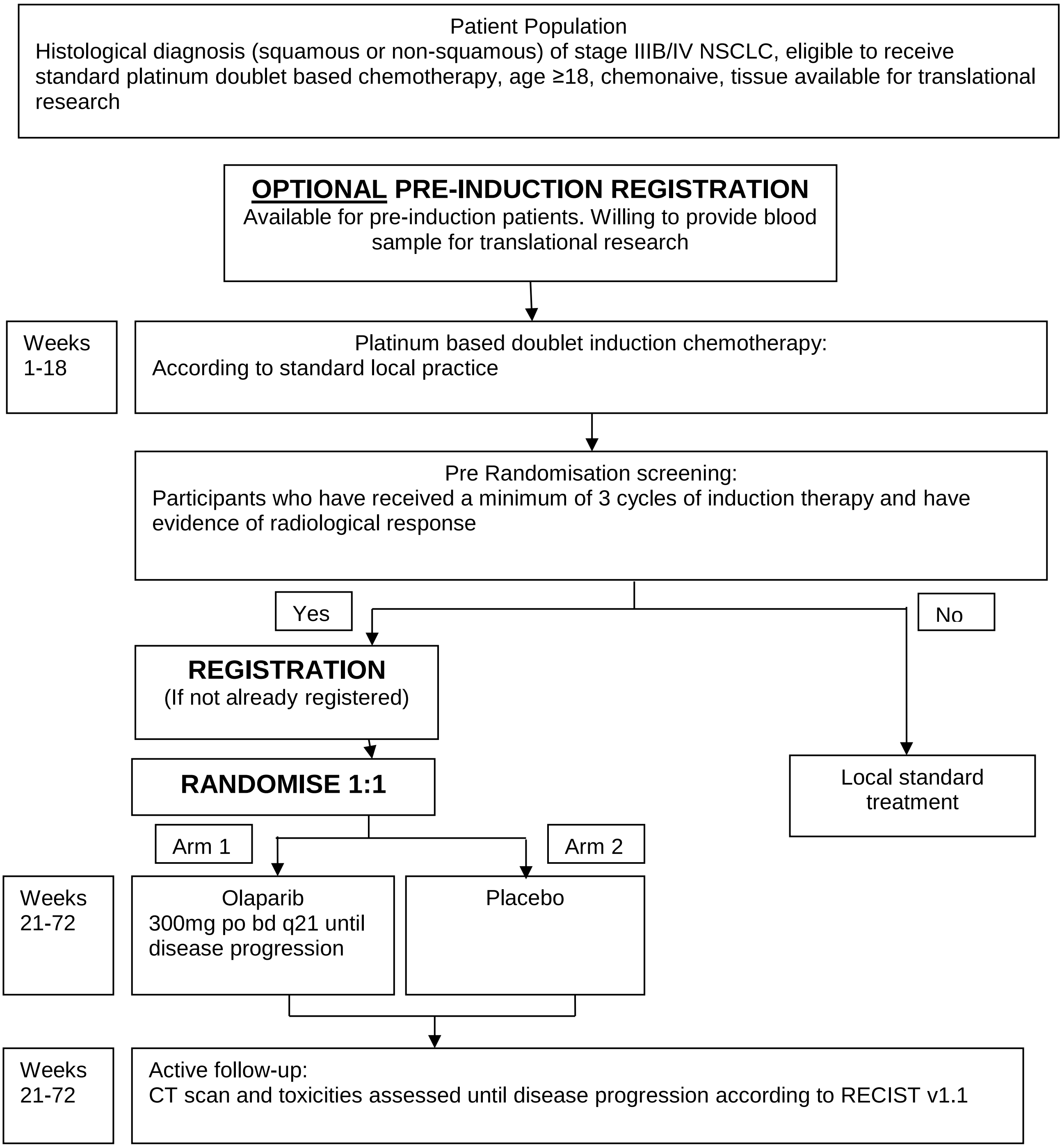

CONFIDENTIAL
This material is the property of the Wales Cancer Trials Unit. Do not disclose or use except as authorised.

2.0 Trial synopsis

**Study title:**	A Randomised phase II trial of Olaparib maintenance versus placebo monotherapy in patients with non-small cell lung cancer.				
**Study acronym:**	PIN				
**Short title:**	Parp Inhibitor in Advanced NSCLC				
**EudraCT No:**	2012-003383-51				
**Clinicaltrials.gov No:**	NCT01788332				
**ISRCTN No:**	42518913				
**Funder:**	Cancer Research UK and AstraZeneca	**Funder's No:**	CRUK/11/055		
**Chief Investigator:**	Professor Dean Fennell				
**Sponsor:**	Velindre NHS Trust	**Sponsor No:**	2012/VCC/0037		
**Study period:**	24 months	**Phase:**	II	**Number of arms:**	2
**Number of participants:**	68 patients will be randomised.<br>25 sites are expected to participate				

Objectives

Primary Objectives:

- To establish the anti-tumour activity of maintenance Olaparib as measured by PFS in DNA damage sensitive NSCLC

Secondary Objectives:

- To determine the toxicity profile of maintenance Olaparib
- To determine the feasibility of use of maintenance Olaparib
- To collect tumour and blood samples for future translational work including investigating BRCA1 expression and genetic determinants of Olaparib sensitivity
- To determine other anti-tumour activity as measured by radiological response, and overall survival.

CONFIDENTIAL
This material is the property of the Wales Cancer Trials Unit. Do not disclose or use except as authorised.

Optional registration into stage 1 – the translational sub-study

Main inclusion criteria for stage 1 (see section 6.1.1 for full list)

- Confirmed histological diagnosis of either squamous or non-squamous NSCLC allowed
- Diagnosis of Stage IIIB/IV NSCLC
- No prior systemic chemotherapy for advanced NSCLC. Prior treatment with an oral tyrosine kinase inhibitor for EGFR/ALK mutant lung cancer is allowed. Immunotherapy e.g. with a PD1 or PDL1 targeted agent is allowed
- Fit to receive platinum doublet induction chemotherapy
- Age >= 18
- Willing to consent to provide tissue and plasma for translational research
- Informed consent

Main exclusion criteria for stage 1 (see section 6.1.2 for full list)

- Evidence of small cell, large cell neuroendocrine or carcinoid histology
- Serious or uncontrolled medical condition that would compromise adherence to the protocol
- Diagnosis of a second malignancy except adequately treated non-melanomatous skin cancer, or other cancer that is considered cured by surgical resection or radiation
- Previous treatment with PARP inhibitors
- Uncontrolled gastrointestinal disorders such as active diverticulitis or colitis
- Myelodysplastic syndrome (MDS) or acute myeloid leukaemia (AML)

Patients with radiological response to induction chemotherapy may proceed to stage 2 of the trial if they fulfil the Stage 2 entry criteria.

Patients without evidence of radiological response to induction chemotherapy:
If participants who have been registered to stage 1 do not meet the criteria for randomisation then they will be treated according to local standard clinical practice.

The best response to maintenance chemotherapy will be evaluated on completion of chemotherapy.

Patients registered post-induction chemotherapy will proceed to Stage 2 randomisation, according to the Stage 2 inclusion and exclusion criteria.

CONFIDENTIAL
This material is the property of the Wales Cancer Trials Unit. Do not disclose or use except as authorised.

Stage 2 of the study

Patients who have not yet been registered to Stage 1 must be registered prior to randomisation.

These patients must have had a minimum of three cycles of induction platinum doublet chemotherapy according to local standard practice.

Randomisation to Olaparib/placebo

Main inclusion criteria for stage 2 (see section 6.2.2 for full list)

- Confirmed diagnosis (either squamous or non-squamous NSCLC are allowed)
- Stage IIIB/IV NSCLC
- ECOG performance status 0-1
- Evidence of radiological response to induction chemotherapy. This can include mixed stable/response or evidence of tumour shrinkage that does not reach the threshold of "partial response" according to RECIST
- No prior systemic chemotherapy for advanced NSCLC. Previous adjuvant or neoadjuvant chemotherapy for non-advanced disease is acceptable. Prior treatment with an oral targeted therapy for e.g. EGFR/ALK or other driver-oncogene mutated lung cancer is allowed. Immunotherapy e.g. with a PD1 or PDL1 targeted agent is allowed.
- Adequate organ function
- Willing to use adequate contraception if of reproductive ability
- Informed consent
- Randomisation to be a maximum of 42 days from day 1 of the last cycle of induction chemotherapy

Main exclusion criteria for stage 2 (see section 6.2.3 for full list)

- Small cell, large cell neuroendocrine or carcinoid histology
- Lack of response to induction chemotherapy (disease progression or no evidence of tumour shrinkage)
- Serious or uncontrolled medical condition that would compromise ability to adhere to protocol
- Diagnosis of a second malignancy except adequately treated non-melanomatous skin cancer, or other cancer that is considered cured by surgical resection or radiation
- Previous treatment with PARP inhibitors
- Difficulty swallowing
- CNS metastases (unless successful local therapy has been completed)
- Concurrent administration of any other systemic anti-tumour therapy
- Yellow fever vaccination in the 30 days prior to registration

CONFIDENTIAL
This material is the property of the Wales Cancer Trials Unit. Do not disclose or use except as authorised.

- Recent treatment with an agent that has not received regulatory approval
- Blood transfusion within 28 days prior to commencing Olaparib or a WBC <3 x 10^9/L
- Uncontrolled GI disorders
- MDS or AML
- Pregnant or breastfeeding

Treatment
Maintenance treatment

Experimental arm: Olaparib 300mg po bd administered until disease progression, and to commence within 42 days after day 1 of last cycle.

Control arm: Placebo, 2 tablets po bd until disease progression.

Trial assessments after consent taken:

Stage 1 Registration baseline – Patients recruited before chemotherapy:

- Medical history
- CT scan (standard pre-treatment)
- Inclusion and exclusion criteria check
- Translational blood sample
- Collection of diagnostic tumour tissue biopsy.

Chemotherapy details – All patients

- Chemotherapy treatment details

Stage 2 Pre-randomisation screening procedures

- Medical history (if not already registered in Stage 1)
- CT scan and RECIST assessment between day 1 of last cycle of chemotherapy and randomisation
- Pregnancy test
- Physical examination including vital signs and ECOG status
- Weight and height
- Blood pressure, pulse, oxygen saturation
- Urinalysis (pH, glucose, blood, protein and leucocytes)
- Full blood count
- Serum biochemistry (renal, liver and bone profiles)
- Documentation of concomitant medication
- Electrocardiogram (ECG)
- Assessment of inclusion and exclusion criteria
- Evaluation of toxicities using NCI CTCAE (v4.03) and real-time serious adverse event (SAE) reporting
- Translational bloods

CONFIDENTIAL
This material is the property of the Wales Cancer Trials Unit. Do not disclose or use except as authorised.

- Collection of diagnostic tumour tissue biopsy (if not already collected in Stage 1)

During Maintenance Therapy (every three weeks) until disease progression, treatment withdrawal, or up to 1 year post randomisation

- Documentation of dose delays and/or reduction
- CT scan to be conducted every six weeks until disease progression with full clinical examination to evaluate disease response by RECIST (v1.1) criteria
- Physical examination
- ECOG performance status
- Weight
- Blood pressure, pulse, oxygen saturation
- Urine dipstick (pH, glucose, blood, protein and leucocytes).
- Full blood count
- Serum biochemistry (renal, liver and bone profiles)
- Evaluation of toxicities using NCI CTCAE (v4.03) and real-time SAE reporting
- Documentation of concomitant medication
- Electrocardiogram (ECG) (**9 weeks after starting treatment**).

Upon radiological disease progression, or up to one year post randomisation

- Translational blood sample
- Tumour tissue biopsy (optional)

30 days post-treatment visit

- Physical examination
- ECOG performance status
- Weight
- Blood pressure, pulse, oxygen saturation
- Electrocardiogram (ECG)
- Evaluation of toxicities using NCI CTCAE (v4.03) and real-time SAE reporting

Endpoints:

Primary outcome measure:

Progression-free survival

Secondary outcome measures:

a. Safety, tolerability and feasibility of use
b. Objective response rate as assessed by RECIST (v1.1)
c. Overall survival, time from randomisation to death with those still alive censored at date last seen
d. Change in tumour volume– from randomisation to six weeks

CONFIDENTIAL
This material is the property of the Wales Cancer Trials Unit. Do not disclose or use except as authorised.

2.1 Lay summary

In 2010 more than 35,000 people died in the UK from lung cancer, the majority from non-small cell cancer (NSCLC). Chemotherapy is one of the main treatments for patients with advanced NSCLC but those treated will still only live for an average of 9 or 10 months after diagnosis. The purpose of this clinical trial is to find out whether or not giving a drug called Olaparib following chemotherapy will benefit patients with NSCLC who have responded to initial chemotherapy treatment by prolonging the time before the tumour re-grows. One hundred and fourteen patients who have responded to chemotherapy will be randomly allocated to receive either Olaparib or an inactive dummy 'placebo' tablet by mouth. The rationale for this clinical trial is that chemotherapy damages tumour cell DNA and that NSCLC tumours that respond to chemotherapy are less able to repair this damage. This can be exploited by using Olaparib, a drug which blocks an enzyme called Poly (ADP-ribose) polymerase (PARP), which is essential for DNA repair. This will prevent DNA repair and cause cancer cell death by a mechanism known as *synthetic lethality*. Synthetic lethality arises when a combination of mutations in two or more genes leads to cell death. If this study shows that Olaparib does delay disease progression, a larger more detailed clinical trial will be needed to find out whether using Olaparib actually makes patients live longer.

3.0 Background, rationale and objectives

BACKGROUND

Advanced non-small cell lung cancer (NSCLC) is a global health burden
Lung cancer kills over 1 million people annually worldwide; NSCLC accounts for around 90% of all lung cancer and kills 35,000 in the UK annually [1]. NSCLC is one of the leading causes of death worldwide and presents a huge health economic burden. The majority of patients with NSCLC present with locally advanced or metastatic (stage IIIB/IV) disease. Systemic treatment with platinum based doublet chemotherapy is the standard treatment for those fit enough to tolerate chemotherapy. However, median survival remains modest, at around 9-10 months. Only a minority of patients (20-30%) respond to chemotherapy [2], and those patients in whom their NSCLCs exhibit a combination of response and stable disease (i.e. disease control), live longer [3] .

Histological stratification of lung cancer therapy
A phase III trial [4] of 1,725 patients comparing gemcitabine/cisplatin with pemetrexed/cisplatin showed non-inferiority (HR=0.94, 95% CI=**0.84, 1.05**); however subgroup analysis showed a striking difference in survival based on histology. Pemetrexed was more effective in non-squamous NSCLC than gemcitabine which was more effective in squamous cell lung cancer. This led to "histology-restricted" approval for pemetrexed by the FDA and EMA. Although the mechanism underlying this differential clinical effectiveness is unknown, there is growing evidence that the expression of thymidylate synthase confers resistance to pemetrexed and is expressed at a higher level in squamous cell NSCLC [5].

Molecular stratification of NSCLC is the new therapeutic paradigm
In recent years, it has become widely recognised that NSCLC is a heterogeneous cancer, an observation that has implications for the development of effective therapy. Until recently, the treatment of Stage IIIB/IV NSCLC with chemotherapy was considered to be at a plateau because of the finding in a four arm randomised clinical trial of 1,155 patients [6] that no-platinum doublet appeared more effective than any other. Recently the idea is emerging that it is best to tailor treatment for individual patients based on the morphological or molecular characteristics of their tumour; so called 'targeted' therapy.

For example, in 2004 NSCLC-specific activating mutations of the epidermal growth factor receptor (EGFR) were found to be correlated with extreme sensitivity to the oral tyrosine kinase inhibitors (TKIs) gefinitib and erlotinib both in the clinic and in the laboratory [7]. Previously when these drugs were investigated in global phase III clinical trials in combination with standard chemotherapy in *unselected* lung cancer patients they had not been shown to be effective [8].

Mutation of the EGFR receptor confers a phenotype to the NSCLC cell called 'oncogene addiction'. This phenomenon involves a dependence on mutant EGFR ($EGFR^{mut}$) for survival [6, 7] . Inhibition of constitutive signalling through the EGFR leads to essential activation of the death inducing protein BIM [8-10], resulting in activation of mitochondrial apoptosis. Based on this discovery, a randomized phase III trial including 1217 patients with tumours with mutant EGFR [11] and comparing oral EGFR TKIs with chemotherapy showed significantly better 12 month progression-free survival (24.9% (TKIs) vs 6.7% (chemotherapy), HR 0.74 (95% CI 0.65,0.85 p<0.001)). Treating patients with NSCLC and mutant EGFR

This material is the property of the Wales Cancer Trials Unit. Do not disclose or use except as authorised.

with TKIs has become standard. But this subgroup represents only 10 to 15% of patients with NSCLC, and almost all have non-squamous tumours.

In 2007, a second major molecular subclass of IIB/IB NSCLC was identified harbouring translocation of echinoderm mesenchymal lymphoma 4 (EML4) with anaplastic lymphoma kinase (ALK) [12]. This mutation (EML4-ALK) occurs in around 5% of lung cancers and early clinical trials have investigated the inhibition of ALK through TKIs. In common with EGFR TKIs, the dramatic apoptosis induction in this specific phenotype results from activation of BIM by post-translational, extracellular-signal-regulated-kinase (ERK)- dependent phosphorylation and activation. ALK inhibition by the TKI crizotinib produces very high response rates and this impressive early clinical activity prompted application for accelerated approval only four years after initial molecular discovery [13]. EML4-ALK gene rearrangement illustrates the potential of personalised therapy to accelerate the path from basic discovery to approval.

Non-squamous NSCLC can be further subdivided into several molecular subclasses in addition to mutation of EGFR and ALK gene rearrangement; these include mutation of KRAS, BRAF, Human epidermal Growth Factor Receptor 2 (Her2), cMET, and PIK3CA. For several of these gene alterations, targeted therapies exist, raising the possibility that the future of NSCLC therapy will involve the selective targeting of small subpopulations with effective therapy. Many of these mutations (with the exception of PIK3CA) are mutually exclusive. Accordingly these have been termed ‘driver mutations’. Molecular diagnostic platforms are being developed to enable multiple analysis of somatic gene alterations. Analysis of over 1,000 NSCLCs by the US Lung Cancer Mutation Consortium recently showed that mutations could be identified around 60% of which were potentially treatable with targeted therapies. Much of the advance in molecular stratification in NSCLC has come from the study of non-squamous lung cancer. However, DNA sequencing initiatives, which are used to accurately screen the genome, are rapidly identifying potentially targetable somatic gene alterations in squamous lung cancer. These include somatic gene alterations in DDR2 and FGFR3, which may be targeted with small molecules.

Synthetic lethality **: a new therapeutic opportunity in NSCLC**

In contrast to oncogene addiction such as in the case of EGFR inhibitor treatment, synthetic lethality is a promising therapeutic strategy. Mutation of BRCA1 causes defective DNA repair. BRCA1 mutant cancers rely on PARP to repair DNA following DNA damage. BRCA1 mutant tumours are therefore exquisitely sensitive to PARP inhibition [14, 15]. This is an example of synthetic lethality. Loss of BRCA1 expression measured using immunohistochemistry occurs in around 15% of NSCLCs [16]. In preclinical models, where BRCA1 expression is silenced, defective homologous recombination occurs leading to marked (log-fold) sensitization to PARP inhibition. Importantly, in contrast to cell death induced by ALK or EGFR inhibition which results in mitochondrial apoptosis dependent on the proapoptotic protein BIM [8-10], we have shown that death induced by PARP inhibitor mediated synthetic lethality in BRCA1 silenced NSCLC does not require a functional mitochondrial pathway [15]. This means that many important survival proteins that inhibit chemotherapy induced cancer cell death via this pathway, cannot prevent PARP inhibitor induced cell death. Rather, death occurs through catastrophic accumulation of DNA double strand breaks. Loss of BRCA1 is not restricted to a specific histological subtype but is reflected to a similar degree in both squamous as well as non-squamous non-small cell lung cancers [15].

CONFIDENTIAL
This material is the property of the Wales Cancer Trials Unit. Do not disclose or use except as authorised.

Rationale for a switch maintenance clinical trial of PARP inhibition in platinum doublet responders

By regulating DNA repair (homologous recombination), BRCA1 expression levels regulate sensitivity to platinum chemotherapy [16-18]. Studies exploring the correlation between BRCA1 mRNA level and response to platinum based therapy have shown a strong inverse predictive interaction [19-20] However, it is believed that low BRCA1 levels are likely to account for only a proportion of highly platinum responsive NSCLCs, with potential defects in other homologous recombination regulating genes underpinning sensitivity. This is because emerging evidence from functional genomic studies indicate that synthetic lethality to PARP inhibition can occur in the absence of somatic alterations in BRCA1 function [21], eg. PTEN deficiency[22, 23], aurora A expression [24].

The total number of cycles of platinum-based chemotherapy that can be given to patients with stage IIIB/IV NSCLC is between 4 and 6, because of toxicity. But if effective therapy could be maintained, disease control, quality of life and overall survival might be prolonged. Exploiting somatic homologous recombination defects, including loss of BRCA1 expression, should confer sensitivity both to PARP inhibition targeted at the cancer and to platinum-based chemotherapy. So response to initial optimal platinum based chemotherapy could be used to identify those patients likely to have DNA repair deficient NSCLC. They could then receive cancer-specific therapy with PARP inhibitor to exploit the homologous recombination defect and to induce NSCLC cell synthetic lethality.

Olaparib

Olaparib (AZD2281, KU-0059436) is a potent inhibitor of poly (ADP-ribose) polymerase enzyme (PARP), (molecular weight 434) which is being developed as a monotherapy as well as for combination with chemotherapy and other anti-cancer agents. Olaparib can lead to tumour regression in patients with DNA repair deficient NSCLC. Olaparib may also enhance the DNA damaging effects of chemotherapy. Within the clinical development programme for Olaparib, the intention is to assess tolerability and efficacy of Olaparib in patients with advanced DNA repair deficient solid tumours, such as hereditary BRCA-associated cancers and serous ovarian cancers. In such tumour types, Olaparib may offer a potentially efficacious and less toxic cancer treatment compared to currently available chemotherapy regimens.

A recent UK phase IIb trial of maintenance Olaparib (treatment with Olaparib following completion of induction chemotherapy) in patients with ovarian cancer met its primary endpoint of progression-free survival (HR, 0.35; 95% CI 0.25–0.47; P<0.00001; median 8.3 vs 3.7 months) [25]. This trial closely resembles the proposed PIN study, which strengthens the rationale for our trial design.

Safety profile of Olaparib

As of 14 February 2012, 1785 patients with ovarian, breast, pancreatic, gastric and a variety of other solid tumours are estimated to have received treatment with Olaparib across the dose range 10 mg od to 600 mg bd. Olaparib has been given as either monotherapy (13 studies, 1049 estimated patients) or in combination with other chemotherapy/anti-cancer agents (17 studies, 736 estimated patients). The majority of patients to date have received the capsule formulation of Olaparib. Data from these studies indicate that Olaparib is generally well tolerated at doses up to 400 mg bd (in the capsule formulation) in patients with solid tumours. Approximately 235 patients have received the tablet formulation to date at several different doses and schedules.

CONFIDENTIAL
This material is the property of the Wales Cancer Trials Unit. Do not disclose or use except as authorised.

Monotherapy has been associated with the following toxicities: haemotological toxicities, nausea and vomiting, diarrhoea, dyspepsia, dysgeusia, fatigue, headache and dizziness. Pneumonitis events with no consistent clinical pattern have been reported in a small number of patients. MDS and AML have been reported in a small number of patients generally with extensive previous exposure to chemotherapy. Toxicities are usually mild or moderate, although some grade 3/4 toxicities have been reported for nausea, vomiting, fatigue and anaemia.

Objectives

Primary:

To investigate the anti-tumour activity of Olaparib as maintenance therapy as measured by PFS (time-to-event).

Secondary:

- To determine the toxicity of Olaparib as maintenance therapy.
- To establish the objective response rate as assessed by RECIST v1.1.
- To determine overall survival (OS), time from randomisation to death with those still alive censored at date last seen.
- To establish change in tumour volume – from randomisation to 6 weeks

4.0 Study design

This is a multicentre double blind randomised phase II trial. Patients have the option to be initially registered to a tissue collection study before induction chemotherapy. These patients will be asked to provide a translational blood sample pre-chemotherapy. All patients will be asked to consent to archival tissue collection for translational analysis, although the timing of this request will depend upon whether or not they have been registered to stage 1 initially. Another consent (to stage 2 of the trial) will precede randomisation to one of two groups of maintenance therapy (Olaparib or placebo) with 1:1 randomisation if they have evidence of radiological response following standard chemotherapy. This can include mixed stable/response or evidence of tumour shrinkage that does not reach the criteria of partial response according to RECIST.

Patients who are randomised to stage 2 of the trial will receive Olaparib or placebo until disease progression. They will be monitored by CT scan every two cycles until disease progression, when they will be managed according to local practice. Follow up will be for a maximum of 12 months from the point of randomisation or until disease progression. For patients who respond well to Olaparib or placebo, there is the option to continue to receive treatment beyond 60 weeks, provided there are no other safety or efficacy considerations, and provided that the PI has signed written confirmation that this is for the benefit of the patient.

Registered patients with progressive disease prior to randomisation will be asked to provide a follow-up blood sample at the end of induction chemotherapy. We will also ask for a blood sample upon randomisation for those patients who go through to the randomisation stage. However, patients with stable disease will not be asked for any further samples. All randomised patients for whom we have a

CONFIDENTIAL
This material is the property of the Wales Cancer Trials Unit. Do not disclose or use except as authorised.

baseline translational blood sample will be asked to provide a follow-up blood sample upon radiological progression.

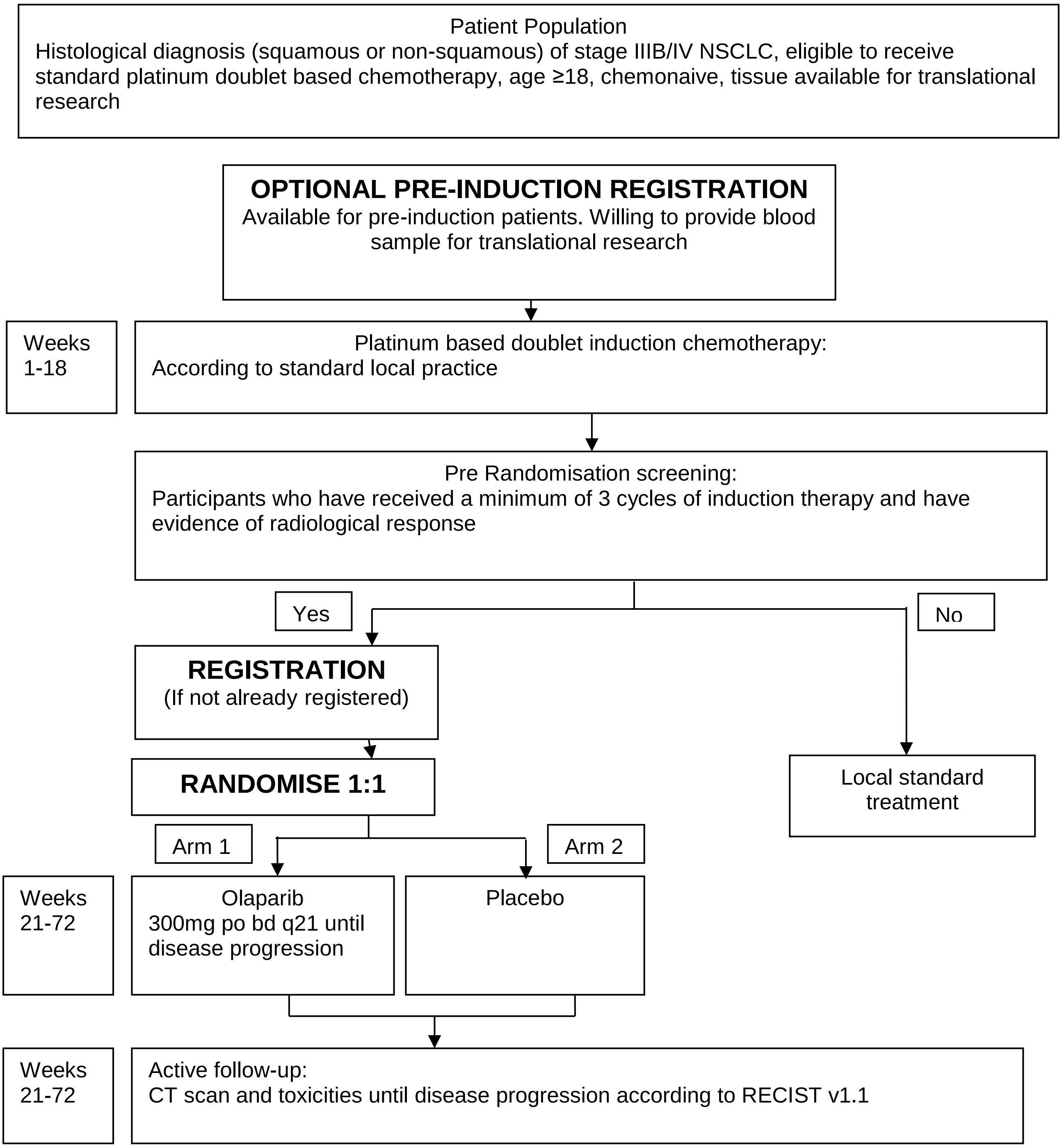

4.1 Risk Assessment

A Trial Risk Assessment has been completed by the WCTU to identify the potential hazards associated with the trial and to assess the likelihood of those hazards occurring and resulting in harm. This risk assessment has been completed in accordance with the MRC/DH/MHRA Joint project guidance

This material is the property of the Wales Cancer Trials Unit. Do not disclose or use except as authorised.

document 'Risk-adapted approaches to the management of Clinical Trials of Investigational Medicinal Products' and includes:

- The risk to participant safety in relation to the IMP.
- All other risks related to the design and methods of the trial (including risks to participant safety and rights as well as data integrity).
- The potential risks have been balanced against the level of risk that a trial participant would be exposed to outside of the trial. This trial has been categorised as a TYPE C where the level of risk is markedly higher than the risk of standard medical care. A copy of the trial risk assessment may be requested from the WCTU Trial Manager. The trial risk assessment is used to determine the intensity and focus of monitoring activity (see section 10.2).

5.0 Participating centre selection

This study will be carried out at participating centres within the UK. All centres that are interested in participating in the trial will be required to complete a registration form to confirm that they have adequate resources and experience to conduct the trial. We request that all centres taking part in PIN have the facilities to spin and store translational samples on site.

The following documentation must be completed and received by the WCTU in order for a centre to begin recruitment:

- Confirmation of NHS permission for research (site specific information application to NHS R&D).
- Confirmation of local R&D approval.
- Signed partnership agreement between the host care organisation and Sponsor.
- Current Curriculum Vitae and up to date GCP certificate of the PI.
- A copy of the most recent version of the Participant Information Sheet, Consent Form, Pregnancy Information Sheet and GP letters printed on the headed paper of the hospital trust.
- Completed Investigator Statement (signed and dated by the PI).
- Completed Delegation Log (signature list and delegation of responsibilities).
- Full contact details for all host care organisation personnel, indicating preferred contact.
- A set of laboratory normal ranges and accreditations from the host care organisation laboratory being used for analyses.
- Self-evident corrections log signed by PI.

Centre personnel are also required to attend either the trial launch meeting or an initiation teleconference prior to opening to recruitment.

Once all the documentation has been received at the WCTU, confirmation of centre approval will be sent by the WCTU to the centre PI. Case report forms (CRFs) will also be sent to the doctor, data manager or research nurse nominated as responsible for the participant.

All documentation must be stored in the Investigator Site File (ISF) at the site and in the Trial Site File (TSF) at the WCTU. The WCTU must be notified of any changes to the trial personnel and their

responsibilities during the running of the trial and the respective trial files must contain this up-to-date information.

6.0 Trial Entry

Any queries about whether a patient is eligible to enter the trial should be discussed with the WCTU before registration or randomisation. Any issues will then be raised with the Chief Investigator (CI) or one of the clinical Co-Investigators in the CI's absence. No eligibility waivers will be allowed.
The centre will inform the participant's General Practitioner (GP) of the participant's enrolment, if the participant gives consent to do so.

It may be possible for participants to be recruited into other clinical trials, but this should be discussed with the CI via the WCTU before this is considered.

Patients are eligible to be registered for the trial if all the inclusion criteria (Section 6.1.1) are met and none of the exclusion criteria (Section 6.1.2) apply. Patients who fulfil all of the randomisation inclusion criteria (Section 6.2.2) and none of the randomisation exclusion criteria (Section 6.2.3) may be randomised to receive either Olaparib or placebo. Patients who have not previously been registered to stage 1 of the trial will be registered prior to online randomisation, via the WCTU registration line.

6.1 Stage 1 Screening procedures

Before any trial related procedures are undertaken, the patient's written informed consent must be obtained. As the only trial specific procedure in stage 1 of the trial is tissue and blood collection, the patient may sign the consent form within 24 hours of being introduced to the trial if they wish. Consent to stage 1 of the trial may be taken by a Research Nurse or Clinician if they are delegated to do so on the trial delegation log.

The following procedures should be performed to confirm patient eligibility:

- CT scan of chest to be performed prior to induction chemotherapy. This will later be compared with the post-induction scan, in order to determine response.

This diagnostic scan is standard practice, used to ascertain eligibility, and it is therefore not necessary to collect participant consent prior to this procedure.

Positron emission tomography (PET)-CT scans are acceptable for inclusion, but CT scans should be used throughout the study for all subsequent treatment assessments.

6.1.1 Inclusion criteria – Stage 1

Patients meeting all of the following criteria may be registered into stage 1 of the trial:

1. Histological diagnosis of NSCLC. Histology can be either squamous or non-squamous. (If available, the same block or 10 unstained slides should be collected for translational research)
2. Stage IIIB or stage IV as defined by the American Joint committee on Cancer staging criteria (7th edition) for lung cancer, that is not amenable to curative therapy.

CONFIDENTIAL
This material is the property of the Wales Cancer Trials Unit. Do not disclose or use except as authorised.

3. Have had no prior systemic chemotherapy for advanced NSCLC. Previous adjuvant or neoadjuvant chemotherapy for non-advanced disease is acceptable. Prior treatment with an oral targeted therapy for e.g. EGFR/ALK or other driver- oncogene mutated lung cancer is allowed. Immunotherapy e.g. with a PD1 or PDL1 targeted agent is allowed.
4. Patients who have already started their induction chemotherapy are not eligible for stage 1 of the trial **but may still be able to enter post-chemotherapy for maintenance olaparib.** Patients who have had adjuvant therapy and then progressed after a year of completing adjuvant therapy **are eligible**.
5. Eligible to receive standard platinum doublet-based chemotherapy.
6. Men or women, aged 18 or over.
7. Willing to consent to provide tissue and blood for translational research.
8. Patients must provide informed consent prior to any study specific procedures.

6.1.2 Exclusion criteria – Stage 1

If any of the following criteria apply, patients cannot be included in the trial:

1. Evidence of small cell, large cell neuroendocrine or carcinoid histology.
2. Have a serious or uncontrolled medical condition that in the opinion of the investigator would compromise the patient's ability to adhere to the protocol.
3. Diagnosis of a second malignancy (except adequately treated non-melanomatous skin cancer, or other cancer that is considered cured by surgical resection or radiation). Patients who had another malignancy in the past but have been disease free for more than 5 years are eligible.
4. Previous treatment with PARP inhibitors.
5. Uncontrolled gastrointestinal disorders such as active diverticulitis or colitis, or any major GI resection which could have an impact on patients' ability to absorb Olaparib.
6. Myelodysplastic syndrome (MDS) or acute myeloid leukaemia (AML).

The PI must confirm the eligibility of a patient in the patient's medical notes prior to registration.

6.1.3 Participant Registration into stage 1 of the trial

To register the patient ring the WCTU registration line on 02920 755 771 between 0900 and 1700 hours (Monday to Friday). The patient will be allocated a unique identification number at registration by the WCTU. This will be the patient's trial number throughout the remainder of the study.

For patients consenting to Stage 1, registration must take place prior to commencing induction chemotherapy.

6.2 Participant Randomisation into the trial

For patients consenting to Stage 2 who were not registered in Stage 1, the patient should be registered with WCTU after checking eligibility but before being randomised.

CONFIDENTIAL
This material is the property of the Wales Cancer Trials Unit. Do not disclose or use except as authorised.

6.2.1 Randomisation Screening Procedures

Following completion of induction chemotherapy, the participant's research nurse and doctor or other treating clinician will screen the participant to ensure that they meet the randomisation eligibility criteria.

The following procedures should be performed between day 1 of final induction chemotherapy cycle and randomisation:

- CT scan of chest to assess measurable disease.

The following procedures should be performed within 14 days prior to randomisation to confirm patient eligibility:

- Medical history
- Documentation of concomitant medication
- Physical examination
- ECOG performance status
- Weight and height
- Blood pressure, pulse, oxygen saturation
- Full blood count
- Serum biochemistry (renal, liver and bone profiles)
- Urinalysis (pH, glucose, blood, protein and leucocytes)
- Electrocardiogram (ECG)
- Pregnancy test in women of child bearing potential within 3 days prior to randomisation

There should be no more than 42 days between day 1 of the last cycle of induction chemotherapy and starting Olaparib/placebo.

6.2.2 Inclusion criteria – Randomisation

1. Confirmed diagnosis of NSCLC (either squamous or non-squamous). Stage IIIB or stage IV as defined by the American Joint committee on Cancer staging criteria (7th edition) for lung cancer, that is not amenable to curative therapy.
2. ECOG performance status 0-1
3. Evidence of radiological response to induction chemotherapy, from the pre-treatment baseline. This must be assessed by the PI. This can include mixed stable/response or evidence of tumour shrinkage that does not reach the criteria of partial response according to RECIST.
4. Have had no prior systemic chemotherapy for advanced NSCLC. Previous adjuvant or neoadjuvant chemotherapy for non-advanced disease is acceptable. Previous palliative radiotherapy to non-target metastases is allowed provided no more than 25% of the bone marrow volume is irradiated. Irradiated sites cannot include the sites of measurable disease unless clear tumour progression has been documented in them since the end of radiation therapy. Patients who have had adjuvant therapy and then progressed after a year of completing adjuvant therapy are eligible. Patients who have received an oral inhibitor for molecularly stratified subgroups e.g. EGFR or ALK mutated

This material is the property of the Wales Cancer Trials Unit. Do not disclose or use except as authorised.

lung cancer, are allowed. Immunotherapy e.g. with a PD1 or PDL1 targeted agent is allowed.

5. Adequate organ function, including the following:
   a. Adequate bone marrow reserve: White cell count ≥ **3.0** x 10^9/L, absolute neutrophil count (ANC) ≥ **1.5** x 10^9/L, platelets ≥ **100** x 10^9/L, haemoglobin ≥ **90**g/L.
   b. Hepatic: total bilirubin ≤ 1.5 times the upper limit of normal (x ULN); alkaline phosphatase (ALP), aspartate aminotransferase (AST), or alanine aminotransferase (ALT) ≤ 2.5 x ULN. ALP, AST, and ALT ≤ 5 x ULN is acceptable if the liver has tumour involvement.
   c. Renal: calculated creatinine clearance (CrCl) ≥ **50**mL/min using Cockcroft-Gault or Wright formula, serum creatinine ≤ 1.5 x institutional upper limit of normal (ULN).
   d. If blood count/morphology suggestive of MDS/AML, no features suggestive of MDS/AML on peripheral blood smear.
6. Patients with reproductive potential (male or female), who are sexually active for the duration of the trial or the drug washout period, should be prepared to use two effective forms of contraception throughout their participation in the trial and for at least three months after the last dose of Olaparib. Effective forms of contraception would include condom with spermicide, along with one of the following: oral contraceptive or hormonal therapy (e.g. hormone implants); placement of an inter-uterine device; vasectomy with assurance of post-vasectomy confirmation of azoospermia; tubal occlusion. Accepted hormonal methods include: Etonogestrel implants; normal and low dose combined oral pills; norelgestromin/ethinyl estradiol transdermal system; or cerazette.
7. Men or women, aged 18 or over.
8. Willing to consent to provide tissue and blood for translational research.
9. Patients must provide informed consent prior to any study specific procedures.
10. There should be no more than 42 days between day 1 of the last cycle of induction chemotherapy and starting Olaparib/placebo.

6.2.3 Exclusion criteria – Randomisation

1. Evidence of small cell, large cell neuroendocrine or carcinoid histology.
2. Patients with radiological disease progression or stable disease after induction chemotherapy
3. Have received treatment with an agent that has not received regulatory approval, within 30 days of study entry.
4. Have had a blood transfusion within 28 days prior to commencing Olaparib or have a WBC <3 x 10^9/L
5. Have received yellow fever vaccination in the 30 days prior to randomisation.
6. Difficulty swallowing.
7. Have central nervous system (CNS) metastases (unless the patient has completed successful local therapy for CNS metastases e.g. involving complete surgical removal or radical radiotherapy to a solitary CNS metastasis). A screening CT scan or magnetic resonance imaging to assess for brain metastases in the absence of a clinical suspicion is not required.

CONFIDENTIAL
This material is the property of the Wales Cancer Trials Unit. Do not disclose or use except as authorised.

8. Concurrent administration of any other systemic antitumour therapy.
9. Have a serious or uncontrolled medical condition that in the opinion of the investigator would compromise the patient's ability to adhere to the protocol.
10. Diagnosis of a second malignancy (except adequately treated non-melanomatous skin cancer, or other cancer that is considered cured by surgical resection or radiation). Patient who had another malignancy in the past but have been disease free for more than 5 years are eligible.
11. Previous treatment with PARP inhibitors.
12. Uncontrolled gastrointestinal disorders such as active diverticulitis or colitis, or any major GI resection which could have an impact on patients' ability to absorb Olaparib.
13. Myelodysplastic syndrome (MDS) or acute myeloid leukaemia (AML).
14. Are pregnant or breastfeeding. If a participant becomes pregnant during the trial, Olaparib/placebo must be discontinued and the participant followed up until birth or termination of pregnancy. Breastfeeding must be avoided as it is unknown whether Olaparib is excreted in human milk

6.2.4 Participant Randomisation into the trial

Once the eligibility criteria have been confirmed, the participant may be registered (if not already registered in stage 1) and randomised. To register the patient ring the WCTU registration line on **029 20755 771** between 0900 and 1700 hours (Monday to Friday). The patient will be allocated a unique identification number at registration by the WCTU. This will be the patient's trial number throughout the remainder of the study.

Please make sure all patients are registered prior to randomisation. To randomise the patient, log onto the Interactive Web Response System (IWRS) and follow the instructions on-screen and in the IWRS user guide. Upon randomisation, a confirmatory email will be sent to the research nurse and the trial pharmacist. This will confirm the participant's date of birth, their trial ID number, the kit number for the Olaparib/placebo, and the date and time of randomisation. Please print and file the email in the individual trial specific patient file.

6.3 Informed consent

The patient's written informed consent must be obtained prior to registration and/or randomisation using the PIN trial Consent Forms, which follow the Participant Information Sheets. There are three separate PISs: one for patients registering to Stage 1 prior to their chemotherapy; one for patients being randomised to Stage 2 who were previously registered to Stage 1 prior to their chemotherapy; and one for patients being registered and randomised to Stage 2 following chemotherapy. Registration into the tissue collection stage (stage 1) may take place within 24 hours of the initial invitation to participate, but randomisation into stage 2 should only take place once the patient has been given a minimum of 24 hours to consider the trial before being asked to sign the Consent Form. Please note, only when written informed consent has been obtained from the patient can they be considered a trial participant.

CONFIDENTIAL
This material is the property of the Wales Cancer Trials Unit. Do not disclose or use except as authorised.

Patients will also be asked to consent to NHS Information Centre Flagging (and Scottish and Northern Irish equivalents) so that the date and cause of death can be collected without longer term follow-up. This will be optional and additional to the standard informed consent.

The patient's consent to participate in the trial should be obtained by the treating doctor (or Research Nurse in the case of Stage 1) after a full explanation has been given of the treatment options, including the conventional and generally accepted methods of treatment. All patients must be informed of the aims of the study, the possible adverse events, the procedures and possible hazards to which they may be exposed. They will be informed of the strict confidentiality of their patient data, but that their medical records may be reviewed for trial purposes by authorised individuals other than their treating physician.

Patient's consent will be sought to notify their GP of their involvement in the trial. The investigator should determine that the patient is fully informed of the trial and their participation, is in accordance with the principles of Good Clinical Practice. Patients should always be asked to sign a consent form. One copy should be given to the participant but the original copy should be kept in the investigator site file and a further copy should be kept with participant's hospital notes.

Participant consent is requested to collect NHS Numbers to utilise NHS data for future research, through Cancer Research UK and the National Cancer Intelligence Network (NCIN).

The right of the participant to refuse to participate in the trial without giving reasons must be respected. After the patient has entered the trial, the investigator must remain free to give alternative treatment to that specified in the protocol, at any stage, if he/she feels it to be in the best interest of the participant. However, the reason for doing so should be recorded and the participant will remain within the trial for the purpose of follow up and data analysis according to the treatment option to which he/she has been allocated. Similarly, the participant must remain free to withdraw at any time from the protocol treatment without giving reasons and without prejudicing his/her further treatment.

CONFIDENTIAL
This material is the property of the Wales Cancer Trials Unit. Do not disclose or use except as authorised.

7.0 Trial treatments

Stage 1: Optional registration into tissue collection study, followed by Induction Chemotherapy

Platinum doublet induction chemotherapy will be administered in accordance with local practice.

The number of chemotherapy cycles will depend on how well the patient is responding. As per local standard practice, an interim CT scan should be carried out at cycle 2 or 3 and compared to the baseline CT scan performed prior to registration to decide how many more cycles to give. This will then trigger planning of the next CT scan and disease assessment which should be arranged at the end of induction chemotherapy. Those who have progressive or stable disease, (when comparing pre-induction chemotherapy scan to final induction chemotherapy scan) are ineligible for randomisation, and should be treated as per local practice outside of trial protocol treatment. A translational blood sample should be collected from patients with progressive disease.

Participants with a radiological response after chemotherapy are eligible for randomisation (provided they fulfil the other eligibility criteria), and should start treatment within 42 days of day 1 of the last induction treatment. There should be no more than 7 days between randomisation and starting treatment.

Stage 2: Olaparib monotherapy/placebo

If patients have not previously been registered into stage 1 of the study, then they should be registered following induction chemotherapy and prior to randomisation to stage 2.

300mg bd administered and reviewed in 21-day cycles until disease progression, unacceptable toxicity or patient withdrawal of consent.

300mg (consisting of 2x150mg tablets) should be taken twice daily, in the morning and evening, unless otherwise instructed, with approximately 240ml of water. The tablets should be taken at least one hour after food, and patients should refrain from eating preferably for up to two hours afterwards because of the potential effect of food on absorption. The Olaparib tablets should be swallowed whole and not chewed, crushed, dissolved or divided. If vomiting occurs shortly after the Olaparib or placebo tablets are swallowed, the dose should only be replaced if all of the intact tablets can be seen and counted. Should any patient enrolled on the study miss a scheduled dose for whatever reason (e.g., as a result of forgetting to take the tablets or vomiting), the patient will be allowed to take the scheduled dose up to a maximum of 2 hours after that scheduled dose time. If greater than 2 hours after the scheduled dose time, the missed dose is not to be taken and the patient should take their allotted dose at the next scheduled time.

Olaparib has been tested in other human cancers, as well as a standard range of safety pharmacology studies e.g. dog cardiovascular and respiratory function tests, and the rat Irwin test. There were no noticeable effects on the cardiovascular or respiratory parameters in the anaesthetized dog or any behavioural, autonomic or motor effects in the rat at the doses studied.
The toxicology studies indicate that the target organ of toxicity is the bone marrow.
Further information can be found in the current version of the Olaparib Investigator's Brochure.

Full details of Olaparib are fully described in the current version of the Olaparib Investigator's Brochure (IB), which has been provided in the site file.

It is not recommended for patients to consume grapefruit while on Olaparib therapy.

7.1 Dose interruptions and modifications

Induction chemotherapy

Dose modifications of induction chemotherapy should be according to local practice.

Olaparib/placebo

Any toxicity observed during the course of the study should first be assessed by the investigator and treated with supportive measures if deemed appropriate. If patients cannot be treated with supportive measures, toxicities will be managed by dose interruptions. Guidelines for specific toxicities are listed in section 7.2.

Investigators can choose to interrupt treatment for any grade of toxicity for a maximum of 14 days. The WCTU should be informed if the interruption is any longer than this. Olaparib/placebo must be interrupted until the patient recovers completely or when toxicity reverts to NCI CTCAE Grade 1 or less. If the toxicity resolves within the 14 days, then the patient should be dose reduced by one dose level. The patient should stop Olaparib/placebo for any interruption of more than 14 days (see below). The patient can have a maximum of 2 dose interruptions and therefore a maximum of two dose level reductions whilst on treatment.

Where toxicity re-occurs following re-challenge with Olaparib/placebo, and where further dose interruptions are considered inadequate for management of toxicity or there has been a maximum of 2 dose interruptions, then the patient must permanently discontinue treatment with Olaparib/placebo. Treatment must also be interrupted if any NCI-CTCAE grade 3 or 4 adverse event occurs which the investigator considers to be related to administration of Olaparib.

- Dose level 0 - 300mg bd
- Dose level 1 - 250mg bd
- Dose level 2 - 200mg bd

Dose escalations are not permitted.

All dose interruptions and modifications, and the reasons for these, are to be recorded in the CRF.

CONFIDENTIAL
This material is the property of the Wales Cancer Trials Unit. Do not disclose or use except as authorised.

7.2 Management of toxicities

Management of leukopenia and/or anaemia:

An exception to the management of Olaparib-related toxicity is the occurrence of leukopenia and/or anaemia. In this case, the AE should be managed as deemed appropriate by the investigator (eg G-CSF or blood transfusions). However, growth factors must be discontinued once the AE has recovered to CTCAE grade 1 or better. They may be resumed, if necessary, if leukopenia/anaemia develops again and discontinued once it recovers.

- Dose delay due to anaemia mandates a dose reduction. The dose should be reduced by one level (as above)
- Patients who develop grade 3 anaemia should be dose reduced by one level (as above)

Management of prolonged haematological toxicities including anaemia, neutropenia or thrombocytopenia whilst on study treatment:

If any study treatment is interrupted/delayed for a prolonged period because of one or more of the following, then <u>Olaparib/placebo should be discontinued</u>:

- ≥2 week interruption/delay in study treatment due to CTC grade >2 neutropenia
- ≥2 week interruption/delay in study treatment due to CTC grade >2 thrombocytopenia
- ≥2 week interruption/delay in study treatment due to CTC grade >2 anaemia and or development of blood transfusion dependence

Weekly blood counts including haemoglobin should be performed during the study treatment interruption/delay. If the levels have still not recovered to CTC Grade ≤1 after 4 weeks of dose interruption, the patient should be referred to a haematologist for further investigations. Bone marrow analysis or blood cytogenetic analysis should be considered at this stage according to standard haematological practice. Development of myelodysplastic syndrome or AML should be reported as an SAE and study drug should be discontinued. Full reports must be provided by the investigator to WCTU, for documentation on the Patient Safety database.

Management of new or worsening pulmonary symptoms:

If new or worsening pulmonary symptoms (e.g. dyspnoea) or radiological abnormality occurs, an interruption in Olaparib/placebo dosing is recommended and a diagnostic workup (including a high resolution CT scan) should be performed, to exclude pneumonitis. Following investigation, if no evidence of abnormality is observed on CT imaging and symptoms resolve, then Olaparib/placebo treatment can be restarted with a dose reduction, if deemed appropriate by the investigator. If significant pulmonary abnormalities are identified, these need to be discussed with the Chief Investigator, via the WCTU.

All dose reductions and interruptions (including any missed doses), and the reasons for the reductions/interruptions are to be recorded in the CRF.

Management in the case of surgery or palliative radiation:

Olaparib/placebo should be stopped before surgery and re-started after the wound has healed following recovery.
Olaparib/placebo should be discontinued for a minimum of 7 days before a patient undergoes therapeutic palliative radiation treatment. The patient may restart Olaparib/placebo following radiation treatment at the investigator's discretion.

7.3 Drug supply and distribution

Olaparib/placebo drug supply and distribution

AstraZeneca will supply IMP (Olaparib or matching placebo) to the investigator as follows:

Table 1 Identity of IMP

IMP	Dosage strength	Manufacturer
Olaparib	150 mg and 100mg	AstraZeneca
Placebo to match Olaparib	N/A	AstraZeneca

Olaparib/placebo tablets will be supplied in 32 count bottles.

All study drug supplied by AstraZeneca will be packaged and labelled by Fisher Clinical Services in accordance with local regulations and Good Manufacturing Practice, stating that the drug is for clinical trials use only and should be kept out of the reach of children. The drugs will be distributed by Fisher Clinical Services and supply will be managed by IWRS. The packaging and tablets will appear identical for both active and matching placebo treatments. The label attached to each package of blinded study material will have a unique treatment kit number that is linked to the randomisation scheme. Each centre will be provided with kits of packaged drugs. The IWRS will allocate the appropriate kit number from those available at the centre.

Patients enrolled in the study will be dispensed bottles of blinded Olaparib/placebo tablets as determined by the IWRS and randomisation scheme; each bottle will contain Olaparib 150mg or placebo tablets as determined by the randomisation scheme. Patients given dose reductions will be dispensed bottles of 100mg tablets as well as 150mg.

A detachable tear-off label will be affixed to each container and will contain space for centre number, patient number and date of dispensing to be completed and attached to the patient drug accountability CRF located in the pharmacy pack at the time of dispensing.

Patients will be supplied with sufficient medication for each visit. There will be sufficient tablets in the bottle to cover the visit window.

CONFIDENTIAL
This material is the property of the Wales Cancer Trials Unit. Do not disclose or use except as authorised.

7.4 Drug accountability

Storage

All IMPs must be kept in a secure place under appropriate storage conditions at room temperature under 30°C. A description of the appropriate storage and shipment conditions are specified on the labels. Study treatment must be kept out of the reach and sight of children.

Accountability

The medication provided for this study is for use only as directed in the protocol. Drug distribution and accountability logs will be provided to the site in a pharmacy pack. Pharmacies may be able to use their own logs but must request permission from WCTU, who will review their logs to ensure that they are compliant with the protocol. It is the investigator’s responsibility to establish a system for handling the IMP to ensure that:

- Deliveries of IMP from AstraZeneca via Fisher Clinical Services are correctly received by a responsible person (eg, pharmacist or suitable pharmacy designee), are handled and stored correctly and safely, and are documented on IMP accountability logs.
- IMP are dispensed only to study participants, and in accordance with the protocol.
- Participants return any unused IMP and all empty containers to the investigator.
- A dispensing record (which will include the identification of the participant to whom the IMP was dispensed, the date of dispensing, the quantity of IMP dispensed, and the date and quantity of any unused IMP returned to the pharmacy) is accurately maintained. Any discrepancies must be accounted for on the appropriate form. This record is in addition to any drug accountability information recorded in the CRF.

In the case that any study drug is damaged, please contact WCTU for reconciliation and replacement.

The participants will be given a diary card to keep track of the Olaparib/placebo tablets they have taken. This diary card is to assist hospital staff with CRF completion, and should not be returned to WCTU.

At the termination of the study or at the request of the sponsor, all unused drugs will be accounted for and destroyed locally at the study sites, with the prior permission of WCTU and in accordance with all applicable laws. Certificates of delivery and destruction or return must be signed and copies retained in the Investigator Site File.

CONFIDENTIAL
This material is the property of the Wales Cancer Trials Unit. Do not disclose or use except as authorised.

7.5 Concomitant medications

Any other treatment considered necessary for the patient's safety and well-being may be given at the discretion of the investigator(s), with the exception of the non-permitted medications listed below.

The administration of all medication (including IMPs) must be recorded in the appropriate sections of the case report form (CRF). Patients must be instructed that additional medication should not be taken without the prior consent of the investigator.

Permitted concomitant medications / procedures

Support medication is allowed according to standard local practice. Simple anti-emetics will be allowed.

Non-permitted concomitant medications / procedures

Potent inhibitors of CYP3A4 must not be used for any patient receiving Olaparib/placebo.

- Whilst this is not an exhaustive list, it covers the known potent inhibitors that have most often previously been reported to be associated with clinically significant drug interactions: Ketoconazole, erythromycin, itraconazole, ritonavir, indinavir, saquinavir, telithromycin, clarithromycin and nelfinavir (wash-out period 1 week).

- In addition, to avoid potential reductions in exposure due to drug interactions and, therefore, a potential reduction in efficacy, the following CYP3A4 inducers are excluded: Phenytoin, rifampicin, rifapentin, rifabutin, carbamazepine, phenobarbitone, nevirapine, modafinil and St John's Wort (wash-out period for phenobarbitone 5 weeks and for any of the others 3 weeks).

7.6 Code breaking procedure

A patient unblinding form will be available to investigators in the Cenduit IWRS system. Directions to use this form can be found in the Cenduit Study investigator and Site User Guide. The investigator and trials pharmacist (if applicable) will have access to unblind through this system in case of emergency. If IWRS is unavailable then the unblinding can be performed by the Cenduit Helpdesk by ringing Freephone 00 800 1012 1960. Prior to unblinding, Cenduit will confirm whether the caller has IWRS log in details, and if not then unblinding will not be able to be performed.

The treatment code must not be broken except in medical emergencies when the appropriate management of the patient necessitates knowledge of the treatment randomisation. If the treatment code is broken this will be automatically reported to the WCTU. Once unblinded, it will not be possible to dispense further IMP to the patient, and the treatment withdrawal form should be completed. The patient can continue in the trial follow-up.

8.0 Trial assessments

8.1 Stage 1 Registration baseline – Patients recruited before induction chemotherapy (inclusive of eligibility screening collected after patient consent):

- Informed consent
- Medical history
- CT scan (standard pre-treatment)
- Inclusion and exclusion criteria check
- Translational blood sample
- Collection of diagnostic tumour tissue biopsy.

8.2 **Chemotherapy details – All patients**

- Chemotherapy treatment details

8.3 Pre-Randomisation screening procedures

The following procedures should be performed between day 1 of final induction chemotherapy cycle and randomisation. There must be no more than 42 days between day 1 of last cycle of chemotherapy and starting olaparib

- CT scan and RECIST assessment

Within 14 days prior to randomisation

- Medical history (if not already registered in Stage 1)
- Pregnancy test
- Physical examination including vital signs and ECOG status
- Weight and height
- Blood pressure, pulse, oxygen saturation
- Urinalysis (pH, glucose, blood, protein and leucocytes)
- Full blood count
- Serum biochemistry (renal, liver and bone profiles)
- Documentation of concomitant medication
- Electrocardiogram (ECG)
- Assessment of inclusion and exclusion criteria
- Evaluation of toxicities using NCI CTCAE (v4.03) and real-time serious adverse event (SAE) reporting
- Translational bloods
- Collection of diagnostic tumour tissue biopsy (if not already collected in stage 1) 14 day time limit doesn't apply, but biopsy must be available.

8.4 During Maintenance Therapy (every 3 weeks) until disease progression, or up to 1 year post randomisation

- Documentation of dose delays and/or reduction

This material is the property of the Wales Cancer Trials Unit. Do not disclose or use except as authorised.

- CT scan to be conducted every six weeks until disease progression with full clinical examination to evaluate disease response by RECIST (v1.1) criteria
- Physical examination
- ECOG performance status
- Weight
- Blood pressure, pulse, oxygen saturation
- Urine dipstick (pH, glucose, blood, protein and leucocytes).
- Full blood count (within 72 hours prior to dispensing of new Olaparib/placebo prescription)
- Serum biochemistry (renal, liver and bone profiles) (within 72 hours prior to dispensing of new Olaparib/placebo prescription)
- Evaluation of toxicities using NCI CTCAE (v4.03) and real-time SAE reporting
- Documentation of concomitant medication
- Electrocardiogram (ECG) (**9 weeks after starting treatment**).

8.5 Upon radiological disease progression, or up to 1 year post randomisation

- Translational blood sample
- Tumour tissue biopsy (optional)

8.6 30 days post-treatment visit

- Physical examination
- ECOG performance status
- Weight
- Blood pressure, pulse, oxygen saturation
- Electrocardiogram (ECG)
- Evaluation of toxicities using NCI CTCAE (v4.03) and real-time SAE reporting

If the registered participants do not meet the criteria for randomisation then they will be treated according to local standard clinical practice. RECIST data will be collected on disease status following induction chemotherapy. Only participants who progress following/during induction chemotherapy will be asked to consent to provide a follow-up translational blood sample.

8.7 Completion of CRFs

All source data recorded in the CRFs must be evidenced in the patient notes.

A full set of instructions for completing the CRFs are included on the inside cover of each CRF pack. CRFs will be printed on NCR (carbonless copy) paper and should therefore be completed in black ball point pen, with participant trial number, initials and date of birth recorded on the header of every page. Incorrectly entered information can only be amended on the top copy of the CRF if it has not already been separated from the NCR copy underneath. Delete with a single line through the entry and write the correct value alongside the box, all amendments should be initialled and dated. The top copy of completed CRFs should be torn out and sent in the post to the WCTU within four weeks unless stated otherwise.

CONFIDENTIAL
This material is the property of the Wales Cancer Trials Unit. Do not disclose or use except as authorised.

8.8 Schedule of trial assessments

**Assessments**	**Stage 1 entry (optional)**	**Post induction chemotherapy**	**Stage 2 entry (randomisation)**	**Day -4 to 1 of each 21 day cycle**	**Daily**	**Upon disease progression**	**30 day post treatment visit**
Informed consent	X		X				
Medical history	X	X[6]					
Registration	X	X[6]					
CT scan	X	X		X[1]			
RECIST Disease evaluation		X		X[2]			
Co-morbidities check	X						
Pregnancy test			X				
Physical exam			X	X			X
Vital signs			X	X			X
ECOG performance status			X	X			X
Urine Dipstick			X	X			
Serum biochemistry			X	X			
Peripheral blood smear[3]			X				
Full blood count			X	X			
ECG			X	X[4]			X
Inclusion / exclusion criteria	X		X				
Randomisation			X				
Toxicity Assessment			X	X			X
Concomitant medication			X	X			
**Treatments**							
Chemotherapy		X					
Olaparib / placebo				X	X		
Dose delays or reductions				X			

[1]&[2] CT scan and RECIST assessment every 2 cycles only – scan should be performed in the 7 days before the next cycle of treatment
[3] To be done if blood count/morphology suggestive of MDS/AML
[4] To be taken at 9 week visit only
[5] Only taken from Stage 1 participants if they have disease response or disease progression. A blood sample from Stage 1 participants with stable disease is not required.
[6] If not already registered to stage 1 of the trial
[7] Archival tumour biopsy
[8] Optional tumour biopsy sample on progression

CONFIDENTIAL
This material is the property of the Wales Cancer Trials Unit. Do not disclose or use except as authorised.

	Stage 1 entry (optional)	Post induction chemotherapy	Stage 2 entry (randomisation)	Day -4 to 1 of each 21 day cycle	Daily	Upon disease progression	30 day post treatment visit
**Translational Samples**							
Translational blood	X	X[5]	X[6]			X	
Tumour biopsy sample	X[7]		X (diagnostic)[6]			X[8]	

9.0 Safety reporting and pharmacovigilance

The Principal Investigator is responsible for ensuring that all site staff involved in this trial are familiar with the content of this section.

The following definitions are in accordance with the Medicines for Human Use (Clinical Trials) Regulations 2004 (SI2004/1031) (as amended) and EU Directive 2001/20/EC.

**Term**	**Definition**
**Adverse Event (AE)**	Any untoward medical occurrence in a patient or clinical trial participant administered a medicinal product and which does not necessarily have a causal relationship with this treatment. An adverse event can therefore be any unfavourable and unintended sign (including an abnormal laboratory finding for example), symptom or disease temporally associated with the use of a medicinal product, whether or not considered related to the medicinal product.
**Adverse Reaction (AR)**	Any untoward and unintended response in a clinical trial participant to an investigational medicinal product which is related to any dose administered to that participant
**Serious Adverse Event (SAE)**	Any adverse event that -<br>• Results in death<br>• Is life-threatening*<br>• Required hospitalisation or prolongation of existing hospitalisation**<br>• Results in persistent or significant disability or incapacity<br>• Consists of a congenital anomaly or birth defect<br>• Other medically important condition ***<br>In addition for the purposes of this trial the following events will also be considered SAEs and must be captured on the SAE form and reported to the WCTU with 24hours of knowledge of the event:<br>• MDS or AML<br>For the purposes of this trial the following events will not

	require immediate reporting<br>• SAEs during induction chemotherapy<br>• Death due to disease progression<br>These should be completed in the participants notes and on the relevant toxicities CRF page and forwarded to the WCTU in the normal timeframes for CRFs.
**Serious Adverse Reactions (SARs)**	Any SAE occurring in a clinical trial participant for which there is a reasonable possibility that it is **related** to the IMP at any dose administered.
**Suspected Unexpected Serious Adverse Reactions (SUSARs)**	A SAR, the nature and severity of which is not consistent with the Reference Safety Information (RSI) for the IMP.

***Note:** The term 'life-threatening' in the definition of serious refers to an event in which the trial participant was at risk of death at the time of the event or it is suspected that used or continued used of the product would result in the subjects death; it does not refer to an event which hypothetically might have caused death if it were more severe.

**** Note:** Hospitalisation is defined as an inpatient admission, regardless of the length of stay, even if the hospitalisation is a precautionary measure for continued observation. Pre-planned hospitalisation e.g. for pre-existing conditions which have not worsened, or elective procedures, does not constitute an SAE.

***** Note:** other events that may not result in death, are not life-threatening, or do not require hospitalisation, may be considered as an SAE when, based upon appropriate medical judgement, the event may jeopardise the participant and may require medical or surgical intervention to prevent one of the outcomes listed above.

9.1 Causality Assessments

The Principal Investigator (or another delegated medically qualified doctor from the trial team) and Chief Investigator (or another medically qualified doctor from the Trial Management Group) will assess each SAE to determine the causal relationship with the IMP, and will answer 'yes' or 'no' to the question "Do you consider that there is a reasonable possibility that the SAE may have been caused by the IMP?"

For SAEs causal relationship will also be assessed for other trial treatments (nIMPs) and procedures.

**IMPs:** Olaparib<br>**nIMPs:** Induction chemotherapy as per local practice

The causality assessment given by the Principal Investigator (or delegate) cannot be downgraded by the Chief Investigator (or delegate), and in the case of disagreement both opinions will be provided.

This material is the property of the Wales Cancer Trials Unit. Do not disclose or use except as authorised.

A guide to the interpretation of the causality question is found in Appendix A of this clinical trial protocol.

9.2 Expectedness Assessments

The Chief Investigator (or another delegated appropriately qualified individual) will assess each SAE to perform the assessment of expectedness.

The expectedness assessment should be made with reference to the current Reference Safety Information (RSI). Expectedness decisions must be based purely on the content of the RSI; other factors such as the participant population and participant history should not be taken into account. Expectedness is not related to what is an anticipated event within a particular disease.

SAEs which add significant information on specificity or severity of a known, already documented adverse event constitute unexpected events. For example, an event more specific or more severe than that described in the RSI is considered unexpected.

The table below lists the RSI's that should be referenced

IMP	Most recent version of Olaparib Investigator's Brochure	Section 5.4

9.3 SAE reporting

9.3.1 Participating Site Responsibilities

All SAEs must be reported immediately (and within 24 hours of knowledge of the event) by the PI at the participating site to the WCTU unless the SAE is specified as not requiring immediate reporting (see above). This includes SAEs related to IMPs and non-Investigational Medicinal Products (nIMPs).

The PI (or delegated medically qualified doctor from the trial team) should sign and date the SAE CRF to acknowledge that he/she has performed the seriousness and causality assessments.

A completed SAE form for all events requiring immediate reporting should be **emailed** to the WCTU within 24 hours of knowledge of the event. All SAEs should be reported using email and we strongly encourage you to use this method. However, where sending SAE forms by email is not possible, please use the SAE fax service. This should only be used if absolutely necessary. A separate form must be used to report each event, irrespective of whether or not the events had the same date of onset.

The participant will be identified only by trial number, date of birth and initials. The participant's name should not be used on any correspondence.

It is also required that sites respond to and clarify any queries raised on any reported SAEs and report any additional information as and when it becomes available through to the resolution of the event.

Serious Adverse Event (SAE) Email:
CTR-Safety@cardiff.ac.uk

SAE Fax number:
0203 0432 376

Serious adverse events should be reported from time of signature of informed consent, throughout the treatment period up to, and including 30 days after the participant receives their last dose of the IMP. Serious adverse reactions (such as long term side effects of trial treatment under investigation) should continue to be reported until the end of follow up.

Adverse events (AE) should be graded using the NCI Common Terminology Criteria for Adverse Events (CTCAE) Version 4.03. The toxicity grades should be recorded on the toxicity part of the CRF.

An SAE form is not considered as complete unless the following details are provided:

- Full participant trial number
- An Adverse Event / Adverse Reaction
- A completed assessment of the seriousness, and causality as performed by the PI (or another appropriately medically qualified doctor registered on the delegation log).

If any of these details are missing, the site will be contacted and the information must be provided by the site to the WCTU within 24 hours.

All other AEs should be reported on the CRF following the CRF procedure described in Section 8.7.

9.3.2 The WCTU responsibilities

Following the initial report, all SAEs should be followed up to resolution wherever possible, and further information may be requested by the WCTU.

The WCTU should continue reporting SAEs until 30 days after the participant receives their last dose of the investigational medicinal product. Serious adverse reactions should continue to be reported until the end of follow up.

Once an SAE is received at the WCTU, it will be evaluated by staff at the WCTU and sent to the Chief Investigator (or their delegate) for an assessment of causality and expectedness.

Investigator reports of suspected SARs will be reviewed immediately and those that are identified as SUSARs are reported to the MHRA, Main Ethics Committee and AstraZeneca.

9.4 SUSAR reporting

Velindre NHS Trust is undertaking the duties of trial Sponsor and has delegated to the WCTU the responsibility for reporting SUSARs and other SARs to the regulatory authorities (MHRA and relevant ethics committees) and to AstraZeneca as follows:

CONFIDENTIAL
This material is the property of the Wales Cancer Trials Unit. Do not disclose or use except as authorised.

SUSARs which are fatal or life-threatening must be reported to the MHRA and MREC within 7 calendar days of receipt at the WCTU. Any additional, relevant information must be reported within a further 8 calendar days of submitting the initial report.

SUSARs that are not fatal or life-threatening must be reported to the MHRA and MREC within 15 days of receipt at the WCTU. Any additional, relevant information must be reported within a further 15 days.

N.B. There is no requirement for WCTU to report SUSARs to nIMPs to the MHRA except in the following instances:

- If the adverse reaction is suspected to be linked to an interaction between a nIMP and IMP, and is serious and unexpected, WCTU should report as a SUSAR due to the interaction with the IMP.
- If a SUSAR is suspected and might be linked to either a nIMP or an IMP and cannot be attributed to only one of these.
- If the adverse reaction due to the nIMP is likely to affect the safety of trial subjects then WCTU should report it to the MHRA and Main Ethics Committee in accordance with the relevant Standard Operating Procedure for reporting Urgent Safety Measures.

9.5 Unblinding for the purposes of SUSAR reporting

To enable processing of a SAR in this blinded trial, expectedness should be assessed initially using the assumption that the IMP has been given to the trial participant.

If it is assessed as unexpected as per the RSI of the IMP the SUSAR will be unblinded by the WCTU safety group prior to reporting to the MHRA and MREC.

If after unblinding it is evident that the trial participant received the placebo, this event will not require expedited reporting to the MHRA and MREC, unless in the opinion of the Principal Investigator or Chief Investigator the event was related to the placebo (for example an allergic reaction to an excipient).

9.6 Safety Reports

A list of all SARs (expected and unexpected) will be reported annually to the MHRA, Main Ethics Committee, trial sponsor and AstraZeneca in a Development Safety Update Report (DSUR). This report must be submitted within 60 days of the anniversary of the MHRA CTA approval date.

The WCTU will report a list of all SARs (expected and unexpected) and any other safety recommendations to all PIs six monthly throughout the course of the trial. This frequency may be reviewed and amended as necessary. This reporting will be done via the Investigator safety report (ISR).

The WCTU will notify AstraZeneca of any SAEs, SARs and SUSARs within 24 hours of becoming aware of the event. AstraZeneca will be sent copies of any submissions regarding SARs and SUSARs that are sent to the competent authorities.

CONFIDENTIAL
This material is the property of the Wales Cancer Trials Unit. Do not disclose or use except as authorised.

9.7 Pregnancy reporting whilst participating in the PIN trial

Pregnancy, or the pregnancy of a partner occurring whilst participating in PIN trial, although not considered an SAE, must be notified to the WCTU within the same timelines as an SAE (i.e. during the trial treatment period and up to 30 days after the last date of treatment).

In the event of a pregnancy, if the participant or the female partner of a male participant have read the Pregnancy Information Sheet, and signed the Pregnancy Consent Form, the WCTU must be contacted immediately to request a Pregnancy Report Form. The Pregnancy Report Form should be completed and returned to the WCTU to capture all the relevant information required for the expedited reporting of these events.

The outcome of a pregnancy should be followed up carefully and any abnormal outcome of the mother or the foetus should be reported. This also applies to pregnancies following the administration of the IMP to the father prior to sexual intercourse.

A congenital anomaly or birth defect is considered an SAE and should be reported to the WCTU within 24 hours of knowledge of the event.

CONFIDENTIAL
This material is the property of the Wales Cancer Trials Unit. Do not disclose or use except as authorised.

9.8 Flowchart for Serious Adverse Event reporting

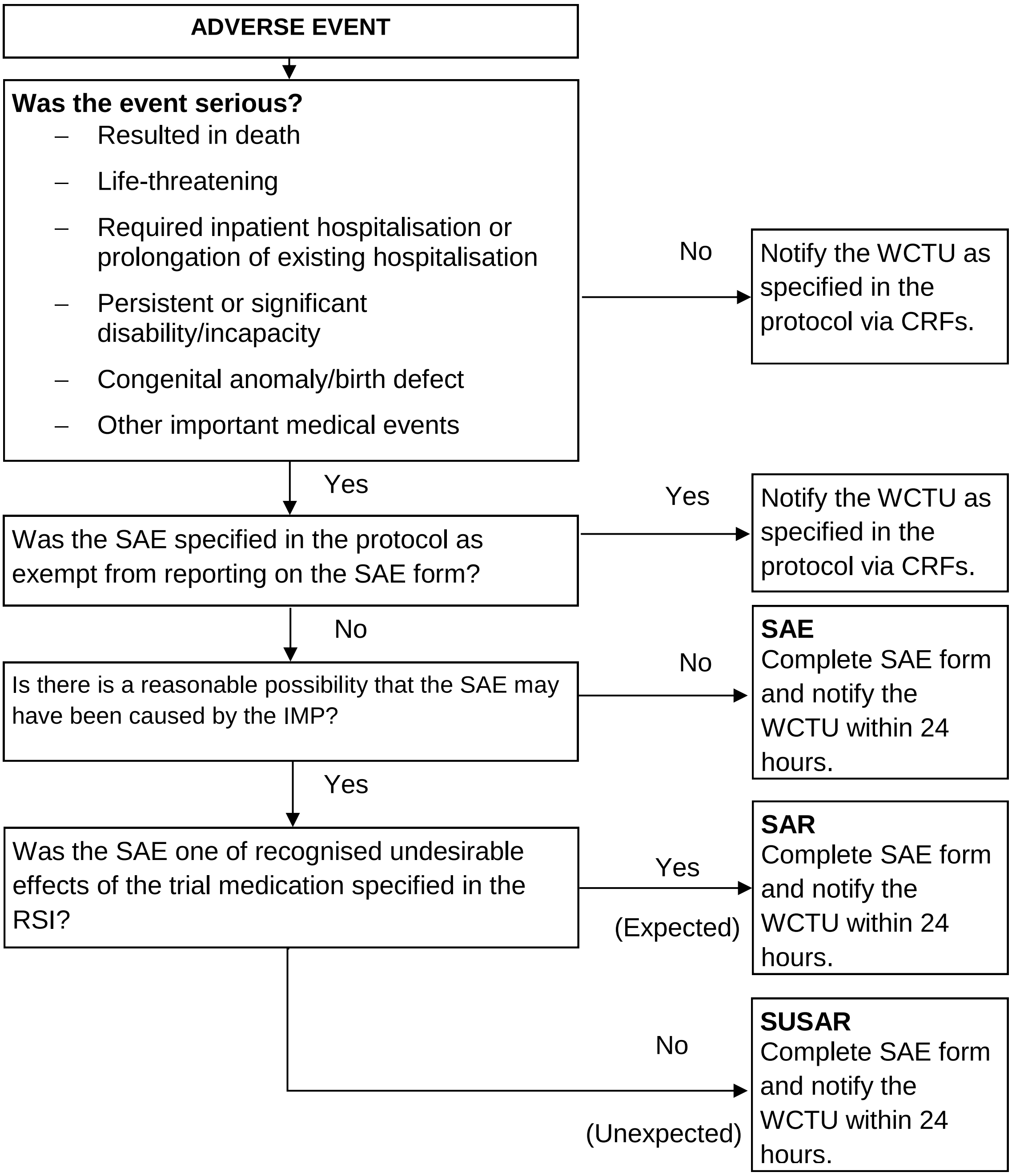

CRF Case Report Form
RSI Reference Safety Information
SAE Serious Adverse Event
SAR Serious Adverse Reaction
SUSAR Suspected Unexpected Serious Adverse Reaction
WCTU Wales Cancer Trials Unit

CONFIDENTIAL
This material is the property of the Wales Cancer Trials Unit. Do not disclose or use except as authorised.

10.0 Trial management

10.1 Trial committees and trial management

The conduct of the trial is being overseen by the following committees:

1. Trial Management Group (TMG): The TMG will be responsible for the day-to-day running of the trial and will meet at least once every six months. The TMG members will include the Chief Investigator, other active trial investigators, WCTU representatives, and specialist advisors (e.g. Pharmacist, Statistician, consumer representative).

2. Independent Data Monitoring Committee (IDMC): The IDMC will consist of at least two independent Clinicians (not entering patients into the trial) and an independent Statistician who will review trial data approximately every six months. The remit of the IDMC will be to recommend whether the accumulated data from the trial, together with results from other relevant trials, justifies continuing recruitment of further patients. A decision to recommend discontinuation of recruitment, in all patients or in selected subgroups, will be made only if the result is likely to convince a broad range of Clinicians including PIs in the trial and the general clinical community. If a decision is made to recommend continuation, the IDMC will advise on the frequency of future reviews of the data on the basis of accrual and event rates. There will be two initial safety reviews of toxicities and SAEs after 10 and 20 participants on each arm have completed trial treatment. Randomisation will continue during the safety review process. The IDMC will make confidential recommendations to the Trial Steering Committee (TSC).

3. Independent Trial Steering Committee (TSC): The TSC will be a committee of independent members that provides overall supervision of the trial. The role of the TSC is to act on behalf of the sponsor, to provide overall supervision for the trial, to ensure that it is conducted in accordance with GCP, and to provide advice through its independent chairman. The TSC will review the recommendations from the IDMC and will decide on continuing or stopping the trial, or modifying the protocol. It will meet at least annually when it will consider each report of the IDMC, as well as results of other trials and new information which has arisen, and recommend appropriate action.

10.2 Monitoring

10.2.1 Site monitoring

Investigators should agree to allow trial related monitoring, including audits and regulatory inspections, by providing direct access to source data/documents as required. Participant consent for this will be obtained.

Routine and triggered monitoring visits will be performed, the frequency of which will be detailed in the separate trial monitoring plan.

CONFIDENTIAL
This material is the property of the Wales Cancer Trials Unit. Do not disclose or use except as authorised.

10.2.2 Central monitoring and data queries

The top copy of each completed CRF should be returned to the WCTU for data entry within four weeks of the visit. The remaining copy is to be retained at the local centre.

CRF pages and data received by the WCTU from participating trial centres will be checked for missing, illegible or unusual values (range checks) and consistency over time.

If missing or questionable data are identified, a data query will be raised on a data clarification form. The data clarification form will be sent to the relevant participating site. The site shall be requested to answer the data query or correct data on the data clarification form. **The case report form pages should not be altered.**

All answered data queries and corrections should be signed off and dated by a member of staff listed on the trial delegation log at the relevant participating site. The completed data clarification form should be returned to the WCTU and a copy retained at the site along with the participants' CRFs.

The WCTU shall send reminders for any overdue data. It is a centre's responsibility to submit complete and accurate data in a timely manner

10.3 Participant withdrawal

In consenting to the trial, participants are consenting to trial treatment, trial follow-up and data collection. Participants may withdraw from the trial at any time. Patients may:

Level 1: Withdraw from trial treatment – participants stop trial treatment but remain in follow-up and continue to provide translational samples if applicable.

Level 2: Withdraw from the translational study – participants continue trial treatment and follow-up but do not provide translational samples

Level 3: Withdrawal from the translational study and trial treatment - participants stop trial treatment and do not continue providing translational samples but remain in follow-up.

Level 4: Complete withdrawal from the trial – participants stop trial treatment, follow-up and any translational sample collection.

If a participant wishes to withdraw from trial treatment, participating sites should nevertheless explain the importance of remaining on trial follow up for the purposes of data capture only. Withdrawal for any reason requires a completed withdrawal CRF to be faxed to the WCTU with the hard copy to follow soon after. Participants do not have to give a reason for their withdrawal but sites should make a reasonable attempt to find out why.

A participant may withdraw, or be withdrawn, from trial treatment for the following reasons:

- Voluntary discontinuation by the participant who is at any time free to discontinue study treatment or their participation in the study as a whole, without prejudice to further treatment

This material is the property of the Wales Cancer Trials Unit. Do not disclose or use except as authorised.

- Safety reasons as judged by the investigator and/or sponsor
- Severe non-compliance to protocol as judged by the investigator and/or sponsor
- Participant lost to follow-up
- Clinical disease progression
- Development of MDS or AML
- Incorrect enrolment i.e., the participant does not meet the required inclusion/exclusion criteria for the study

Data and samples collected prior to participant withdrawal at any of the four levels indicated above will be collected and used for trial analysis by the WCTU. Participants who initially consented to be registered with the National Health Service Information Centre (NHSIC) or equivalent will remain on the system so that important research information on date and cause of death can be requested from NHSIC by the WCTU.

10.4 Lost to follow up

If a participant is lost to follow up the WCTU will contact the participant's GP to obtain information on the participant's status. We will obtain additional consent from participants to register their details with the NHS Information Centre to obtain confirmation of date and cause of death.

10.5 Protocol/GCP non-compliance

The Principal Investigator should report any non-compliance to the trial protocol or the conditions and principles of GCP to the WCTU using the WCTU Protocol/GCP non-compliance proforma as soon as they become aware of it.

10.6 The End of the Trial

For the purposes of both MHRA and Research Ethics Committee approval, the study end date is deemed to be the date of last data capture.
The Trial Master File (TMF) and trial data will be archived by the WCTU for a minimum period of 15 years.

10.7 Archiving

The TMF and Investigator Site File (ISF) containing essential documents will be archived at an approved external storage facility for a minimum of 15 years. The WCTU will archive the TMF and Trial Site Files (TSFs) on behalf of the Sponsor. The Principal Investigator is responsible for archival of the ISF at site. Essential documents pertaining to the trial shall not be destroyed without permission from the Sponsor.

CONFIDENTIAL
This material is the property of the Wales Cancer Trials Unit. Do not disclose or use except as authorised.

11.0 Statistical considerations

11.1 Randomisation

Randomisation will take place centrally via the interactive web response system (IWRS) after confirmation of eligibility. Participants will be randomised using stratified permuted blocks. This will ensure balanced treatment allocation by a number of clinically important stratification factors. Randomisation will have an allocation ratio of 1:1.

11.2 Outcome measures

11.2.1 Primary outcome measure

- To establish the anti-tumour activity of Olaparib as measured by progression-free survival (PFS). This is the time from randomisation to any disease progression and/or any death, defined according to strict RECIST v1.1 (www.recist.com) criteria. Lesions will be compared to baseline measurements to assess progression.

11.2.2 Secondary outcome measures

- Tolerability (side effects) and feasibility of use (number of participants requiring dose delays or reductions and/or treatment withdrawal).
- Objective response rate as assessed by RECIST criteria
- Overall survival (OS). Time from randomisation to death. Those still alive will be censored at time last seen.
- To measure anti-tumour activity in terms of tumour volume.
- To collect tumour and blood samples for translational work including investigating BRCA1 expression

11.3 Sample size calculation

The median PFS for patients with CR/PR after 4-6 cycles is expected to be 3 months. With 80% power and a one-sided α (type I error) of 0.2, 68 participants are required in total to demonstrate statistical significance between the arms if the true hazard ratio for Olaparib compared to placebo is 0.65, based on the logrank test. We therefore aim to recruit 34 participants into each arm, over a 12 month accrual period, with minimum participant follow-up of at least 6 months prior to disease progression, making the total trial length 1.5 years. The primary analysis of data will be analysed after 63 PFS events.

11.4 Statistical analyses

A full statistical analysis plan will be developed before the first analysis of the trial and will be signed off prior to unblinding the data for analysis.

A WCTU statistician will unblind the data prior to analyses for the safety run-in, IDMC analysis and final report. Unblinded interim reports will only be seen by the WCTU statistician and the IDMC. The final clinical study report will be unblinded.

CONFIDENTIAL
This material is the property of the Wales Cancer Trials Unit. Do not disclose or use except as authorised.

Safety will be assessed by the IDMC when 10 and 20 patients on each arm have completed trial treatment.

The trial data will be analysed when all participants have completed a minimum 6 months follow up and at least 63 disease progression events are observed. Disease progression will be formally assessed according to the RECIST v1.1 criteria.

At the end of the trial both an intention-to-treat (all results analysed according to the participants' original trial arm allocation) and a per protocol analysis will be carried out. We will attempt to trace participants who are lost to follow-up via their GP or through the NHS-IC. Where no information is available these participants will be censored at the date last seen.

Primary analysis will be of Progression-Free Survival described using Kaplan-Meier curves in both arms of the trial. The median PFS will be calculated for each arm of the trial, and then the logrank test will be used to formally test the equality of the survivor functions. If the hazards are proportional, then Cox regression will also be performed to adjust the hazard ratio for the stratification factors.

Secondary analysis will include:

- Toxicity data will be summarised, serious adverse events will be listed and toxicities will be presented in tabular format. Tolerability and feasibility of use will be summarised in terms of the number of treatment reductions, delays and treatment withdrawals.
- The objective response rate will be summarised, based on Radiological disease progression defined according to RECIST (Response Evaluation Criteria In Solid Tumours) v1.1. The proportions of participants in each objective disease response (RECIST v1.1) category will be reported at each CT scan.
- Overall survival (OS) will be calculated from the time of randomisation to date of death or date censored (date last known to be alive). OS will be presented as median time-to-event and with Kaplan-Meier curves.
- The change of size of measurable lesions at each disease assessment after start of chemotherapy will be assessed using Waterfall plots.

11.5 Subgroup analyses

Translational data will be used to perform subgroup analyses as described in section 12.0.

Additional exploratory analyses may be conducted to inform the design of a future phase III trial.

CONFIDENTIAL
This material is the property of the Wales Cancer Trials Unit. Do not disclose or use except as authorised.

12.0 PIN Translational research

Baseline translational blood samples will be collected from all participants entering Stage 1 before induction chemotherapy starts. Follow-up blood samples will be collected upon disease progression following induction chemotherapy, randomisation (for those patients who go on to be randomised) and disease progression following randomisation. Follow-up blood samples will not be collected from patients with stable disease at the end of induction chemotherapy. We request that all centres have the facilities to spin and store blood samples. Stored blood will be collected on dry ice and sent to the Wales Cancer Bank at the end of study. Details of dry ice collections can be found in the Translational Pack.

Using ultra-deep re-sequencing (6000x) of plasma, we are able to detect around 700 mutations corresponding to 46 cancer genes in the blood of patients with advanced NSCLC. This is using the Ion Torrent/Ampliseq cancer panel platform. Plasma collected at baseline, randomisation and disease progression will be sequenced for these mutations, to determine whether specific somatic gene alterations in DNA repair pathways correlate with enhanced sensitivity to both platinum based chemotherapy and PARP inhibition.

Formalin fixed paraffin embedded, archival diagnostic NSCLC tissue will be collected on all patients enrolled into this clinical trial either at the time of induction chemotherapy or upon their entry to Stage 2. Patients who have reached the randomisation stage will also be asked to donate an optional tissue sample upon progression. Consent for this will be sought at time of registration. Tissue will be batched and transferred to the Wales Cancer Bank at the end of study.

This PIN trial biorepository will comprise not only chemoresponsive but also chemorefractory NSCLCs. Tissue will be used to assess for putative predictive biomarkers associated with synthetic lethality in the context of PARP inhibition. For example, it is anticipated that loss of BRCA1 expression will be enriched in chemoresponsive NSCLCs. BRCA1 negativity will be explored as a stratification factor for future trials. A project grant currently funded by the British Lung Foundation is exploring potential causes of resistance to Olaparib in NSCLC and may generate hypotheses for exploratory analysis. A specific translational research grant will be sought to enable exploratory analysis.

13.0 Publication policy

Data from all centres will be analysed together and published as soon as possible. Individual participating PIs may not publish data concerning their participants that are directly relevant to questions posed by the trial until the TMG has published its report. The TMG will form the basis of the writing committee and will advise on the nature of publications, subject to sponsor requirements.

All publications should include a list of participating PIs, and if there are named authors, these should include the Chief Investigator, Co-Investigators, Trial Manager, and Statistician(s) involved in the trial. If there are no named authors then a writing committee will be identified.

CONFIDENTIAL
This material is the property of the Wales Cancer Trials Unit. Do not disclose or use except as authorised.

14.0 Informed consent, ethical and regulatory considerations

14.1 Ethical approval

This protocol will be submitted to a Multi-centre Research Ethics Committee (MREC) that is legally "recognised" by the United Kingdom Ethics Committee Authority for review and approval. The approval of the MREC must be obtained before the start of a clinical trial or any trial procedures are conducted.

14.2 Clinical Trial Authorisation (CTA)

The trial is being performed under a Clinical Trial Authorisation (CTA) from the MHRA. The Clinical Trials Authorisation (CTA), the approval of the MHRA, must be obtained before the start of the trial in accordance with Part 3, Regulation 12 of The Medicines for Human Use (Clinical Trials) Regulations 2004 (SI2004/1031).

14.3 Regulatory considerations

All substantial amendments to this Protocol must be approved by the MREC responsible for the study and MHRA, before the implementation of the amendments. Minor amendments will not require prior approval by the MREC and MHRA.

If the trial is temporarily halted it will not be recommenced without reference to the MREC responsible for the study and the MHRA.

The MREC and MHRA will be notified within 90 days of trial completion. If the trial is terminated early, the MREC and MHRA will be notified of this within 15 days.

A summary of the clinical trial report will be submitted to the MREC responsible for the study and MHRA within one year of the end of trial.

14.4 Research governance approval

This trial protocol will be submitted through the Research Governance process of the host care organisation for review and approval. The Research Governance approval of the host care organisation must be obtained before recruitment of participants within that host care organisation.

14.5 Sponsorship

The PIN trial is being sponsored by Velindre NHS Trust. Velindre NHS Trust shall be responsible for ensuring that the trial is performed in accordance with the following:

- The Medicines for Human Use (Clinical Trials) Regulations 2004 (SI2004/1031) and subsequent amendments
- Conditions and principles of Good Clinical Practice
- Declaration of Helsinki (1996)

This material is the property of the Wales Cancer Trials Unit. Do not disclose or use except as authorised.

- Research Governance Framework for Health and Social Care (Welsh Assembly Government 2009 and Department of Health 2nd July 2005),
- the Data Protection Act 1998,
- the Human Tissue Act 2004
- other regulatory requirements as appropriate

The Sponsor has/will be delegating certain responsibilities to Cardiff University (WCTU), the Chief Investigators, Principal Investigators, host sites and other stakeholder organisations as appropriate in accordance with the relevant agreement that is informed by regulation and study type.

14.5 Indemnity

- Non-negligent harm: This trial is an academic, investigator-led and designed trial sponsored by Velindre NHS Trust and coordinated by the WCTU. The Chief Investigator, local Investigators and WCTU do not hold insurance against claims for compensation for injury caused by participation in a clinical trial and therefore cannot offer any non-negligent harm indemnity. The Association of the British Pharmaceutical Industry (ABPI) guidelines will not apply.
- Negligent harm: In accordance with Technical Note 12 Indemnity for Clinical Research for research Sponsored by a Welsh body, Welsh Risk Pool Services provides indemnity cover against successful negligence claims arising from the management and conduct of the study. Where NHS employees are responsible for the design of a study, indemnity cover will also be provided for negligent harm arising from the study design. Velindre NHS Trust does not accept liability for any breach in the other NHS Organisations duty of care, or any negligence on the part of employees of these NHS Organisations.

14.6 Data protection

The WCTU will act to preserve patient confidentiality and will not disclose or reproduce any information by which participants could be identified (except where participants are registered with the National Health Service Information Centre (formerly the Office for National Statistics) or traced via the NHS Central Register or equivalent for Scotland and Northern Ireland, which requires separate consent. Consent will also be obtained from trial participants to have their NHS numbers provided to CR-UK). Data will be stored in a secure manner and WCTU trials are registered in accordance with the Data Protection Act 1998. The data custodian for this trial is the Director of the WCTU. The custodian of the translational samples is the CI.

14.7 Finance

PIN is part funded by Cancer Research UK (CR-UK) Feasibility Study Committee (FSC) (funder's no: CRUK/11/055). The trial is supported and part funded by an Investigator-Sponsored Study Collaboration between AstraZeneca and the National Cancer Research Network. The WCTU is core funded by CR-UK and these core resources will be used to support this trial. The trial is in the National Cancer Research Network (NCRN) and National Institute for Health (NIHR) portfolio. Local

CONFIDENTIAL
This material is the property of the Wales Cancer Trials Unit. Do not disclose or use except as authorised.

NCRN/WCRN/SCRN support should be available at each centre taking part to support entry of participants into this trial.

15.0 References

1. Cancer Research UK: www.cancerresearchuk.org/cancer- info/cancerstats/types/lung/mortality

2. Schiller, J.H. et al., *Comparison of four chemotherapy regimens for advanced non-small cell lung cancer*. N Eng J Med, 2002. **346**(2): p.92-98.

3. Lara, P.N. et al., *Disease control rate at 8 weeks predicts clinical benefit in advanced non-small cell lung cancer: results from Southwest Oncology Group randomized trials*. J Clin Oncol, 2008. **26**(3): p.463-467.

4. Scagliotti, G.V. et al., *Phase III study comparing cisplatin plus gemcitabine with cisplatin plus pemetrexed in chemotherapy-naïve patients with advanced-stage non-small cell lung cancer*. J Clin Oncol, 2008. **26**(21): p. 3543-51.

5. Ceppi, P. et al., *Squamous cell carcinoma of the lung compared with other histotypes shows higher messenger RNA and protein levels for thymidylate synthase*. Cancer, 2006. **107**(7): p.1589-96.

6. Paez, J.G. et al., *EGFR mutations in lung cancer: correlation with clinical response to gefitinib therapy*. Science, 2004. **304**(5676): p.1497-500.

7. Lynch, T.J. et al., *Activating mutations in the epidermal growth factor receptor underlying responsiveness of non-small cell lung cancer to gefitinib*. N Engl J Med, 2004. **350**(21): p.2129-39.

8. Deng, J. et al., *Proapoptotic BH3-only BCL-2 family protein BIM connects death signalling from epidermal growth factor receptor inhibition to the mitochondrion*. Cancer Res, 2007. **67**(24): p.11867-75.

9. Cragg, M.S. et al., *Gefitinib-induced killing of NSCLC cell lines expressing mutant EGFR requires BIM and can be enhanced by BH3 mimetics*. PLoS Med, 2007. **4**(10): p.1681-89; discussion 1690.

10. Costa, D.B. et al., *BIM mediates EGFR tyrosine kinase inhibitor-induced apoptosis in lung cancers with oncogenic EGFR mutations*. PLoS Med, 2007. **4**(10): p.1669-79; discussion 1680.

11. Mok, T.S. et al., *Gefitinib or carboplatin-paclitaxel in pulmonary adenocarcinoma*. N Engl J Med, 2009. **361**(10): p.947-57.

12. Soda, M. et al., *Identification of the transforming EML4-ALK fusion gene in non-small cell lung cancer*. Nature, 2007. **448**(7153): p.561-6.

13. Kwak, E.L. et al., *Anaplastic lymphoma kinase inhibition in non-small cell lung cancer*. N

This material is the property of the Wales Cancer Trials Unit. Do not disclose or use except as authorised.

Engl J Med, 2010. **363**(18): p.1693-703.

14. Farmer, H. et al., *Targeting the DNA repair defect in BRCA mutant cells as a therapeutic strategy*. Nature, 2005. **434**(7035): p.917-21.

15. Fong, P.C. et al., *Inhibition of poly(ADP-ribose) polymerase in tumours from BRCA mutation carriers*. N Engl J Med, 2009. **361**(2): p.123-34

16. Paul, I. et al., *PARP Inhibition induces BAX/BAK-independent synthetic lethality of BRCA1-deficient non-small cell lung cancer*. J Pathol, 2011. **224**(4): p.564-74.

17. Quinn, J.E. et al., *BRCA1 functions as a differential modulator of chemotherapy-induced apoptosis*. Cancer Res, 2003. **63**(19): p.6221-8.

18. Font, A. et al., *BRCA1 mRNA expression and outcome to neoadjuvant cisplatin-based chemotherapy in bladder cancer*. Ann Oncol, 2011. **22**(1): p.139-44.

19. Massuti, B. et al., *SCAT Trial: Phase III Spanish customized adjuvant treatment according BRCA1 mRNA levels in stage II-IIIA non-small cell lung cancer*. J Clin Oncol. **29**:2011 (suppl; abstr TPS208).

20. Taron, M. et al., *BRCA1 mRNA expression levels as an indicator of chemoresistance in lung cancer*. Hum Mol Genet, 2004. **13**(20): p.2443-9.

21. Dedes, K.J. et al., *Synthetic lethality of PARP inhibition in cancer lacking BRCA1 and BRCA2 mutations*. Cell Cycle. **10**(8): p.1192-9.

22. Dedes, K.J. et al., *PTEN deficiency in endometrioid endometrial adenocarcinomas predicts sensitivity to PARP inhibitors*. Sci Transl Med. **2**(53): p.53ra75.

23. Mendes-Pereira, A.M. et al., *Synthetic lethal targeting of PTEN mutant cells with PARP inhibitors*. EMBO Mod Med, 2009. **1**(6-7): p.315-22.

24. Sourisseau, T. et al., *Aurora-A expressing tumour cells are deficient for homology-directed DNA double strand-break repair and sensitive to PARP inhibition*. EMBO Mol Med, 2010. **2**(4): p.130-42

25. Ledermann, J. et al., *Phase II randomized placebo-controlled study of Olaparib (AZD2281) in patients with platinum-sensitive relapsed seruos ovarian cancer* (PSR SOC). J Clin Oncol, 2011. **29**:(suppl; abstr 5003).

Appendix A: A guide to performing causality assessments

The following factors should be considered when deciding if there is a "reasonable possibility" that an SAE may have been caused by the drug.

- Time course. Exposure to suspect drug. Has the subject actually received the suspect drug? Did the SAE occur in a reasonable temporal relationship to the administration of the suspect drug?
- Consistency with known drug profile. Was the AE consistent with the previous knowledge of the suspect drug (pharmacology and toxicology) or drugs of the same pharmacological class? Or could the SAE be anticipated from its pharmacological properties?
- Dechallenge experience. Did the SAE resolve or improve on stopping or reducing the dose of the suspect drug?
- No alternative cause. The SAE cannot be reasonably explained by another aetiology such as the underlying disease, other drugs, other host or environmental factors.
- Laboratory tests. A specific laboratory investigation (if performed) has confirmed the relationship?
- A "reasonable possibility" could be considered to exist for an SAE where one or more of these factors exist.

In contrast there would not be a "reasonable possibility" of causality if none of the above criteria apply or where there is evidence of exposure and a reasonable time course but any dechallenge (if performed) is negative or ambiguous or there is another more likely cause of the SAE.

In difficult cases other factors should be considered such as:

- Is this a recognised feature of overdose of the drug?
- Is there a known mechanism?

Ambiguous cases should be considered as being a "reasonable possibility" of causal relationship unless further evidence becomes available to refute this. Causal relationship in cases where the disease under study has deteriorated due to lack of effect should be classified as no reasonable possibility.

CONFIDENTIAL
This material is the property of the Wales Cancer Trials Unit. Do not disclose or use except as authorised.

Short title:	ISAP / SAP Template

Statistical Analysis Plan for

PIN : Parp Inhibitor in advanced NSCLC: A randomised phase II trial of Olaparib maintenance versus placebo monotherapy in patients with chemosensitive advanced non-small cell lung cancer

Eudract/ISRCTN No:	2012-003383-51	Version Number:	2.0 FINAL dated 4th February 2019

**[Final Plan**
Based on protocol version: Version 9.0 dated 11th July 2017

**SAP Revision History**				
Protocol version	Updated Sap version no.	Section number changed	Description and reason for change	Date changed
9.0	2	6.1.2	Sentence added to clarify the plan in the case of missing data	22/1/19
9.0	2	6.1.5	Sentence added to describe Objective Response Rate	22/1/19
9.0	2	6.2.1.1	Sentence added to clarify that PFS will be calculated for the ITT and PP populations and the Cox-model adjusted analysis	22/1/19

9.0	2	6.2.1.2	Sentences added that will present number of patients discontinuing due to adverse events, and that the number of SAEs and deaths due to adverse events will be presented. Median duration of treatment will be presented. ORR at 6 weeks post randomisation will be presented. Further description of the overall survival analysis has been added. The change of size of measurable lesions will be presented using Spider plots rather than Waterfall plots.	22/1/19
9.0	2	Table 12	Changed to remove Clinical Benefit rate and include a column for ORR	22/1/19
9.0	2	Table 13	Changed to show one-sided p-value and overall survival in months	22/1/19

ROLES AND RESPONSIBILITIES

**Trial Statistician: Catharine Porter**			
**Role: Research Assistant (Statistics)**			
Date:		Signature:	
**Senior Statistician: Angela Casbard**			
**Role: Scientific lead**			
Date:	23/10/20	Signature:	Angela Casbard

Short title:	ISAP / SAP Template

**Chief Investigator: Dean Fennell**			
**Role: Chief Investigator**			
Date:		Signature:	
**Other non-signatory contributor to the ISAP/SAP:**			
**Role:**			

TABLE OF CONTENTS

1. INTRODUCTION 6
2. BACKGROUND 6
   2.1 RATIONALE AND RESEARCH QUESTION 6
   2.2 OBJECTIVES 7
3. STUDY MATERIALS 7
   3.1 TRIAL DESIGN 7
   3.2 RANDOMISATION 10
   3.3 BLINDING 10
   3.4 SAMPLE SIZE 10
   3.5 FRAMEWORK 10
   3.6 INTERIM ANALYSES 10
   3.7 TIMING OF FINAL ANALYSIS 11
   3.8 TIMING OF OUTCOME ASSESSMENT 12
4. PRE-ANALYSIS QUALITY CONTROL 14
5. STATISTICAL PRINCIPLES 14

5.1 LEVELS OF CONFIDENCE AND P-VALUES 14
5.2 ADJUSTMENT FOR MULTIPLICITY 14
5.3 ADHERENCE AND PROTOCOL DEVIATIONS 14
5.3.1 DEFINITION AND ASSESSMENT OF ADHERENCE 14
5.6 SCREENING DATA 15
5.7 ELIGIBILITY 15
5.8 RECRUITMENT 15
5.9 WITHDRAWAL/FOLLOW UP 15
5.9.1 LEVEL OF WITHDRAWAL 16
5.9.2 TIMING OF WITHDRAWAL 16
5.9.3 REASONS FOR WITHDRAWAL 16
5.9.4 PRESENTATION OF WITHDRAWAL/LOSS TO FOLLOW-UP 16
5.10 BASELINE PARTICIPANT CHARACTERISTICS 16
5.10.1 LIST OF BASELINE DATA 16
5.10.2 DESCRIPTIVE STATISTICS 16
6. ANALYSIS 17
6.1 OUTCOME DEFINITIONS 17
6.1.1 PRIMARY OUTCOME(S) 17
6.1.2 TIMING, UNITS AND DERIVATION OF PRIMARY 17
6.1.3 LIST OF SECONDARY OUTCOMES 17
6.1.4 ORDER OF TESTING 17
6.1.5 TIMING, UNITS AND DERIVATION OF SECONDARYS 17
6.2 ANALYSIS METHODS 18
6.2.1 LIST OF METHODS AND PRESENTATION 18
6.2.1.1 Primary efficacy analyses 18
6.2.1.2 Secondary analyses 18
6.2.2 COVARIATE ADJUSTMENT 19
6.2.3 ASSUMPTION CHECKING 19
6.2.4 ALTERNATIVE METHODS IF DISTRIBUTIONAL ASSUMPTIONS NOT MET. 19
Not applicable as Kaplan-Meier and log-rank are non-parametric tests. 19

6.2.5 SENSITIVITY ANALYSES 19
6.2.6 SUBGROUP ANALYSES 20
6.3 MISSING DATA 20
6.4 ADDITIONAL ANALYSES 20
6.5 HARMS 20
6.6 STATISTICAL SOFTWARE 20
7. REFERENCES 20
7.1 NON STANDARD STATISTICAL METHODS 20
7.2 DATA MANAGEMENT PLAN 21
7.3 TRIAL MASTER FILE AND STATISTICAL MASTER FILE 21
7.4 OTHER SOPS OR GUIDANCE DOCUMENTS 21
7.5 PUBLISHED LITERATURE 21
8. APPENDICES 24
Appendix 1: Figures 24
.... **Error! Bookmark not defined.**
Appendix 2: Tables 26

1. INTRODUCTION

This statistical analysis plan provides guidelines for the final presentation and analysis for the PIN trial. This plan, along with all other documents relating to the analysis of this trial, will be stored in the Statistical Analysis Master File electronically and/or in hard signed copy formats.

2. BACKGROUND

2.1 RATIONALE AND RESEARCH QUESTION

In 2010 more than 35,000 people died in the UK from lung cancer, the majority from non-small cell cancer (NSCLC). Chemotherapy is one of the main treatments for patients with advanced NSCLC but those treated will still only live for an average of 9 or 10 months after diagnosis. The purpose of this clinical trial is to find out whether or not giving a drug called Olaparib following chemotherapy will benefit patients with NSCLC who have responded to initial chemotherapy treatment by prolonging the time before the tumour re-grows. Sixty-eight patients who have responded to chemotherapy will be randomly allocated to receive either Olaparib or an inactive dummy 'placebo' tablet by mouth. The rationale for this clinical trial is that chemotherapy damages tumour cell DNA and that NSCLC tumours that respond to chemotherapy are less able to repair this damage. This can be exploited by using Olaparib, a drug which blocks an enzyme called Poly (ADP-ribose) polymerase (PARP) which is essential for DNA repair. This will prevent DNA repair and cause cancer cell death by a mechanism known as synthetic lethality. Synthetic lethality arises when a combination of mutations in two or more genes leads to cell death. If this study shows that Olaparib does delay disease progression, a larger more detailed clinical trial will be needed to find out whether using Olaparib actually makes patients live longer.

Rationale for a switch maintenance clinical trial of PARP inhibition in platinum doublet responders

By regulating DNA repair (homologous recombination), BRCA1 expression levels regulate sensitivity to platinum chemotherapy [1-3]. Studies exploring the correlation between BRCA1 mRNA level and response to platinum based therapy have shown a strong inverse predictive interaction [4-5] However, it is believed that low BRCA1 levels are likely to account for only a proportion of highly platinum responsive NSCLCs, with potential defects in other homologous recombination regulating genes underpinning sensitivity. This is because emerging evidence from functional genomic studies indicate that synthetic lethality to PARP inhibition can occur in the absence of somatic alterations in BRCA1 function [6], eg. PTEN deficiency[7, 8], aurora A expression [9].

The total number of cycles of platinum-based chemotherapy that can be given to patients with stage IIIB/IV NSCLC is between 4 and 6, because of toxicity. But if effective therapy could be maintained, disease control, quality of life and overall survival might be prolonged. Exploiting somatic homologous recombination defects, including loss of BRCA1 expression, should confer sensitivity both to PARP inhibition targeted at the cancer and to platinum-based chemotherapy. So response to initial optimal

platinum based chemotherapy could be used to identify those patients likely to have DNA repair deficient NSCLC. They could then receive cancer-specific therapy with PARP inhibitor to exploit the homologous recombination defect and to induce NSCLC cell synthetic lethality.

Olaparib

Olaparib (AZD2281, KU-0059436) is a potent inhibitor of poly (ADP-ribose) polymerase enzyme (PARP), (molecular weight 434) which is being developed as a monotherapy as well as for combination with chemotherapy and other anti-cancer agents. Olaparib can lead to tumour regression in patients with DNA repair deficient NSCLC. Olaparib may also enhance the DNA damaging effects of chemotherapy. Within the clinical development programme for Olaparib, the intention is to assess tolerability and efficacy of Olaparib in patients with advanced DNA repair deficient solid tumours, such as hereditary BRCA-associated cancers and serous ovarian cancers. In such tumour types, Olaparib may offer a potentially efficacious and less toxic cancer treatment compared to currently available chemotherapy regimens.

A recent UK phase IIb trial of maintenance Olaparib (treatment with Olaparib following completion of induction chemotherapy) in patients with ovarian cancer met its primary endpoint of progression-free survival (HR, 0.35; 95% CI 0.25–0.47; P<0.00001; median 8.3 vs 3.7 months) [10]. This trial closely resembles the proposed PIN study, which strengthens the rationale for our trial design.

2.2 OBJECTIVES

Primary:

To investigate the anti-tumour activity of Olaparib as maintenance therapy as measured by PFS (time-to-event).

Secondary:

- To determine the toxicity of Olaparib as maintenance therapy.
- To establish the objective response rate as assessed by RECIST v1.1.
- To determine overall survival (OS), time from randomisation to death with those still alive censored at date last seen.
- To establish change in tumour volume – from randomisation to 6 weeks

3. STUDY MATERIALS

3.1 TRIAL DESIGN

This is a multi-centre double blind randomised phase II trial. Patients have the option to be initially registered to a tissue collection study before induction chemotherapy. These patients will be asked to provide a translational blood sample pre-chemotherapy. All patients will be asked to consent to archival tissue collection for translational analysis, although the timing of this request will depend

upon whether or not they have been registered to stage 1 initially. Another consent (to stage 2 of the trial) will precede randomisation to one of two groups of maintenance therapy (Olaparib or placebo) with 1:1 randomisation if they have evidence of radiological response following standard chemotherapy. This can include mixed stable/response or evidence of tumour shrinkage that does not reach the criteria of partial response according to RECIST.

Patients who are randomised to stage 2 of the trial will receive Olaparib or placebo until disease progression. They will be monitored by CT scan every two cycles until disease progression, when they will be managed according to local practice. Follow up will be for a maximum of 12 months from the point of randomisation or until disease progression. For patients who respond well to Olaparib or placebo, there is the option to continue to receive treatment beyond 60 weeks, provided there are no other safety or efficacy considerations, and provided that the PI has signed written confirmation that this is for the benefit of the patient.

Registered patients with progressive disease prior to randomisation will be asked to provide a follow-up blood sample at the end of induction chemotherapy. We will also ask for a blood sample upon randomisation for those patients who go through to the randomisation stage. However, patients with stable disease that is not eligible for stage 2 will not be asked for any further samples. All randomised patients for whom we have a baseline translational blood sample will be asked to provide a follow-up blood sample upon radiological progression. The trial schema is shown in Figure .

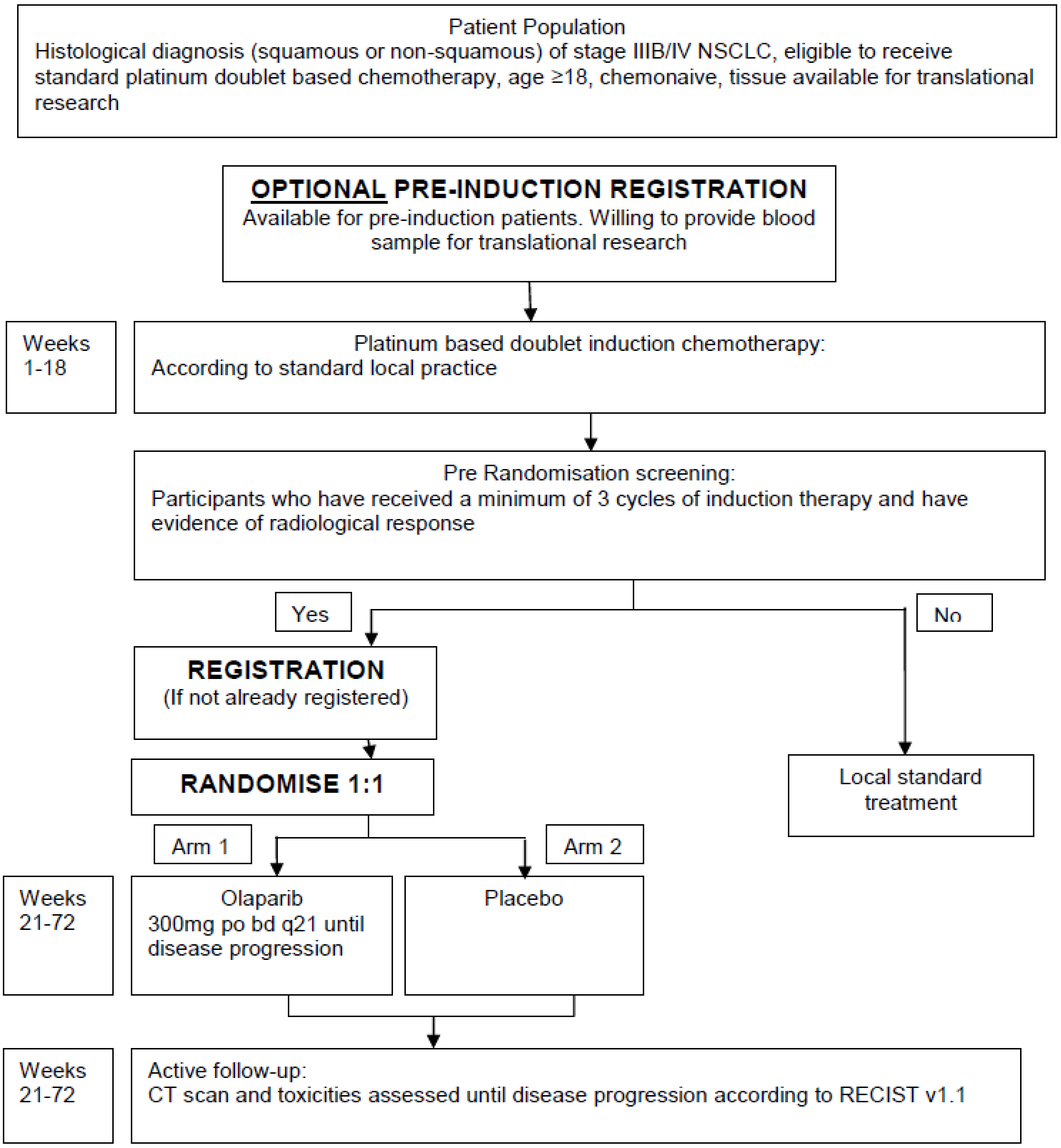

Figure A: Trial Schema

3.2 RANDOMISATION

Randomisation will take place centrally via the interactive web response system (IWRS) after confirmation of eligibility. Participants will be randomised using stratified permuted blocks. This will ensure balanced treatment allocation by a number of clinically important stratification factors (smoking status and histology). Randomisation will have an allocation ratio of 1:1.

3.3 BLINDING

This is a double-blind trial: patients and clinicians are blinded to the olaparib or placebo tablet.

Individual treatment kit codes, indicating the treatment randomisation for each randomised participant, will be available to the investigator(s) or pharmacists from the IWRS. The kit code is different for each bottle of drug.

Staff at the Centre for Trials Research are also blinded to treatment allocation, with the exception of the safety team who can unblind individual patients for SUSAR reporting (blinding is maintained at site). The safety team also provide blinded grouping information to the trial statisticians for the purposes of producing IDMC reports.

3.4 SAMPLE SIZE

The original sample size was based on 90% power and a one-sided α (type I error) of 0.2, 98 events and 114 participants are required in total to demonstrate statistical significance between the arms if the true hazard ratio for Olaparib compared to placebo is 0.65, based on the logrank test. We therefore aimed to recruit 57 participants into each arm, over a 12 month accrual period, with minimum participant follow-up of at least 6 months prior to disease progression, making the total trial length 1.5 years.

Due to much slower than expected recruitment, it was agreed by the TMG, IDMC, TSC and funders that the power of the trial should be lowered to complete recruitment. With 80% power and a one-sided α (type I error) of 0.2, 68 participants are required in total to demonstrate statistical significance between the arms if the true hazard ratio for Olaparib compared to placebo is 0.65, based on the logrank test. We therefore aim to recruit 34 participants into each arm, with a minimum participant follow-up of at least 6 months prior to disease progression. The primary analysis of data will be analysed after 63 PFS events.

3.5 FRAMEWORK

This is a phase 2, parallel-group screening design comparing progression-free survival between the treatment and placebo arms.

3.6 INTERIM ANALYSES

IDMC analyses will be carried out after 10 and 20 patients are randomised on the trial and 6 monthly thereafter.

3.7 TIMING OF FINAL ANALYSIS

The trial data will be analysed when all participants have completed a minimum 6 months follow up and at least 63 disease progression events are observed.

3.8 TIMING OF OUTCOME ASSESSMENT

Assessments	Stage 1 entry (optional)	Post induction chemotherapy	Stage 2 entry (randomisation)	Day -4 to 1 of each 21 day cycle	Daily	Upon disease progression	30 day post treatment visit
Informed consent	X		X				
Medical history	X	X[5]					
Registration	X	X[5]					
CT scan	X	X		X[1]			
RECIST Disease evaluation		X		X[2]			
Co-morbidities check	X						
Pregnancy test			X				
Physical exam			X	X			X
Vital signs			X	X			X
ECOG performance status			X	X			X
Urine Dipstick			X	X			
Serum biochemistry			X	X			
Peripheral blood smear[3]			X				
Full blood count			X	X			
ECG			X	X[4]			X
Inclusion / exclusion criteria	X		X				
Randomisation			X				
Toxicity Assessment			X	X			X
Concomitant medication			X	X			
**Treatments**							
Chemotherapy		X					
Olaparib / placebo				X	X		
Dose delays or reductions				X			

[1]&[2] CT scan and RECIST assessment every 2 cycles only – scan should be performed in the 7 days before the next cycle of treatment
[3] To be done if blood count/morphology suggestive of MDS/AML
[4] To be taken at 9 week visit only
[5] Only taken from Stage 1 participants if they have disease response or disease progression. A blood sample from Stage 1 participants with stable disease is not required.
[6] If not already registered to stage 1 of the trial
[7] Archival tumour biopsy
[8] Optional tumour biopsy sample on progression

	Stage 1 entry (optional)	Post induction chemotherapy	Stage 2 entry (randomisation)	Day -4 to 1 of each 21 day cycle	Daily	Upon disease progression	30 day post treatment visit
**Translational Samples**							
Translational blood	X	X[5]	X[6]			X	
Tumour biopsy sample	X[7]		X (diagnostic)[6]			X[8]	

3.9 PRE-ANALYSIS QUALITY CONTROL

Pre-analysis quality control is detailed in the PIN Data Cleaning Plan.

4. STATISTICAL PRINCIPLES

4.1 LEVELS OF CONFIDENCE AND P-VALUES

80% confidence intervals will be presented for the primary endpoint. 95% confidence intervals will be presented for all other continuous secondary endpoints and any categorical secondary endpoints using Pearson-Clopper or Fisher's exact test.

4.1.1 ADJUSTMENT FOR MULTIPLICITY

No adjustments for multiplicity will be required

4.2 ADHERENCE AND PROTOCOL DEVIATIONS

4.2.1 DEFINITION AND ASSESSMENT OF ADHERENCE

Adherence data will be presented in Table 3 (see Section 6.2.1.2)

4.2.2 PRESENTATION OF ADHERENCE

See Section 6.2.1.2

4.2.3 DEFINITION OF PROTOCOL DEVIATION

Protocol deviations likely to have an effect on the estimation of primary and secondary endpoints will be presented. Examples are missing RECIST assessments, missing translational blood or tissue samples, blood samples taken but not processed within specified time frame, missing toxicity assessments, missing data on dose taken or delays, extra dose taken in error, registration after starting induction chemotherapy leading to missed pre-registration translational samples.

4.2.4 PRESENTATION OF PROTOCOL DEVIATIONS

A summary of the number of protocol deviations will be presented in the CONSORT flow diagram by trial arm (see Appendix 1 Figure 1)

4.3 ANALYSIS POPULATION

At the end of the trial both an intention-to-treat (all results analysed according to the participants' original trial arm allocation) and a per protocol analysis will be carried out. We will attempt to trace participants who are lost to follow-up via their GP or through the NHS-IC. Where no information is available these participants will be censored at the date last seen. Efficacy analyses will be performed for both the intention-to-treat (ITT) population and per-protocol (PP) population. The ITT population will consist of all randomized subjects. The ITT population will be given the greatest weight and the PP

population will be a sensitivity analysis. The PP population will exclude all subjects in the ITT population who meet any of the following criteria:

- A Did not receive at least one IMP or placebo
- B Found to be ineligible
- C RECIST unevaluable at baseline
- D Other protocol deviation likely to have an effect on the estimation of primary and secondary endpoints

Safety analyses will be performed on the safety population, which will be comprised of all randomized subjects who have been administered at least one dose of IMP or placebo.

5. STUDY POPULATION

The Study population will be presented on a CONSORT flow diagram (Appendix 1 Figure 1) as detailed below:

5.1 SCREENING DATA

Screening data will be presented on the CONSORT flow diagram, detailing total numbers of patients assessed for eligibility at all participating centres.

5.2 ELIGIBILITY

The trial inclusion and exclusion criteria are specified in the protocol.

Of those assessed for eligibility, numbers of either excluded or randomised patients will be presented on the CONSORT flow diagram. Numbers of eligible and non-eligible excluded patients will also be presented with reasons for exclusion.

Patients randomized but later found to be ineligible will be listed with reasons for ineligibility.

5.3 RECRUITMENT

Numbers of eligible patients randomised will be presented on the CONSORT flow diagram. Numbers allocated to olaparib or placebo will also be presented.

Start of recruitment: January 2014

End of recruitment: November 2017

Actual recruitment: 70

Target number: 68

5.4 WITHDRAWAL/FOLLOW UP

Numbers of patients completely withdrawing will be presented on the CONSORT flow diagram for each trial arm. Every effort will be made to trace participants who are lost to follow-up via the site. Numbers of patients lost to follow up will be presented on the CONSORT flow diagram

5.4.1 LEVEL OF WITHDRAWAL

There are 4 withdrawal levels for PIN:

1) Withdraw from trial treatment – participants stop trial treatment but remain in follow up and continue to provide translational samples if applicable.
2) Withdraw from the translational study – participants continue trial treatment and follow-up but do not provide translational samples.
3) Withdrawal from the translational study and trial treatment - participants stop trial treatment and do not continue providing translational samples but remain in follow-up.
4) Completely withdraw from the trial – participants stop trial treatment, follow-up and any translational sample collection.

Those completely withdrawing from the trial will be presented on the CONSORT flow diagram. Data for completely withdrawn patients will be used up to the date of withdrawal.

5.4.2 TIMING OF WITHDRAWAL

Numbers of patients completely withdrawing (Level 4) before treatment and during treatment will be presented on the CONSORT flow diagram. Numbers of patients still lost to follow up during treatment and follow up after these checks have been made will be presented on the CONSORT flow diagram.

5.4.3 REASONS FOR WITHDRAWAL

Reasons for withdrawal will be presented on the CONSORT flow diagram including numbers of patients withdrawing for each reason

5.4.4 PRESENTATION OF WITHDRAWAL/LOSS TO FOLLOW-UP

As detailed above.

5.5 BASELINE PARTICIPANT CHARACTERISTICS

Medical history and baseline characteristics will be presented by trial arm (see Table 1 and Table 2). Discrepancies in stratification factors reported at randomization will be checked with sites and any remaining discrepancies will be reported in Table 2.

5.5.1 LIST OF BASELINE DATA

Data for baseline comparability of randomised groups is detailed in Table 1 and Table 2.

5.5.2 DESCRIPTIVE STATISTICS

Data will be presented as numbers (N) and %, by trial arm, or median and IQR, by trial arm as appropriate (see Table 1 and Table 2).

6. ANALYSIS

6.1 OUTCOME DEFINITIONS

6.1.1 PRIMARY OUTCOME(S)

Progression-free survival (PFS - time to event) based on Response Evaluation Criteria in Solid Tumours (RECIST) v1.1.

6.1.2 TIMING, UNITS AND DERIVATION OF PRIMARY

- Progression-free survival will be measured in weeks post randomization. Event is defined as disease progression or death from any cause. Time to event will be calculated as days from the date of randomisation to the date of event (date of RECIST assessment or date of death). Those still alive and progression free will be censored at the date last seen. However, if the patient progresses or dies after two or more missed visits, the patient will be censored at the time of the latest evaluable RECIST 1.1 assessment prior to the two missed visits.
- If the patient has no evaluable visits or does not have baseline data they will be censored at Day 1 unless they die within 2 visits of baseline (16 weeks plus 1 week allowing for a late assessment within the visit window).

Disease progression is formally assessed according to the RECIST v1.1 criteria (Eisenhower et al 2009).

6.1.3 LIST OF SECONDARY OUTCOMES

- Tolerability (side effects) and feasibility of use (number of participants requiring dose delays or reductions and/or treatment withdrawal).
- Objective response rate (ORR) as assessed by RECIST criteria
- Overall survival (OS). Time from randomisation to death from any cause. Those still alive will be censored at time last seen.
- To measure anti-tumour activity in terms of tumour volume.
- To collect tumour and blood samples for translational work including investigating BRCA1 expression. Translational sample analysis will be performed for a separate sub-study and not for this main paper analysis.

6.1.4 ORDER OF TESTING

Not applicable

6.1.5 TIMING, UNITS AND DERIVATION OF SECONDARYS

Overall survival will be measured in days post randomization and summarized in months.

- ORR will be defined as the best response seen at any time in the trial for CR and PR (excluding any responses seen after subsequent anti-cancer therapy).

6.2 ANALYSIS METHODS

6.2.1 LIST OF METHODS AND PRESENTATION

6.2.1.1 Primary efficacy analyses

Primary analysis will be of Progression-Free Survival, time from randomisation to disease progression or death from any causes (whichever occurs first) with those still alive and progression free censored at date last seen, described using Kaplan-Meier curves in both arms of the trial. p-values, HR and 95% confidence intervals will be presented for the ITT, PP and Cox model adjusted populations (Figure 2). The number of deaths, progressions and PFS events and the median PFS (with 95% CI) will be calculated for each arm of the trial for the ITT and PP populations (Table 13), and then the one-sided logrank test will be used to formally test the equality of the survivor functions. If the hazards are proportional, Cox regression can also be performed to adjust the hazard ratio for the stratification factors (smoking status and histology).

6.2.1.2 Secondary analyses

Secondary analysis will be as follows:

- Tolerability-Toxicity will be evaluated by tabulations of adverse events and will be presented with descriptive statistics at baseline and over visits for both study arms. Baseline toxicities with any grade (N, %) (Table 8), worst reported toxicities with any grade and grade 3+, overall for Cycle 1 to 30 days post treatment (N, %) (Table 9**Error! Reference source not found.**) and emerging toxicities not reported at baseline with any grade and grade 3+, overall for Cycle 1 to 30 days post treatment (N, %) (Table 10) will be reported. Adverse event incidence rates will be summarized by system organ class, preferred term, and severity of the adverse event. Each subject will be counted only once within a system organ class and preferred term by using the adverse events with the highest severity within each category. The numbers (n, %) of patients discontinuing trial treatment due to adverse events will be presented in the text. The number of SAEs (n, %) and deaths due to adverse events (n, %) will be presented in the text.
- Feasibility of use will be presented in Table 3. Descriptive statistics on olaparib/placebo treatment administration (N=number of patients receiving treatment, and dose administered in mg (median (IQR)), number of patients with olaparib/placebo dose reduction (N, %) and number of patients with olaparib/placebo dose delay (N, %) will be presented by unblinded trial arm for each cycle. Median (IQR) dose intensity will be presented for each arm (using the following formula).
    - PD=Planned protocol dose per 21 day Cycle=300x2x21=12600mg
    - AD= Actual dose per 21 day cycle = (Dose prescribedx2x21) -N tablets missed x150(mg)-N tablets missed x100(mg) – (N days delayed/withdrawn x prescribed dose x 2)
    - Relative dose intensity RDI = sum of AD for each cycle received /(PDx[days until end of trial / 21])

The median duration of treatment (with IQR) will be presented in the text for each trial arm.

- The objective response rate will be summarised, based on Radiological disease progression defined according to RECIST (Response Evaluation Criteria In Solid Tumours) v1.1. The objective response rate at 6 weeks post randomisation will be presented in the text, where response is defined as complete or partial response. The proportions of participants in each objective disease response (RECIST v1.1) category will be reported at each CT scan (Table 12). p-values from either Clopper-Pearson or Fisher's exact test will be presented in the text.
- Overall survival (OS) for the ITT population will be calculated from the time of randomisation to date of death or date censored (date last known to be alive). The number of deaths will be reported and the OS will be presented as median survival (with IQR) (Table 13) and with Kaplan-Meier curves (Figure 3Figure 1Figure 3) in both trial arms. The one-sided logrank test will be used to formally test the equality of the survivor functions, and HR, 95% CI and p-value will be presented in Table 13. Median duration of follow-up (with IQR) for those progression-free and alive will be presented in the text and Table 13.
- The change of size of measurable lesions at each disease assessment after start of chemotherapy relative to baseline will be assessed using a Spider plot, by trial arm (Figure 4). This will only be presented if there is a significant difference between trial arms.
- Translational blood sample collection at disease progression (N, %) or end of cycle 18 blood sample if the participant has not progressed (N, %), will be presented by trial arm in Table 6.

6.2.2 COVARIATE ADJUSTMENT

For the primary analysis of Progression-Free Survival, if the hazards are proportional, then Cox regression will also be performed to adjust the hazard ratio for the stratification factors. Stratification factors are smoking status (never smoked or ever smoked) and histology (squamous or non-squamous). As a sensitivity analysis, the hazard ratio will also be adjusted for any unbalanced baseline characteristics. If there are discrepancies between the stratification groups given at randomization and the true stratification groups, a sensitivity analysis will be performed to include an adjustment for the true results.

6.2.3 ASSUMPTION CHECKING

Prior to Cox regression the proportionality of hazards assumption will be tested. Under the proportionality of hazards assumption, the null hypothesis is that the plot of scaled Schoenfeld residuals of the Cox regression against time has a zero slope. This null hypothesis will be tested overall and for each covariate and plots produced. If the plot of Schoenfeld residuals against time shows a non-random pattern, the proportionality of hazards assumption has been violated.

6.2.4 ALTERNATIVE METHODS IF DISTRIBUTIONAL ASSUMPTIONS NOT MET

Not applicable as Kaplan-Meier and log rank are non-parametric.

6.2.5 SENSITIVITY ANALYSES

See section 6.2.2

6.2.6 SUBGROUP ANALYSES

No subgroup analyses are planned.

6.3 MISSING DATA

Every effort will be made to obtain data on patients lost to follow up via the site. Participants still missing date of disease progression or death will be censored on date last seen. Every effort will be made to collect the data by querying study sites. The primary outcome analysis, progression-free survival, should be subject to no missing data; no imputation will be performed.

6.4 ADDITIONAL ANALYSES

The following analyses during treatment and 30 days post treatment will also be presented:

- Table 4-Physical examination including ECOG performance status and weight (cycles 1 to 18 and 30 days post treatment)
- Table 5-Urinalysis and blood tests (cycles 1 to 18 and 30 days post treatment)
- Table 7-Cardiac assessment, ECG repeated at end of cycle 3 and 30 days post treatment
- Table 11-Radiological disease assessment (cycle 2 then every second cycle up to cycle 18)
- Table 14**Error! Reference source not found.**-Summary statistics of level of withdrawal and cause of withdrawals and deaths

6.5 HARMS

Adverse event and serious adverse events are reported in the secondary analyses. For reporting of adverse event data to EudraCT, tabulations of non-serious adverse events (experienced in >5% of participants) will be presented (Table 15: EudraCT reportable non-serious adverse events). Non-serious adverse events will be classified on the basis of CTCAE terminology. All serious adverse events will also be presented (Table 16). Serious adverse events will be classified on the basis of MedDRA terminology. Serious adverse events will be summarized by system organ class, preferred term, and relationship to procedure. Each subject will be counted only once within a system organ class or a preferred term by using the serious adverse events with the closest relationship to treatment within each category.

6.6 STATISTICAL SOFTWARE

All statistical analysis will be performed using STATA 14

7. REFERENCES

7.1 NON STANDARD STATISTICAL METHODS

Not applicable

7.2 DATA MANAGEMENT PLAN

The current Data Management Plan can be found at \\wctunas1\Trials\WCTU Trials\PIN\16. Data Management\Data Management Plan\CURRENT\ PIN DMP V3.0 30.05.2018.docx

7.3 TRIAL MASTER FILE AND STATISTICAL MASTER FILE

The Trial Master File is held by the PIN Trial Manager. A separate Statistical Master File is held securely and is only accessible by the Trial Statistician and Senior Statistician.

7.4 OTHER SOPS OR GUIDANCE DOCUMENTS

SOP/007/7 and template TPL/007/6 were used to create the PIN Data Cleaning Plan. The Data Cleaning Plan can be found at \\wctunas1\Trials\WCTU Trials\PIN\16. Data Management\Data Cleaning for Final Statistical Analysis\Data Cleaning Plan\Final\ PIN Data Cleaning Plan Version 1 FINAL dated 11062018.docx

7.5 PUBLISHED LITERATURE

1. Paul, I. et al., *PARP Inhibition induces BAX/BAK-independent synthetic lethality of BRCA1-deficient non-small cell lung cancer*. J Pathol, 2011. **224**(4): p.564-74.

2. Quinn, J.E. et al., *BRCA1 functions as a differential modulator of chemotherapy-induced apoptosis*. Cancer Res, 2003. **63**(19): p.6221-8.

3. Font, A. et al., *BRCA1 mRNA expression and outcome to neoadjuvant cisplatin-based chemotherapy in bladder cancer*. Ann Oncol, 2011. **22**(1): p.139-44.

4. Massuti, B. et al., *SCAT Trial: Phase III Spanish customized adjuvant treatment according BRCA1 mRNA levels in stage II-IIIA non-small cell lung cancer*. J Clin Oncol. **29**:2011 (suppl; abstr TPS208).

5. Taron, M. et al., *BRCA1 mRNA expression levels as an indicator of chemoresistance in lung cancer*. Hum Mol Genet, 2004. **13**(20): p.2443-9.

6. Dedes, K.J. et al., *Synthetic lethality of PARP inhibition in cancer lacking BRCA1 and BRCA2 mutations*. Cell Cycle. **10**(8): p.1192-9.

7. Dedes, K.J. et al., *PTEN deficiency in endometrioid endometrial adenocarcinomas predicts sensitivity to PARP inhibitors*. Sci Transl Med. **2**(53): p.53ra75.

8. Mendes-Pereira, A.M. et al., *Synthetic lethal targeting of PTEN mutant cells with PARP inhibitors*. EMBO Mod Med, 2009. **1**(6-7): p.315-22.

9. Sourisseau, T. et al., *Aurora-A expressing tumour cells are deficient for homology-directed DNA double strand-break repair and sensitive to PARP inhibition*. EMBO Mol Med, 2010. **2**(4): p.130-42

10. Ledermann, J. et al., *Phase II randomized placebo-controlled study of Olaparib (AZD2281) in patients with platinum-sensitive relapsed seruos ovarian cancer* (PSR SOC). J Clin Oncol, 2011. **29**:(suppl; abstr 5003).

11. Eisenhower, E.A. et al., *New response criteria in solid tumours: revised RECIST guideline (version 1.1)* Eur J Cancer, 2009 **45**(2): p.225-47

SAP/ISAP DEVIATION LOG

**Document number:**		**Document version:**	
**Reason for deviation:**			
Sub-group analysis of squamous and non-squamous overall survival was added prior to finalizing the study report (14/02/2020).			
The following changes were made prior to submission for publication:			
The scale on the KM graphs for overall survival was changed to weeks rather than months to be consistent with progression-free survival (01/04/2020 and 01/10/2020).			
Table 11 was amended to show median change in tumour volume which was reported in the text of the publication (22/04/2020).			

Table 1 was amended to include a summary of recruitment time point. Analysis of chemotherapy regimes was amended to show the complete regimes rather than individual therapies. (01/10/2020).
An analysis of number of dose reductions and discontinuation of treatment after dose reductions was done (22/10/2020).

8. APPENDICES

Appendix 1: Figures

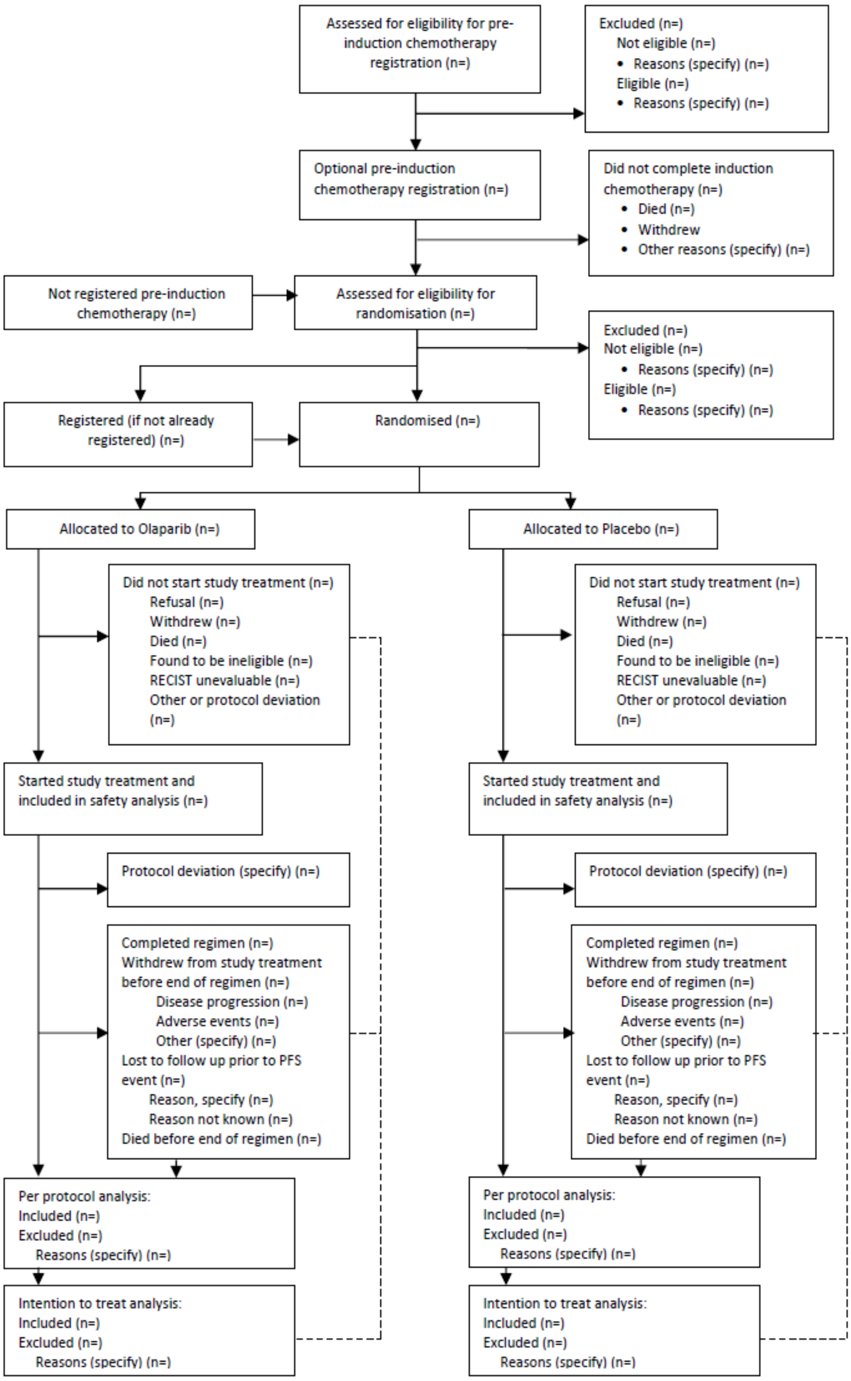

Figure 1: PIN CONSORT diagram

Figure 2: KM of progression-free survival

Show Kaplan-Meier survival curves of study arms measuring the time from enrolment to objective disease progression or death from any causes (whichever occurs first). Include numbers at risk, HR, 95% Confidence intervals and one-sided p-value, for the ITT, PP and Cox model adjusted populations.

Figure 3: KM of overall survival

Show Kaplan-Meier survival curves of study arms measuring the time from enrolment to death from any causes for the ITT population. Include numbers at risk, HR, 95% Confidence intervals and one-sided p-value.

Figure 4: Spider plots showing changes in tumour size at each disease assessment relative to baseline

Show Spider plots showing change in measurable disease at each disease assessment after start of chemotherapy by trial arm, if there is a significant difference between arms.

Appendix 2: Tables

Table 1: Medical History

Table 1: Medical History	Olaparib	Placebo
	N=	N=
**Non-small cell lung cancer type - N (%)**		
Adenocarcinoma		
Squamous		
Mixed		
Unspecified		
Other (specify)		
**T stage - N (%)**		
T1		
T2		
T3		
T4		
Tis		
Tx		
**N stage - N (%)**		
N0		
N1		
N2		
N3		
NX		
**M stage - N(%)**		
M0		
M1a		
M1b		
**Stage of NSCLC - N(%)**		
IIIB		
IV		

Table 1: Medical History	Olaparib	Placebo
	N=	N=
**Sites affected - N (%)**		
Oesophagus		
Chest wall		
Trachea		
Pleura		
Heart pericardium		
Diaphragm		
Liver metastases		
Lung metastases		
Bones metastases		
Brain metastases		
Adrenal gland		
CNS metastases		
Other (specify)		
**Type of induction chemotherapy treatment - N (%)**		
Gemcitabine		
Cisplatin		
Carboplatin		
Pemetrexed		
Paclitaxel		
Vinorelbine		
Other (specify)		
**Number of cycles of induction chemotherapy - n, median (IQR)**		
**Response to induction chemotherapy - N(%)**		
Complete response		
Partial response		
Other evidence of tumour shrinkage/mixed stable		
Stable disease		
Progression		

Table 2: Pre-randomisation baseline characteristics

Table 2: Baseline Characteristics	Olaparib<br>N=	Placebo<br>N=
**Sex - N (%)**		
Male		
Female		
**Age - n, median (IQR)**		
**Smoking history - N (%)**		
Never smoked		
Ever smoked		
**Systolic BP - n, median (IQR)**		
**Diastolic BP - n, median (IQR)**		
**Oxygen Saturation - n, median (IQR)**		
**Pulse - n, median (IQR)**		
**Weight - n, median (IQR)**		
**ECOG status - n, median (IQR)**		
**ECOG status - N (%)**		
0		
1		
2		
3		
4		
**ECG Resting QTc - n, median (IQR)**		
**URINALYSIS**		
**pH Normal - N(%)**		
Yes		
No		
**pH clinically significant - N(%)**		
Yes		
No		
**Glucose Normal - N(%)**		
Yes		
No		
**Glucose clinically significant - N(%)**		
Yes		
No		
**Blood Normal - N(%)**		
Yes		
No		
**Blood clinically significant - N(%)**		
Yes		
No		
**Protein Normal - N(%)**		
Yes		
No		
**Protein clinically significant - N(%)**		
Yes		
No		
**Leucocytes Normal - N(%)**		
Yes		
No		
**Leucocytes clinically significant - N(%)**		
Yes		
No		

Table 2: Baseline Characteristics	Olaparib<br>N=	Placebo<br>N=
**Blood results - n, median (IQR)**		
Serum creatinine - µmol/L		
Urea - mmol/L		
Corrected calcium - mmol/L		
Albumin - g/L		
Neutrophils - 10^9/L		
WBC - 10^9/L		
ALT - IU/L		
ALP - IU/L		
Serum bilirubin - µmol/L		
Potassium - mmol/L		
Sodium - mmol/L		
Platelets - 10^9/L		
Haemoglobin - g/L		
RBC - 10^{12}/L		
AST - IU/L		
**Site of target tumour[(1)]**		
Primary tumour - N (%)		
Lymph node - N (%)		
Brain - N (%)		
Bone - N (%)		
Liver - N (%)		
Adrenal glands - N (%)		
Other - N (%)		
**Longest Diameter of tumours - n, median (IQR)**		
Primary tumour		
Lymph node		
Brain		
Bone		
Liver		
Lung		
Other		
**Sum of Longest Diameters - n, median (IQR)**		
**Pre-induction chemotherapy blood sample was taken for translational purposes - N (%)**		
Yes		
No		
**Post-induction chemotherapy blood sample was taken for translational purposes - N (%)**		
Yes		
No		
**The patient's archival tissue block has been requested for translational purposes - N (%)**		
Yes		
No		

(1) All the patients are included in the analysis who were not withdrawn or died

Table 3: Treatment compliance

Table 3: Treatment Details	Cycle 1		Cycle 2		Cycle 3		Cycle 4		Cycle 5		Cycle 6	
	Olaparib	Placebo	Olaparib	Placebo	Olaparib	Placebo	Olaparib	Placebo	Olaparib	Placebo	Olaparib	Placebo
	N=	N=	N=	N=	N=	N=	N=	N=	N=	N=	N=	N=
Olaparib/placebo given to the patient - N (%)												
Dose of Olaparib/placebo administered - n, median (IQR)												
Dose reduction of Olaparib/placebo - N (%)												
Dose delay of Olaparib/placebo - N (%)												
Withdrawal from trial treatment - N(%)												

	Cycle 7		Cycle 8		Cycle 9		Cycle 10		Cycle 11		Cycle 12	
	Olaparib	Placebo	Olaparib	Placebo	Olaparib	Placebo	Olaparib	Placebo	Olaparib	Placebo	Olaparib	Placebo
	N=	N=	N=	N=	N=	N=	N=	N=	N=	N=	N=	N=
Olaparib/placebo given to the patient - N (%)												
Dose of Olaparib/placebo administered - n, median (IQR)												
Dose reduction of Olaparib/placebo - N (%)												
Dose delay of Olaparib/placebo - N (%)												
Withdrawal from trial treatment - N(%)												

	Cycle 13		Cycle 14		Cycle 15		Cycle 16		Cycle 17		Cycle 18	
	Olaparib	Placebo	Olaparib	Placebo	Olaparib	Placebo	Olaparib	Placebo	Olaparib	Placebo	Olaparib	Placebo
	N=	N=	N=	N=	N=	N=	N=	N=	N=	N=	N=	N=
Olaparib/placebo given to the patient - N (%)												
Dose of Olaparib/placebo administered - n, median (IQR)												
Dose reduction of Olaparib/placebo - N (%)												
Dose delay of Olaparib/placebo - N (%)												
Withdrawal from trial treatment - N(%)												

Table 4: Physical examination and ECOG status

Table 4: Physical Examination, ECOG Status	Cycle 1		Cycle 2		Cycle 3		Cycle 4		Cycle 5		Cycle 6	
	Olaparib	Placebo	Olaparib	Placebo	Olaparib	Placebo	Olaparib	Placebo	Olaparib	Placebo	Olaparib	Placebo
	N=	N=	N=	N=	N=	N=	N=	N=	N=	N=	N=	N=
Systolic BP - n, median (IQR)												
Diastolic BP - n, median (IQR)												
Oxygen Saturation - n, median (IQR)												
Pulse - n, median (IQR)												
Weight - n, median (IQR)												
ECOG status - n, median (IQR)												
ECOG status - N (%)												
0												
1												
2												
3												
4												

	Cycle 7		Cycle 8		Cycle 9		Cycle 10		Cycle 11		Cycle 12	
	Olaparib	Placebo	Olaparib	Placebo	Olaparib	Placebo	Olaparib	Placebo	Olaparib	Placebo	Olaparib	Placebo
	N=	N=	N=	N=	N=	N=	N=	N=	N=	N=	N=	N=
Systolic BP - n, median (IQR)												
Diastolic BP - n, median (IQR)												
Oxygen Saturation - n, median (IQR)												
Pulse - n, median (IQR)												
Weight - n, median (IQR)												
ECOG status - n, median (IQR)												
ECOG status - N (%)												
0												
1												
2												
3												
4												

	Cycle 13		Cycle 14		Cycle 15		Cycle 16		Cycle 17		Cycle 18		30 days post treatment	
	Olaparib	Placebo	Olaparib	Placebo	Olaparib	Placebo	Olaparib	Placebo	Olaparib	Placebo	Olaparib	Placebo	Olaparib	Placebo
	N=	N=	N=	N=	N=	N=	N=	N=	N=	N=	N=	N=	N=	N=
Systolic BP - n, median (IQR)														
Diastolic BP - n, median (IQR)														
Oxygen Saturation - n, median (IQR)														
Pulse - n, median (IQR)														
Weight - n, median (IQR)														
ECOG status - n, median (IQR)														
ECOG status - N (%)														
0														
1														
2														
3														
4														

Short title:	ISAP / SAP Template

Table 5: Urinalysis, blood tests and serum biochemistry

Table 5: Urinalysis, Blood Tests, Serum Biochemistry	Cycle 1		Cycle 2		Cycle 3		Cycle 4		Cycle 5		Cycle 6		Cycle 7		Cycle 8		Cycle 9	
	Olaparib	Placebo	Olaparib	Placebo	Olaparib	Placebo	Olaparib	Placebo	Olaparib	Placebo	Olaparib	Placebo	Olaparib	Placebo	Olaparib	Placebo	Olaparib	Placebo
	N=	N=	N=	N=	N=	N=	N=	N=	N=	N=	N=	N=	N=	N=	N=	N=	N=	N=
**URINALYSIS**																		
**pH Normal - N(%)**																		
Yes																		
No																		
**pH clinically significant - N(%)**																		
Yes																		
No																		
**Glucose Normal - N(%)**																		
Yes																		
No																		
**Glucose clinically significant - N(%)**																		
Yes																		
No																		
**Blood Normal - N(%)**																		
Yes																		
No																		
**Blood clinically significant - N(%)**																		
Yes																		
No																		
**Protein Normal - N(%)**																		
Yes																		
No																		
**Protein clinically significant - N(%)**																		
Yes																		
No																		
**Leucocytes Normal - N(%)**																		
Yes																		
No																		
**Leucocytes clinically significant - N(%)**																		
Yes																		
No																		
**Blood results - n, median (IQR)**																		
Magnesium - mmol/L																		
Sodium - mmol/L																		
Potassium - mmol/L																		
Urea - mmol/L																		
Serum creatinine - µmol/L																		
eGFR - ml/min																		
Albumin - g/L																		
Corrected calcium - mmol/L																		
Serum bilirubin - µmol/L																		
AST - IU/L																		
ALT - IU/L																		
ALP - IU/L																		
Haemoglobin - g/L																		
WBC - 10^9/L																		
Neutrophils - 10^9/L																		
Platelets - 10^9/L																		
Prothrombin time - sec																		
FSH - IU/L																		
Estradiol - pmol/L																		

	Cycle 10		Cycle 11		Cycle 12		Cycle 13		Cycle 14		Cycle 15		Cycle 16		Cycle 17		Cycle 18		30 days post treatment	
	Olaparib	Placebo	Olaparib	Placebo	Olaparib	Placebo	Olaparib	Placebo	Olaparib	Placebo	Olaparib	Placebo	Olaparib	Placebo	Olaparib	Placebo	Olaparib	Placebo	Olaparib	Placebo
	N=	N=	N=	N=	N=	N=	N=	N=	N=	N=	N=	N=	N=	N=	N=	N=	N=	N=	N=	N=
**URINALYSIS**																				
**pH Normal - N(%)**																				
Yes																				
No																				
**pH clinically significant - N(%)**																				
Yes																				
No																				
**Glucose Normal - N(%)**																				
Yes																				
No																				
**Glucose clinically significant - N(%)**																				
Yes																				
No																				
**Blood Normal - N(%)**																				
Yes																				
No																				
**Blood clinically significant - N(%)**																				
Yes																				
No																				
**Protein Normal - N(%)**																				
Yes																				
No																				
**Protein clinically significant - N(%)**																				
Yes																				
No																				
**Leucocytes Normal - N(%)**																				
Yes																				
No																				
**Leucocytes clinically significant - N(%)**																				
Yes																				
No																				
**Blood results - n, median (IQR)**																				
Magnesium - mmol/L																				
Sodium - mmol/L																				
Potassium - mmol/L																				
Urea - mmol/L																				
Serum creatinine - µmol/L																				
eGFR - ml/min																				
Albumin - g/L																				
Corrected calcium - mmol/L																				
Serum bilirubin - µmol/L																				
AST - IU/L																				
ALT - IU/L																				
ALP - IU/L																				
Haemoglobin - g/L																				
WBC - 10^9/L																				
Neutrophils - 10^9/L																				
Platelets - 10^9/L																				
Prothrombin time - sec																				
FSH - IU/L																				
Estradiol - pmol/L																				

Table 6: Collection of translational blood sample at disease progression, or end of cycle 18 if not progressed

Table 6: Collection of translational blood samples at disease progression or end of cycle 18	At disease progression		At end of cycle 18 (if not progressed)	
	Olaparib	Placebo	Olaparib	Placebo
	N=	N=	N=	N=
Blood sample was taken for translational purposes - N (%)				
Yes				
No				

Table 7: Cardiac assessment

Table 7: Cardiac Assessment	Cycle 3		30 days post treatment	
	Olaparib	Placebo	Olaparib	Placebo
	N=	N=	N=	N=
ECG Resting QTc - n, median (IQR)				

Table 8: Baseline toxicities

Table 8: Baseline toxicities	Olaparib Grade reported (N)										Placebo Grade reported (N)									
	1	%	2	%	3	%	4	%	3+	%	1	%	2	%	3	%	4	%	3+	%
**Blood and lymphatic system**																				
Anaemia - N (%)																				
Lymphopenia - N (%)																				
Neutropenia - N (%)																				
Thrombocytopenia - N (%)																				
**Cardiac disorders**																				
Tachycardia - N (%)																				
**Gastrointestinal disorders**																				
Abdominal distension - N (%)																				
Abdominal pain - N (%)																				
Constipation - N (%)																				
Diarrhoea - N (%)																				
Dry mouth - N (%)																				
Dyspepsia - N (%)																				
Flatulence - N (%)																				
Mucosal inflammation - N (%)																				
Nausea - N (%)																				
Vomiting - N (%)																				
**General disorders and administration site conditions**																				
Fatigue - N (%)																				
Fever - N (%)																				
Oedema peripheral - N (%)																				
Palmar-plantar syndrome - N (%)																				
**Infections and infestations**																				
Lower respiratory infection - N (%)																				
Upper respiratory infection - N (%)																				
Urinary tract infection - N (%)																				
Psychiatric disorders - N (%)																				
**Metabolism and nutrition disorders**																				
Anorexia - N (%)																				
Hyponatraemia - N (%)																				
**Musculoskeletal and connective tissue disorders**																				
Arthralgia - N (%)																				
Back pain - N (%)																				
Nervous system disorders - N (%)																				
Dizziness - N (%)																				
Dysgeusia - N (%)																				
Headache - N (%)																				
Nasopharyngitis - N (%)																				
**Psychiatric disorders**																				
Insomnia - N (%)																				
**Respiratory, thoracic and mediastinal disorders**																				
Coughing - N (%)																				
Dyspnoea - N (%)																				
Oropharyngeal pain - N (%)																				
Pneumonitis - N (%)																				
Stomatitis - N (%)																				
**Skin and subcutaneous tissue disorders**																				
Alopecia - N (%)																				
Blisters - N (%)																				
Erythema - N (%)																				
Rash - N (%)																				
**Neoplasms benign, malignant and unspecified**																				
AML - N (%)																				
Pruritis - N (%)																				
Myelodysplastic syndrome - N (%)																				
Vascular disorders - N (%)																				
Hot flushes - N (%)																				
Hypertension - N (%)																				
**Other1 - N (%)**																				
**Other2 - N (%)**																				
**Other3 - N (%)**																				
**etc**																				

Table 9: **Worst Reported Toxicities overall (Cycle 1 to 30 days post treatment), including SAEs**

**Table 9: Worst Reported Toxicities overall (Cycle 1 to 30 days post treatment)**	Olaparib Grade reported (N)										Placebo Grade reported (N)									
	1	%	2	%	3	%	4	%	3+	%	1	%	2	%	3	%	4	%	3+	%
**Any cause**																				
**Blood and lymphatic system**																				
Anaemia - N (%)																				
Lymphopenia - N (%)																				
Neutropenia - N (%)																				
Thrombocytopenia - N (%)																				
**Cardiac disorders**																				
Tachycardia - N (%)																				
**Gastrointestinal disorders**																				
Abdominal distension - N (%)																				
Abdominal pain - N (%)																				
Constipation - N (%)																				
Diarrhoea - N (%)																				
Dry mouth - N (%)																				
Dyspepsia - N (%)																				
Flatulence - N (%)																				
Mucosal inflammation - N (%)																				
Nausea - N (%)																				
Vomiting - N (%)																				
**General disorders and administration site conditions**																				
Fatigue - N (%)																				
Fever - N (%)																				
Oedema peripheral - N (%)																				
Palmar-plantar syndrome - N (%)																				
**Infections and infestations**																				
Lower respiratory infection - N (%)																				
Upper respiratory infection - N (%)																				
Urinary tract infection - N (%)																				
Psychiatric disorders - N (%)																				
**Metabolism and nutrition disorders**																				
Anorexia - N (%)																				
Hyponatraemia - N (%)																				
**Musculoskeletal and connective tissue disorders**																				
Arthralgia - N (%)																				
Back pain - N (%)																				
Nervous system disorders - N (%)																				
Dizziness - N (%)																				
Dysgeusia - N (%)																				
Headache - N (%)																				
Nasopharyngitis - N (%)																				
**Psychiatric disorders**																				
Insomnia - N (%)																				
**Respiratory, thoracic and mediastinal disorders**																				
Coughing - N (%)																				
Dyspnoea - N (%)																				
Oropharyngeal pain - N (%)																				
Pneumonitis - N (%)																				
Stomatitis - N (%)																				
**Skin and subcutaneous tissue disorders**																				
Alopecia - N (%)																				
Blisters - N (%)																				
Erythema - N (%)																				
Rash - N (%)																				
**Neoplasms benign, malignant and unspecified**																				
AML - N (%)																				
Pruritis - N (%)																				
Myelodysplastic syndrome - N (%)																				
Vascular disorders - N (%)																				
Hot flushes - N (%)																				
Hypertension - N (%)																				
**Other1 - N (%)**																				
**Other2 - N (%)**																				
**Other3 - N (%)**																				
**etc**																				

Table 10: Emerging toxicities (not present at or worse since baseline) overall (Cycle 1 to 30 days post treatment), including SAEs

Table 10: Emerging toxicities (not present at baseline) overall (Cycle 1 to 30 days post treatment)	Olaparib										Placebo									
	Grade reported (N)										Grade reported (N)									
	1	%	2	%	3	%	4	%	3+	%	1	%	2	%	3	%	4	%	3+	%
**Any cause**																				
**Blood and lymphatic system**																				
Anaemia - N (%)																				
Lymphopenia - N (%)																				
Neutropenia - N (%)																				
Thrombocytopenia - N (%)																				
**Cardiac disorders**																				
Tachycardia - N (%)																				
**Gastrointestinal disorders**																				
Abdominal distension - N (%)																				
Abdominal pain - N (%)																				
Constipation - N (%)																				
Diarrhoea - N (%)																				
Dry mouth - N (%)																				
Dyspepsia - N (%)																				
Flatulence - N (%)																				
Mucosal inflammation - N (%)																				
Nausea - N (%)																				
Vomiting - N (%)																				
**General disorders and administration site conditions**																				
Fatigue - N (%)																				
Fever - N (%)																				
Oedema peripheral - N (%)																				
Palmar-plantar syndrome - N (%)																				
**Infections and infestations**																				
Lower respiratory infection - N (%)																				
Upper respiratory infection - N (%)																				
Urinary tract infection - N (%)																				
Psychiatric disorders - N (%)																				
**Metabolism and nutrition disorders**																				
Anorexia - N (%)																				
Hyponatraemia - N (%)																				
**Musculoskeletal and connective tissue disorders**																				
Arthralgia - N (%)																				
Back pain - N (%)																				
Nervous system disorders - N (%)																				
Dizziness - N (%)																				
Dysgeusia - N (%)																				
Headache - N (%)																				
Nasopharyngitis - N (%)																				
**Psychiatric disorders**																				
Insomnia - N (%)																				
**Respiratory, thoracic and mediastinal disorders**																				
Coughing - N (%)																				
Dyspnoea - N (%)																				
Oropharyngeal pain - N (%)																				
Pneumonitis - N (%)																				
Stomatitis - N (%)																				
**Skin and subcutaneous tissue disorders**																				
Alopecia - N (%)																				
Blisters - N (%)																				
Erythema - N (%)																				
Rash - N (%)																				
**Neoplasms benign, malignant and unspecified**																				
AML - N (%)																				
Pruritis - N (%)																				
Myelodysplastic syndrome - N (%)																				
Vascular disorders - N (%)																				
Hot flushes - N (%)																				
Hypertension - N (%)																				
**Other1 - N (%)**																				
**Other2 - N (%)**																				
**Other3 - N (%)**																				
**etc**																				

Table 11: Radiological disease assessment

Table 11: Radiological Disease Assessment	Baseline		Cycle 2		Cycle 4		Cycle 6		Cycle 8		Cycle 10		Cycle 12		Cycle 14		Cycle 16		Cycle 18	
	Olaparib	Placebo	Olaparib	Placebo	Olaparib	Placebo	Olaparib	Placebo	Olaparib	Placebo	Olaparib	Placebo	Olaparib	Placebo	Olaparib	Placebo	Olaparib	Placebo	Olaparib	Placebo
	N=	N=	N=	N=	N=	N=	N=	N=	N=	N=	N=	N=	N=	N=	N=	N=	N=	N=	N=	N=
**Longest Diameter of Target Tumours - n, median (IQR)**																				
Primary tumour																				
Lymph node																				
Brain																				
Bone																				
Liver																				
Adrenal glands																				
Other																				
**Sum of Longest Diameters - n, median (IQR)**																				
**Minimum SLD - n, median (IQR)**	NA	NA																		

Table 12: Objective response rate

Table 12: Objective Response	Overall at end of Cycle 2	Cycle 2		Cycle 4		Cycle 6		Cycle 8		Cycle 10		Cycle 12		Cycle 14		Cycle 16		Cycle 18	
		Olaparib	Placebo	Olaparib	Placebo	Olaparib	Placebo	Olaparib	Placebo	Olaparib	Placebo	Olaparib	Placebo	Olaparib	Placebo	Olaparib	Placebo	Olaparib	Placebo
	N=	N=	N=	N=	N=	N=	N=	N=	N=	N=	N=	N=	N=	N=	N=	N=	N=	N=	N=
**Response of target lesions - N (%)**																			
Complete response																			
Partial response																			
Stable disease																			
Progressive disease																			
Not evaluable																			
**Response of non-target lesions -**																			
Complete response																			
Non-CR/Non-PD																			
Progressive disease																			
Not evaluable																			
**Overall response - N (%)**																			
Complete response																			
Partial response																			
Stable disease																			
Progressive disease																			
Not evaluable																			

Table 13: Progression-free and overall survival

Table 13: Progression-free and overall survival	Olaparib	Placebo	HR (95% CI)	One-sided p-value
Progression-free survival (weeks) - n, median (95% CI)				
ITT population				
PP population				
Cox model adjusted				
Overall survival (months) - n, median (95% CI)				
ITT population				
Duration of follow up (weeks) - n, median (IQR)				

Table 14: Patient withdrawals and deaths

Table 14: Patient withdrawal and death	Olaparib<br>N=	Placebo<br>N=
**Withdrawal - N (%)**		
Level 1		
Level 2		
Level 3		
Level 4		
Total		
**Reasons for withdrawal from trial treatment - N (%)**		
Participant choice		
Intolerance to treatment		
Non-compliance		
Lost to follow up		
Clinical disease progression		
Development of MDS or AML		
Incorrect enrolment		
Other1		
Other2		
Other3		
Total		
**Death - N (%)**		
Disease progression		
Treatment related toxicity		
Other1		
Other2		
Other3		
Total		

Table 15: EudraCT reportable non-serious adverse events

Table 15: EudraCT reportable non-serious adverse events (>5% of patients experience the AE)	Subjects - N (%)		Occurrences - N (%)	
	Olaparib	Placebo	Olaparib	Placebo
	N=	N=	N=	N=
**Blood and lymphatic system**				
Anaemia - N (%)				
Lymphopenia - N (%)				
Neutropenia - N (%)				
Thrombocytopenia - N (%)				
**Cardiac disorders**				
Tachycardia - N (%)				
**Gastrointestinal disorders**				
Abdominal distension - N (%)				
Abdominal pain - N (%)				
Constipation - N (%)				
Diarrhoea - N (%)				
Dry mouth - N (%)				
Dyspepsia - N (%)				
Flatulence - N (%)				
Mucosal inflammation - N (%)				
Nausea - N (%)				
Vomiting - N (%)				
**General disorders and administration site conditions**				
Fatigue - N (%)				
Fever - N (%)				
Oedema peripheral - N (%)				
Palmar-plantar syndrome - N (%)				
**Infections and infestations**				
Lower respiratory infection - N (%)				
Upper respiratory infection - N (%)				
Urinary tract infection - N (%)				
Psychiatric disorders - N (%)				
**Metabolism and nutrition disorders**				
Anorexia - N (%)				
Hyponatraemia - N (%)				

Table 15: EudraCT reportable non-serious adverse events (>5% of patients experience the AE)	Subjects - N (%)		Occurrences - N (%)	
	Olaparib	Placebo	Olaparib	Placebo
	N=	N=	N=	N=
**Musculoskeletal and connective tissue disorders**				
Arthralgia - N (%)				
Back pain - N (%)				
Nervous system disorders - N (%)				
Dizziness - N (%)				
Dysgeusia - N (%)				
Headache - N (%)				
Nasopharyngitis - N (%)				
**Psychiatric disorders**				
Insomnia - N (%)				
**Respiratory, thoracic and mediastinal disorders**				
Coughing - N (%)				
Dyspnoea - N (%)				
Oropharyngeal pain - N (%)				
Pneumonitis - N (%)				
Stomatitis - N (%)				
**Skin and subcutaneous tissue disorders**				
Alopecia - N (%)				
Blisters - N (%)				
Erythema - N (%)				
Rash - N (%)				
**Neoplasms benign, malignant and unspecified**				
AML - N (%)				
Pruritis - N (%)				
Myelodysplastic syndrome - N (%)				
Vascular disorders - N (%)				
Hot flushes - N (%)				
Hypertension - N (%)				
**Other1, specify with CTCAE category - N (%)**				
**Other2, specify with CTCAE category - N (%)**				
**Other3, specify with CTCAE category - N (%)**				
**etc**				

Table 16: EudraCT reportable Serious Adverse Events

Table 16: EudraCT reportable Serious Adverse Events	Subjects - N (%)		Occurrences - N (%)		Occurrences related to treatment - N (%)		Deaths - N (%)		Deaths related to treatment - N (%)	
	Olaparib	Placebo	Olaparib	Placebo	Olaparib	Placebo	Olaparib	Placebo	Olaparib	Placebo
	N=	N=	N=	N=	N=	N=	N=	N=	N=	N=
System organ classA (specify) - N(%)										
Preferred term1										
Preferred term2										
etc.										
System organ classB (specify) - N(%)										
Preferred term1										
Preferred term2										
etc.										
System organ classC (specify) - N(%)										
Preferred term1										
Preferred term2										
etc.										
